# Effect of rPET Content and Preform Heating/Cooling Conditions in the Stretch Blow Molding Process on Microcavitation and Solid-State Post-Condensation of vPET-rPET Blend: Part II—Statistical Analysis and Interpretation of Tests

**DOI:** 10.3390/ma18010036

**Published:** 2024-12-25

**Authors:** Paweł Wawrzyniak, Waldemar Karaszewski, Marta Safandowska, Rafał Idczak

**Affiliations:** 1 Faculty of Automotive and Construction Machinery Engineering, Warsaw University of Technology, 84 Ludwika Narbutta Street, 02-524 Warsaw, Poland; 2 Faculty of Mechanical Engineering and Ship Technology, Gdansk University of Technology, 11/12 Gabriela Narutowicza Street, 80-233 Gdansk, Poland; waldemar.karaszewski@pg.edu.pl; 3 Centre of Molecular and Macromolecular Studies, Polish Academy of Sciences, 112 Sienkiewicza Street, 90-363 Lodz, Poland; marta.safandowska@cbmm.lodz.pl; 4 Faculty of Physics and Astronomy, University of Wroclaw, 9 Maxa Born Square, 50-204 Wroclaw, Poland; ridczak@ifd.uni.wroc.pl

**Keywords:** PET recycling, SBM process, microcavitation, solid-state post-condensation (SSPC), power of ANOVA test in DOE, response surface methodology (RSM), positron annihilation lifetime spectroscopy (PALS)

## Abstract

This research explores how varying proportions of virgin polyethylene terephthalate (vPET) and recycled polyethylene terephthalate (rPET) in vPET-rPET blends, combined with preform thermal conditions during the stretch blow molding (SBM) process, influence PET bottles’ microscopic characteristics. Key metrics such as viscosity, density, crystallinity, amorphous phase relaxation, and microcavitation were assessed using response surface methodology (RSM). Statistical analysis, including Analysis of variance (ANOVA) and its power, supported the interpretation of results. The first part of the work details the experimental design and statistical methods. Positron annihilation lifetime spectroscopy (PALS) and amorphous phase density analysis revealed reduced free volume size, a substantial increase in free volume quantity, and a transformation toward ellipsoidal geometries, highlighting significant structural changes in the material. At the same time, the intrinsic viscosity (IV) and PALS studies indicate that the solid-state post-condensation effect (SSPC) is linked with microcavitation through post-condensation product diffusion. The conclusions, which resulted from the microstructure analysis, affected the material’s mechanical strength and were validated by pressure resistance tests of the bottles.

## 1. Introduction

In recent years, environmental protection has become increasingly important from the point of view of the reuse of polymer materials. This is particularly important in the case of a circular economy [1], i.e., one in which products, materials, and raw materials should remain in circulation as long as possible, and waste generation should be minimized as much as possible. Part of this trend is the packaging of carbonated and non-carbonated beverages in PET bottles that contain recycled PET (rPET). The rPET content causes significant changes in the internal structure of PET after the stretch blow molding (SBM) process (forming bottles/packages), and this structure is closely related to its mechanical, physical, and chemical properties. We must emphasize that this study analyzed only pure PET (virgin and recycled), while the combination of different recycled PET formulations with other materials to create composites [2] could enhance their nanomechanics [3]. However, this issue will not be discussed in this study.

A broad review of the literature on the SBM process (from preforms containing vPET and rPET) with cold and hot molds was presented in other articles [4,5,6,7,8,9,10,11,12,13,14]. This paper aims to describe and interpret the results of statistical analysis of the influence of rPET content, preform heating power, and preform cooling power in the SBM process on the microscopic bottle and preform material properties. The microscopic bottle and preform material properties were defined by the degree of crystallinity, density, intrinsic viscosity, relaxation of the amorphous phase, and microcavitation in PET. For this purpose, response surface experiments were performed for the analyzed factors (independent variables), i.e., rPET content, the power of heating lamps, the power of cooling fans, and the SBM process as a whole. Analysis of variance (ANOVA) and power of ANOVA in the design of experiments (DOE) were carried out. The sample size was based on the authors’ previous research [15,16].

Due to the large scope of the research, the entire work was divided into two parts to increase its clarity. The first part of our work [17] focused on the description of the purpose and methodology of the research, with the presentation of research results on the microscopic and macroscopic properties of bottle and preform material. In addition, a literature review was developed in a separate article [18], which discussed in detail the current knowledge on the phenomena of cavitation and post-condensation occurring in the PET material. The literature review included the three-phase PET model (an exemplary in-depth study performed by combining the conventional Differential Scanning Calorimetry (DSC) with the Fast Scanning Calorimetry (FSC) of the MAF and RAF structures of PET material was also presented by Heidrich and Gehde [19]), the cavitation process, and the solid-state post-condensation process occurring in the PET material. Based on the literature review [18], a causal mechanism was developed linking the phenomenon of cavitation and solid-state post-condensation, which may occur in the SBM process, i.e., at temperatures much lower than the melting point of PET. The literature review revealed the five potential hypotheses, with the first two being contradictory due to the influence of rPET content on the microcavitation process [18]. These two are verified in this part of the study, which are as follows:

**Hypothesis** **1.** “*The addition of rPET reduces the occurrence of microcavitation and, consequently, post-condensation because the crystallites with the addition of rPET are less perfect, and due to this, microcavitation decreases as well as the efficiency of the post-condensation process in the vicinity of the volume of voids (“cavitation is prevented in SCPs possessing thinner or more defective crystals or lower molecular weight”* [20]*).”*

**Hypothesis** **2.** “*Or, the addition of rPET increases the trans conformation content in the amorphous phase (which increases the stiffness of this phase), which reduces the mobility of the free ends of PET macromolecules, reducing the efficiency of the PET solid-state post-condensation process inside, but without reducing the microcavitation effects—for increasing the amount of rigid amorphous structures, the Tg increases for the material* [21] *and the shift of Tg towards higher values hinders the migration of macromolecule fragments/chain ends (this should further hinder/inhibit the post-condensation process).”*

The main purposes of this paper are:

1. The DOE and power of ANOVA tests’ analysis for the microscopic and macroscopic bottle and preform properties;
2. Interpretation of data regarding the degree of crystallinity, relaxation of the amorphous phase, microcavitation measured by PALS, and solid-state post-condensation measured by intrinsic viscosity;
3. Definition of conclusions concerning microcavitation phenomena confirmed by annihilation positron measurements and solid-state post-condensation phenomena confirmed by intrinsic viscosity (verify the above two hypotheses).

## 2. Research Methodology

### 2.1. Research Plan

**Figure A1:**
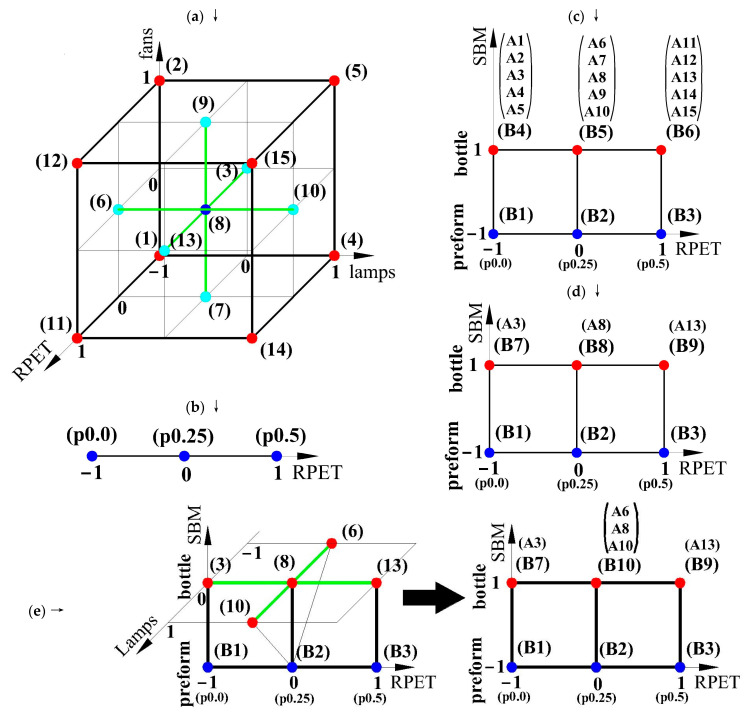

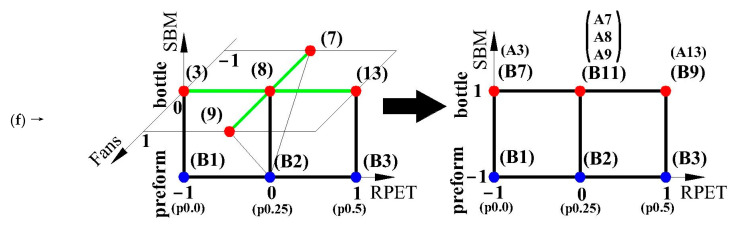
Visual depiction of the experimental designs outlined in Table A1, where a detailed description of the figures (**a**–**f**) is presented in the previous paper in Figure A1 [17].

In the first part of our work [17], we present the experimental plans for examining the impact of rPET content on the microstructure of the preform material (Table 1b [17]), the impact of rPET content in the preform and the SBM process itself on the microstructure of the bottle material in relation to the preform material (Table 1c [17] for “ALL”, Table 1d [17] for “RPET”, Table 1e [17] for “change in the impact of RPET content from LAMPS”, Table 1f [17] for “change in the impact of RPET content from FANS”), and the study of the impact of rPET content, power of heating lamps, and power of cooling fans in the CCF plan on the microstructure and macroscopic properties of the bottle material in the SBM process (Table 1a [17]). In order to facilitate understanding of the methodology used, Table A1 (Table 1 from the first part of the paper [17]) and Figure A1 (from the first part of the paper [17]) have been presented in Appendix A. A detailed description of Table A1 and Figure A1 can be found in the first part of this paper [17].

The microstructure of preform and bottle material is defined by density (ρ), intrinsic viscosity (IV), thermal properties (DSC), and free volume (PALS). At the same time, the macroscopic properties of the bottle material are defined by the thickness profiles (TH) of the bottle wall (at three points: I, II, and III) and the pressure resistance (PRT) of the bottle.

### 2.2. Materials, Reagents, and Method Used

Used materials, reagents, and methods were presented in the first part of our work [17].

### 2.3. Statistical Methodology

**Figure 1:**
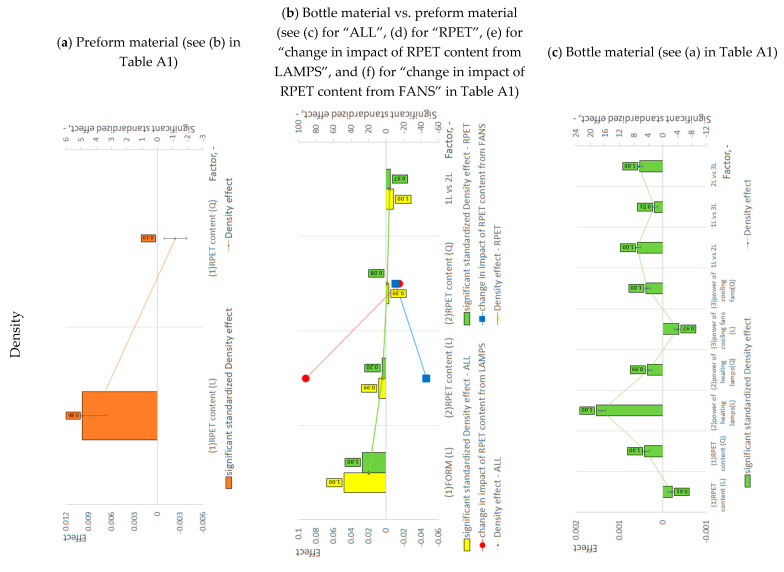

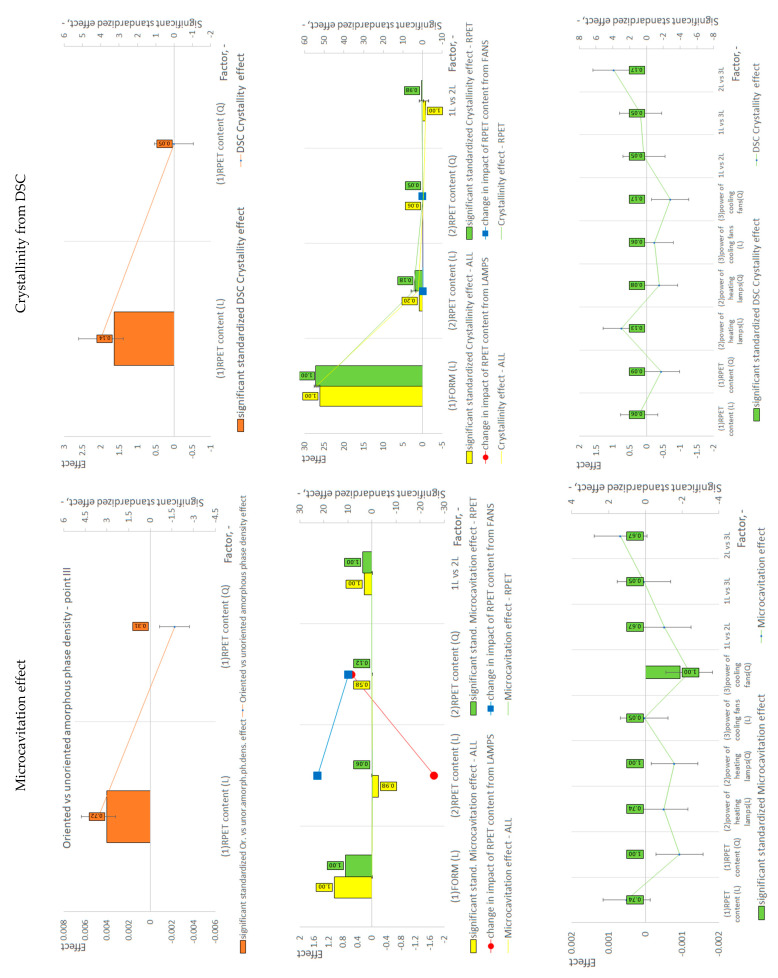

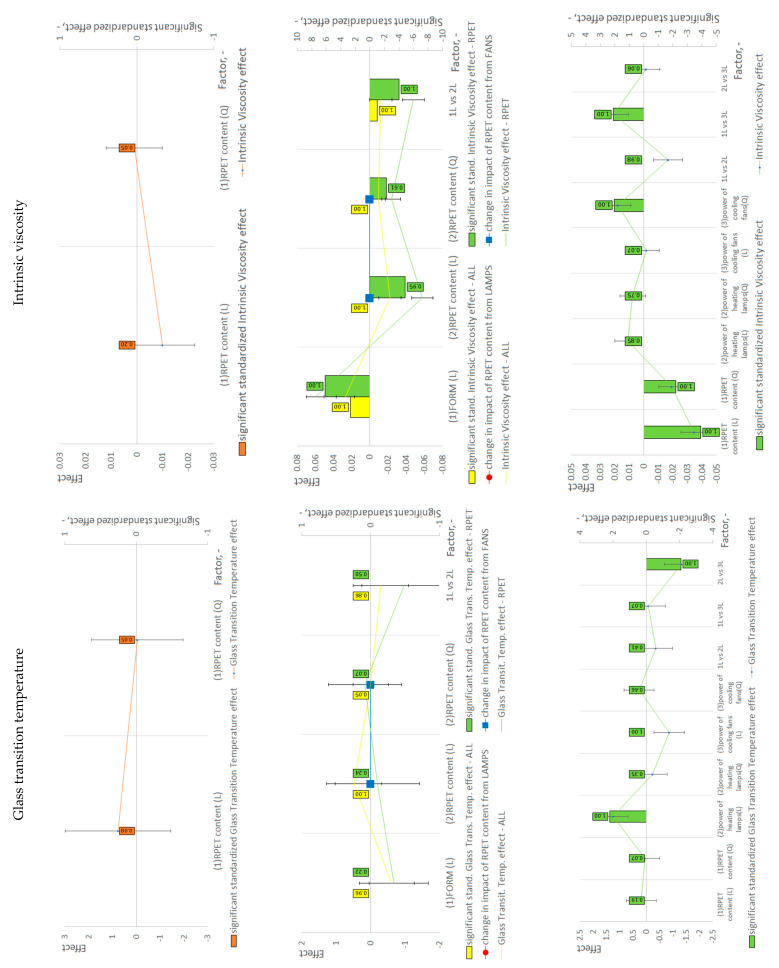

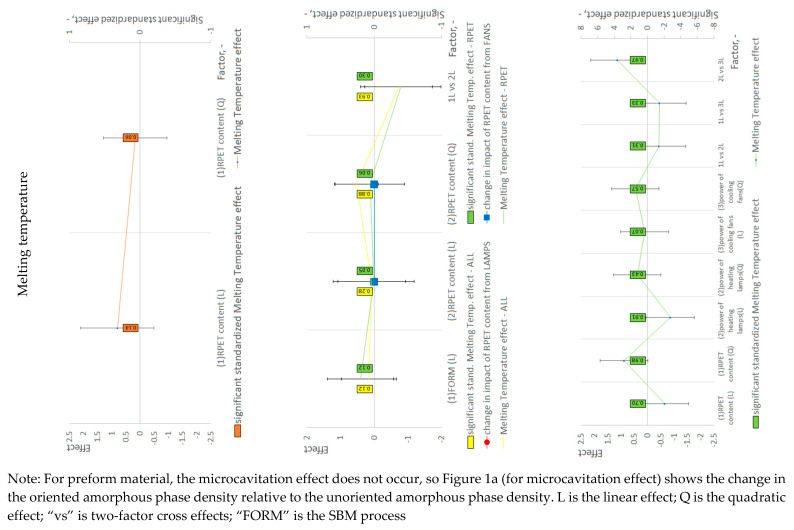
Results of the statistical analyses regarding the impact on the physical and thermal properties of: (**a**) RPET content on the preform material; (**b**) RPET content and SBM process on the bottle material in relation to the preform material (“ALL” refers to testing rPET content for variable power of heating lamps and the power of cooling fans, while “RPET” refers to testing the influence of RPET content for the constant power of heating lamps and cooling fans (see description in Part I [17])); (**c**) RPET content, power of heating lamps, and power of cooling fans on the bottle material after the SBM process. An example of calculating effects is provided in Appendix B using the density measurement example. The calculations of effects for all other dependent variables are analogous. The unoriented amorphous phase density for PET is 1.3350 g/cm^3^ [27].

**Figure 2:**
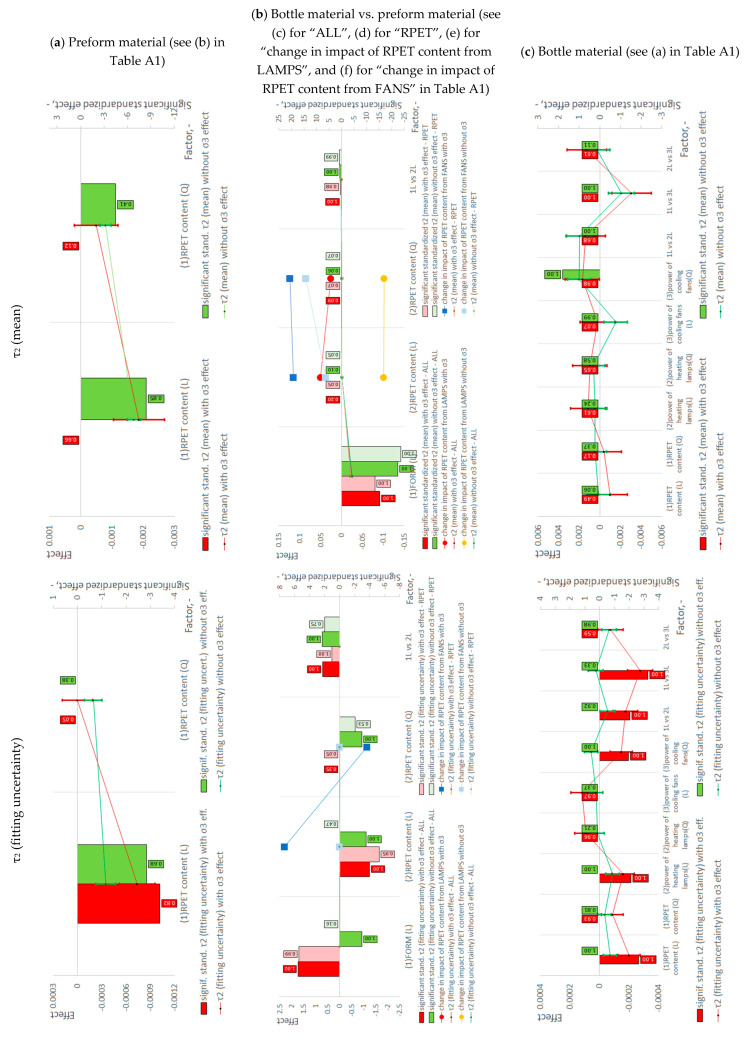

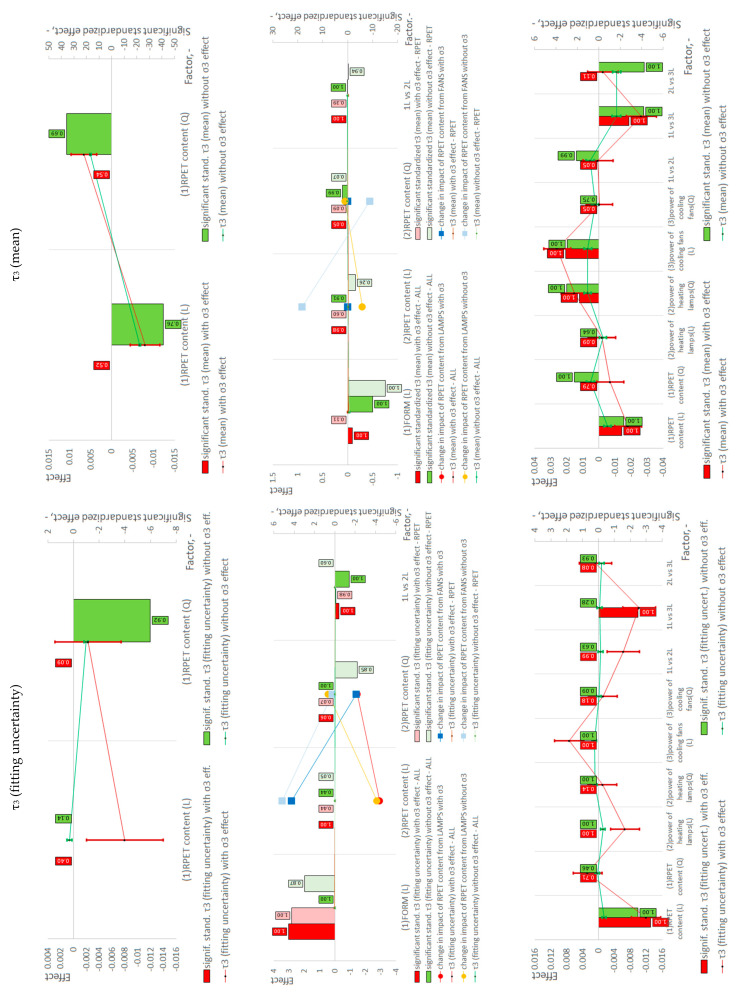

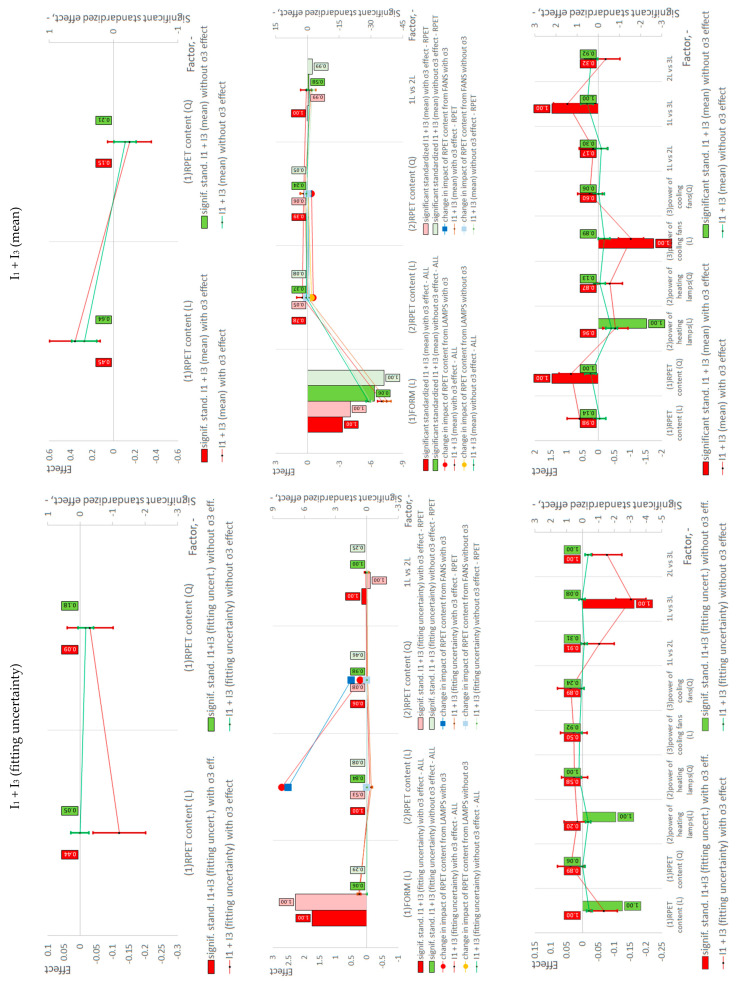

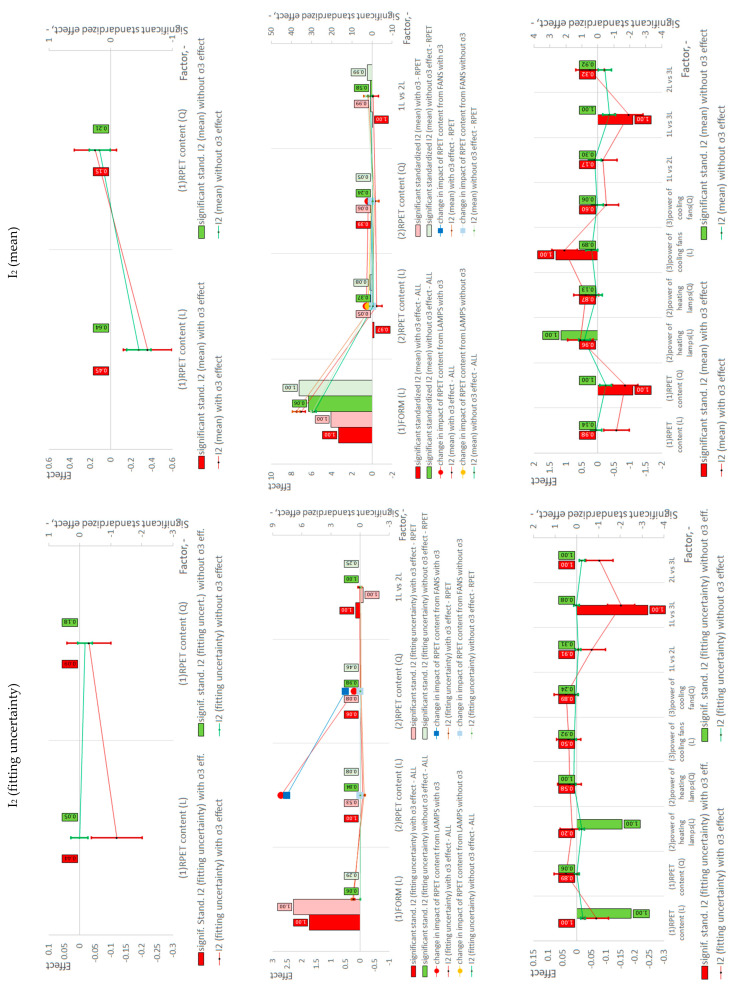

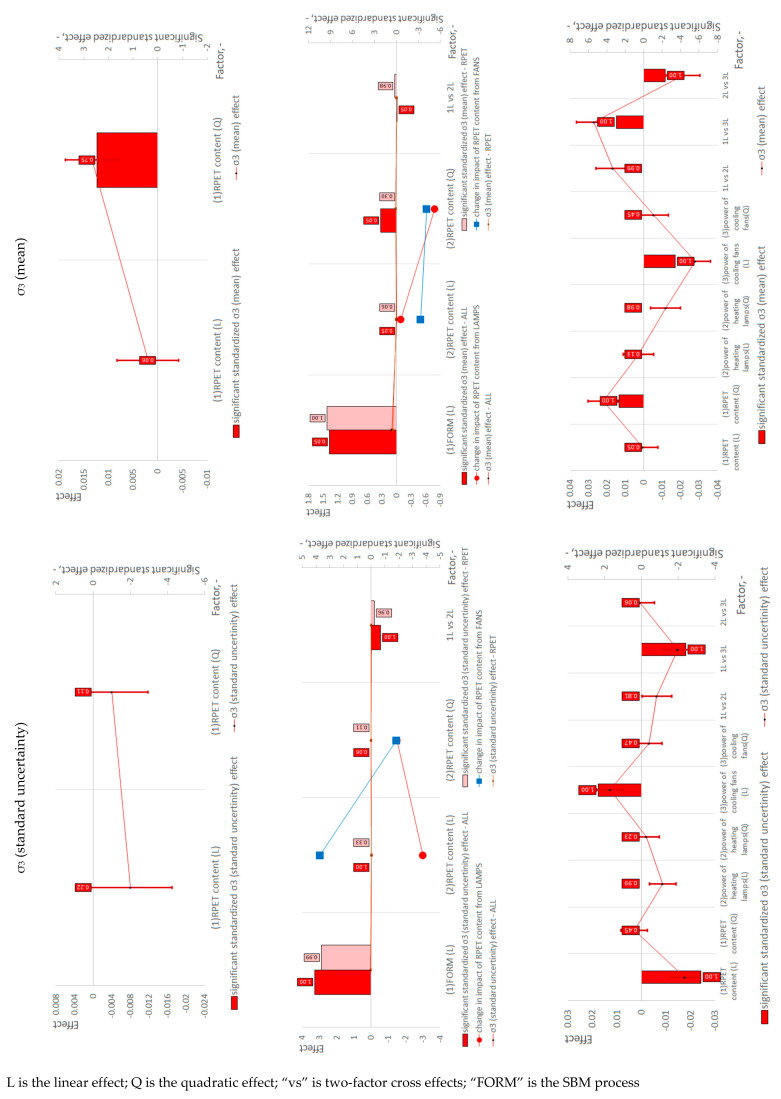
Results of the statistical analyses regarding the impact on the PALS of: (**a**) RPET content on the preform material; (**b**) RPET content and SBM process on the bottle material in relation to the preform material (“ALL” refers to testing rPET content for the variable power of heating lamps and cooling fans, while “RPET” refers to testing the influence of rPET content for the fixed power of heating lamps and cooling fans (see description in Part I [17])); (**c**) RPET content, power of heating lamps, and power of cooling fans on the bottle material after the SBM process. An example of calculating effects is provided in Appendix B using the density measurement example. The calculations of effects for all other dependent variables are analogous.

**Figure 3:**
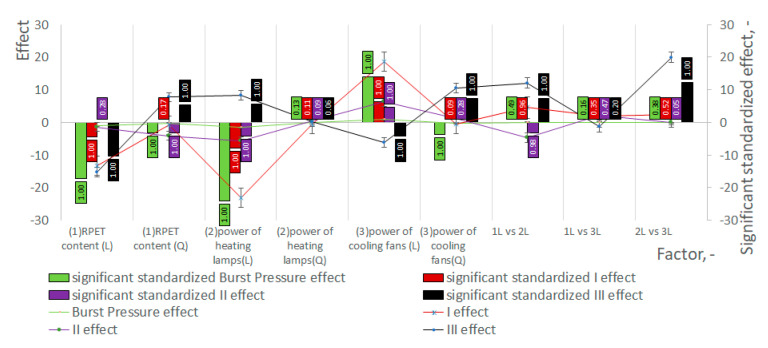
Results of the statistical analyses of macroscopic properties of bottles.

A statistical analysis methodology was also presented in the first part of our work [17]. However, to facilitate the understanding of the statistical methodology used, Appendix B presents the steps for creating graphs shown in Figure 1a–c for the example of density analysis. The steps for developing these graphs for all other dependent variables (Figure 1, Figure 2 and Figure 3) are analogous.

Appendix C presents the steps for developing Table 1 and Table 2 for the example of interpretation of the effect of independent variables on density (shown in Figure 1a–c). The steps for interpretation graphs from Figure 1, Figure 2 and Figure 3 for all other dependent variables (Table 1 and Table 2) are analogous. 

Statistical calculations were performed using the Statistica 13 software (StatSoft Polska Sp. z o.o.: Krakow, Poland).

## 3. Results

### 3.1. Measurements Results

Measurements results of the dependent variables mentioned in Table A1 (density (ρ), intrinsic viscosity (IV), thermal properties (DSC), free volume (PALS), thickness profiles (TH) of the bottle wall (at three points: I, II, and III), and the pressure resistance (PRT)) were presented in the first part of our work in Tables S4–S8 [17].

### 3.2. Statistical Analysis Results

Figure 1a–c illustrate the results of statistical analysis conducted to assess the effects of rPET content on the microstructure of the preform material, the influence of rPET content in both the preform and the SBM process on the microstructure of the bottle material compared to the preform, and the impact of rPET content, heating lamp power, and cooling fan power within the CCF plan on the microstructure of the bottle material during the SBM process. The microstructure of both the preform and the bottle was evaluated through viscosity, density, and calorimetric measurements.

Due to the occurrence of post-condensation phenomena [17], the microstructural investigation, initially based on DSC thermograms and density measurements, was expanded to incorporate PALS testing. The PALS results, analyzed in conjunction with the DSC and density data, demonstrated consistent correlations. Room-temperature PALS spectra were processed using LT-9.0 software [22], employing two distinct models.

The first model, termed “with dispersion”, assumes that each PALS spectrum can be resolved into three components: p-Ps annihilation (defined by τ_1_ = 125 ps and I_1_), free positron annihilation (characterized by τ_2_ and I_2_), and o-Ps pick-off annihilation (described by τ_3_, σ_3_, and I_3_). Here, τ_i_ and I_i_ represent the mean positron lifetime and the relative intensity of the i-th spectrum component, respectively. The I_1_/I_3_ ratio was fixed at 1/3. The longest component, τ_3_, was modeled as a continuous distribution, with τ_3_ and σ_3_ representing the average lifetime and its dispersion. In polymeric systems, variations in τ_3_ reflect the distribution of free volume. The second model, called “without dispersion”, mirrors the first but sets the σ_3_ parameter to zero. Both models are widely applied in the analysis of PALS spectra for polymers [23,24,25,26].

Figure 2a–c show the results of statistical tests for the study of the impact of rPET content on positron annihilation analysis in the preform material, the effect of rPET content and the SBM process itself on positron annihilation in the bottle material relative to the preform material, and the study of the effect of rPET content, power of heating lamps, and power of cooling fans in the CCF plan on positron annihilation in the bottle material in the SBM process. Figure 2a–c show the variation of lifetimes (τ_2_ and τ_3_) and their intensities for models that take into account the σ_3_ dispersion of the spectrum and that do not take into account the σ_3_ dispersion of the spectrum.

Figure 3 shows results of the statistical analyses of macroscopic properties of bottles. The tests of macrostructural properties of the bottles (wall thickness at points I, II, III and pressure resistance of the bottles) were performed in order to validate the conclusions resulting from the analysis of the microstructure of the bottle material.

Figure 1, Figure 2 and Figure 3 show the statistically significant standardized effect with the axis on the right (values of statistically insignificant effects were given as zero) and the absolute effect with the axis on the left with the standard error bars of the given effect. The numbers in the boxes above the bars indicate the power of the statistical test. It should be noted that the power of tests for almost all statistically insignificant effects is less than 80%; therefore, the results obtained cannot be used for quantitative analysis but can be employed for a preliminary qualitative analysis (see Table S7). Appendix B presents the calculation of effects and their statistical significance using density as an example (Appendix B presents the procedure for generating the graphs shown in Figure 1a–c). The numerical results of standardized effects, *p*-values in ANOVA, power of ANOVA tests, and adjusted R^2^ parameter for all experimental designs (Table A1) of all dependent variables are presented in Table S7. All charts in Figure 1, Figure 2 and Figure 3 were made using Microsoft Excel 365 software (Microsoft Corporation: Washington, DC, USA).

## 4. Interpretation of Statistical Results

The interpretation of the statistical results of the microscopic (Figure 1 and Figure 2) and macroscopic (Figure 3) features tests is presented symbolically in Tables S1–S6 (included in Supporting Information), and in Appendix C using the example of density. The physical equations employed to carry out the statistical studies are described in detail in Appendix B and Appendix C and based on the example of density measurement. The verbal interpretation of Tables S1–S6 requires a large volume of text; therefore, the full statistical interpretation of Figure 1, Figure 2 and Figure 3 used to create Tables S1–S6 will be sent on request.

Table 1 summarizes the analyzed linear effects shown in Tables S1, S2 and S5. Data analysis of PALS analysis, taking into account the dispersion parameter σ_3_, enables the analysis of shape dispersion of the analyzed structure in the sample volume. Therefore, the results for mean time τ_2_ and τ_3_ were given without taking into account σ_3_ dispersion, and then from those times, it can be found information about the average dimensions of the analyzed structures in the sample volume. For the same reason, the fitting uncertainty (δτ_2_ and δτ_3_) was given along with the σ_3_ dispersion, and then from them, it can be found information about the regularity of mean dimension distribution (in the text, it is called briefly regularity) of the analyzed structures in the sample volume—the greater the fitting uncertainty, the less and less regularly distributed the mean dimensions of the structure become in the sample volume. However, the σ_3_ dispersion itself indicates the ellipsoidality of the free volumes, and the higher the σ_3_ dispersion, the greater the ellipsoidality of the free volumes in the sample volume. Ellipsoidality is different from the regularity of mean dimension distribution (regularity), because ellipsoidality concerns the individual dimensions of structures in the sample volume, and regularity concerns the distribution of mean dimensions in the sample volume.

An analysis of quadratic effects (Tables S1, S3 and S6) reveals four distinct scenarios based on the statistical significance of the quadratic effect and its magnitude relative to the linear effect [17]:

1. No non-linearity: The quadratic effect is statistically insignificant.
2. Non-linearity without trend reversal: The quadratic effect is statistically significant, but its magnitude does not exceed ¼ of the linear effect’s magnitude, meaning the trend direction in the dependent variable remains consistent with changes in the independent variable.
3. Non-linearity with ambiguous trend behavior: The quadratic effect is statistically significant, with its magnitude exceeding ¼ but remaining below ½ of the linear effect’s magnitude, suggesting no definitive trend reversal in the dependent variable.
4. Non-linearity with trend reversal: The quadratic effect is statistically significant and its magnitude surpasses ½ of the linear effect’s magnitude, indicating a reversal in the trend direction of the dependent variable relative to the independent variable.

Table 2 categorizes dependent variables based on their linear or non-linear relationship with independent variables.

**Table 1:** Summary of the analyzed main linear effects shown in Tables S1, S2 and S5.

Dependent Variables			Independent Variables						
			SBM Process	RPET Content		Power of Heating Lamps		Power of Cooling Fans	
Preform (Table S1)	Physical and thermal properties	Density	N/A	+		N/A		N/A	
		Crystallinity	N/A	+		N/A		N/A	
		Oriented amorphous phase	N/A	+		N/A		N/A	
		Intrinsic viscosity	N/A	0		N/A		N/A	
		*T*_g_	N/A	0		N/A		N/A	
		*T*_m_	N/A	0		N/A		N/A	
	PALS analysis	τ_2_ (mean) without σ_3_	N/A	−		N/A		N/A	
		τ_2_ (fitting uncertainty) with σ_3_	N/A	−		N/A		N/A	
		τ_3_ (mean) without σ_3_	N/A	−		N/A		N/A	
		τ_3_ (fitting uncertainty) with σ_3_	N/A	0		N/A		N/A	
		I_1_ + I_3_ (mean)	N/A	0		N/A		N/A	
		I_1_ + I_3_ (fitting uncertainty)	N/A	0		N/A		N/A	
		I_2_ (mean)	N/A	0		N/A		N/A	
		I_2_ (fitting uncertainty)	N/A	0		N/A		N/A	
		σ_3_ (mean)	N/A	0		N/A		N/A	
		σ_3_ (standard uncertainty)	N/A	0		N/A		N/A	
Bottle vs. preform (Table S2)	Physical and thermal properties	Density	+	Preform +	Bottle 0	N/A		N/A	
		Crystallinity	+	+		N/A		N/A	
		Microcavitation effect	+	Preform −	Bottle +	N/A		N/A	
		Intrinsic viscosity	+	−		N/A		N/A	
		*T*_g_	0	0		N/A		N/A	
		*T*_m_	0	0		N/A		N/A	
	PALS analysis	τ_2_ (mean) without σ_3_	−	Preform −	Bottle +	N/A		N/A	
		τ_2_ (fitting uncertainty) with σ_3_	+	−		N/A		N/A	
		τ_3_ (mean) without σ_3_	−	−		N/A		N/A	
		τ_3_ (fitting uncertainty) with σ_3_	+	Preform +	Bottle −	N/A		N/A	
		I_1_ + I_3_ (mean)	−	Preform +	Bottle −	N/A		N/A	
		I_1_ + I_3_ (fitting uncertainty)	+	Preform +	Bottle −	N/A		N/A	
		I_2_ (mean)	+	Preform −	Bottle +	N/A		N/A	
		I_2_ (fitting uncertainty)	+	Preform +	Bottle −	N/A		N/A	
		σ_3_ (mean)	+	Preform −	Bottle +	N/A		N/A	
		σ_3_ (standard uncertainty)	+	Preform +	Bottle −	N/A		N/A	
Bottle (Table S5)	Physical and thermal properties	Density	N/A	LAMPS − | +	FANS − | 0	+		RPET −	LAMPS − | +
		Crystallinity	N/A	0		0		0	
		Microcavitation effect	N/A	0		0		0	
		Intrinsic viscosity	N/A	−		0		RPET − | +	LAMPS 0
		*T*_g_	N/A	0		RPET +	FANS + | 0	RPET 0	LAMPS + | −
		*T*_m_	N/A	0		0		0	
	PALS analysis	τ_2_ (mean) without σ_3_	N/A	0		0		0	
		τ_2_ (fitting uncertainty) with σ_3_	N/A	LAMPS −	FANS + | −	RPET 0 | −	FANS −	RPET + | −	LAMPS 0
		τ_3_ (mean) without σ_3_	N/A	LAMPS − | 0	FANS + | −	RPET − | +	FANS + | −	RPET + | −	LAMPS + | −
		τ_3_ (fitting uncertainty) with σ_3_	N/A	−		0		RPET + | −	LAMPS 0
		I_1_ + I_3_ (mean)	N/A	0		−		0	
		I_1_ + I_3_ (fitting uncertainty)	N/A	LAMPS 0	FANS − | +	0		RPET − | +	LAMPS 0
		I_2_ (mean)	N/A	0		+		0	
		I_2_ (fitting uncertainty)	N/A	LAMPS 0	FANS + | −	0		RPET + | −	LAMPS 0
		σ_3_ (mean)	N/A	LAMPS 0	FANS − | +	RPET 0	FANS + | −	RPET − | 0	LAMPS −
		σ_3_ (standard uncertainty)	N/A	−		0		RPET + | 0	LAMPS +
	Macroscopic properties	Thickness-I	N/A	−		−		+	
		Thickness-II	N/A	LAMPS + | −	FANS 0	−		+	
		Thickness-III	N/A	−		+		−	
		Pressure resistance	N/A	−		−		+	

where “+” is the dependent variable increasing as the independent variable increases; “−” is the dependent variable decreasing as the independent variable increases; “0” is the dependent variable not changing statistically significantly as the independent variable increases; “− | +” is the change in the dependent variable due to a change in the independent variable conditioned by the value of the “other” independent variable, and in the designation, the left side shows the effect of a change in the independent variable on the dependent variable, with a minimum setting of the “other” independent variable, while the right side shows the effect of a change in the independent variable on the dependent variable, at the maximum setting of the “other” independent variable, i.e., “min | max”; N/A means “not applicable”.

**Table 2:** Division of dependent variables due to linearity and non-linearity of changes concerning independent variables shown in Tables S1, S3 and S6.

Object	Independent Variable	Trend of Changes in the Dependent Variable			
		1. Linear Variability of the Dependent Variable in Terms of the Independent Variable	Non-Linear Variation of the Dependent Variable Within the Range of the Independent Variable		
			2. No Change in the Sign of the Trend of Changes in the Dependent Variable	3. No Clear Evidence of a Change in the Sign of the Trend of Changes in the Dependent Variable	4. Change in the Sign of the Trend of Changes in the Dependent Variable
Preform (Table S1)	rPET content	(1) Density (+;0) (2) Crystallinity (+;0) (3) Density of amorphous phase (+;0) (4) τ_2_ (fitting uncertainty) with σ_3_ (−;0)	-	(1) τ_2_ (mean) without σ_3_ (−;−)	(1) τ_3_ (mean) without σ_3_ (−;+) (2) τ_3_ (fitting uncertainty) without σ_3_ (0;−) (3) σ_3_ mean (0;+)
Bottle and Preform (Table S3)	rPET content	(1) Density “RPET” (+;0) (2) Crystallinity “ALL” (+;0), “RPET” (+;0) (3) Microcavitation effect “ALL” (−;0) (4) τ_2_ (fitting uncertainty) with σ_3_ “ALL” (−;0), “RPET” (−;0) (5) τ_3_ (mean) without σ_3_ “RPET” (−;0) (6) I_2_ (mean) with σ_3_ “ALL” (−;0), without σ_3_ “RPET” (+;0)—there is a change of sign for the linear effect	(1) Density “ALL” (+;−)	(1) Intrinsic viscosity “RPET” (−;−)	(1) τ_2_ (fitting uncertainty) without σ_3_ “ALL” (−;−), “RPET” (0;−) (2) τ_3_ (mean) without σ_3_ “ALL” (0;+) (3) τ_3_ (fitting uncertainty) without σ_3_ “RPET” (0;−) (4) σ_3_ (mean) “ALL (0;+)
Bottle (Table S6)	rPET content	(1) τ_2_ (fitting uncertainty) with σ_3_ (−;0) (2) τ_3_ (mean) with σ_3_ (−;0) (3,4) τ_3_ (fitting uncertainty) with and without σ_3_ (−;0) (5) I_1_ + I_3_ (fitting uncertainty) without σ_3_ (−;0) (6) I_2_ (fitting uncertainty) without σ_3_ (−;0) (7) σ_3_ (standard uncertainty) (−;0) (8) Thickness-I (−;0)	(1) Pressure resistance (−;−)	(1) Intrinsic viscosity (−;−) (2) Thickness-III (−;+)	(1) Density (−;+) (2) τ_3_ (mean) without σ_3_ (+;−) (3) I_1_ + I_3_ (mean) with σ_3_ (0;+) (4) I_2_ (mean) with σ_3_ (0;−) (5) σ_3_ (mean) (0;+) (6) Thickness-II (0;−)
	Power of heating lamps	(1) Glass Transition Temperature (+;0) (2) τ_2_ (fitting uncertainty) with σ_3_ (−;0) (3) I_1_ + I_3_ (mean) without σ_3_ (−;0) (4) I_1_ + I_3_ (fitting uncertainty) without σ_3_ (−;0) (5) I_2_ (mean) without σ_3_ (+;0) (6) I_2_ (fitting uncertainty) without σ_3_ (−;0) (7) Thickness-I (−;0) (8) Thickness-II (−;0) (9) Thickness-III (+;0) (10) Pressure resistance (−;0)	(1) Density (+;+)	-	(1,2) τ_3_ (mean) with and without σ_3_ (0;+)
	Power of cooling fans	(1,2) τ_3_ (mean) with and without σ_3_ (+;0) (3) I_1_ + I_3_ (mean) with σ_3_ (−;0) (4) I_2_ (mean) with σ_3_ (+;0) (5) σ_3_ (mean) (−;0) (6) σ_3_ (standard uncertainty) (+;0) (7) Thickness-I (+;0) (8) Thickness-II (+;0)	(1) Pressure resistance (+;−)	-	(1) Density (−;+) (2) Microcavitation effect (0;−) (3) Intrinsic viscosity (0;+) (4) τ_2_ (fitting uncertainty) with σ_3_ (0;−) (5) τ_2_ (mean) without σ_3_ (0;+) (6) Thickness-III (−;+)

where the symbols in parentheses refer to the sign of the effects (linear; quadratic).

**Figure 4:**
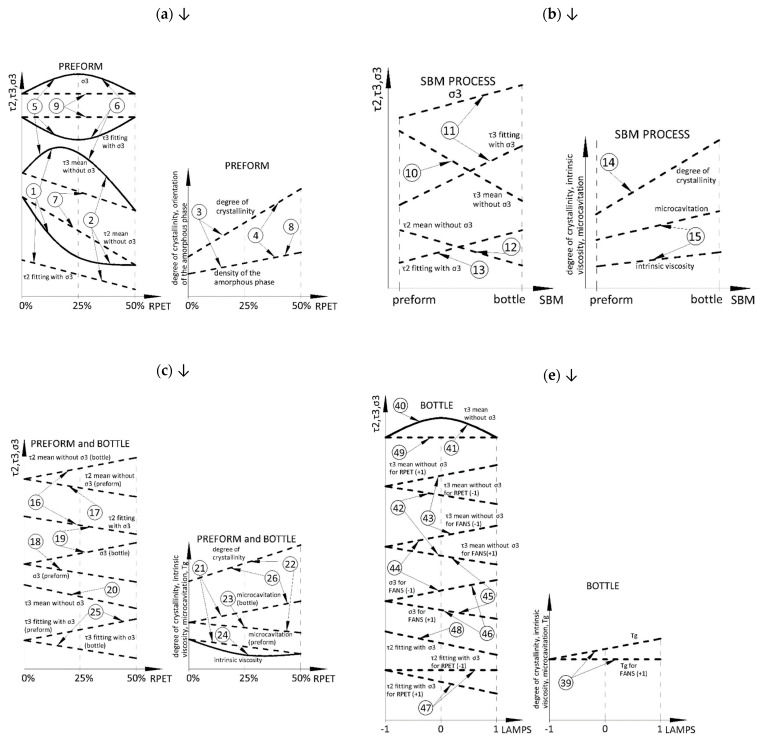

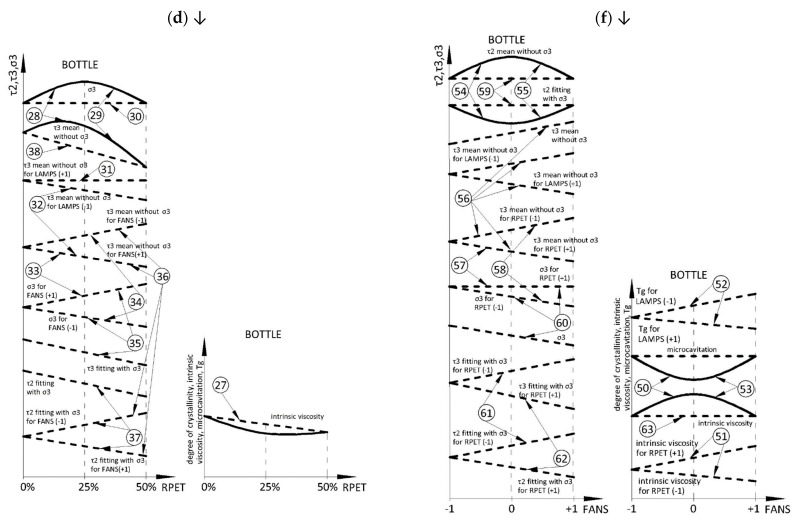
Conceptual graphic analysis of the linear and quadratic effects for dependent variables ($C_{DSC}$, M.E., IV, *T*_g_, *T*_m_, τ_2_, τ_3_, δτ_2_, δτ_3_, and σ_3_) of: (**a**) the preform material due to changes in the rPET content; (**b**) the bottle material in comparison with the preform material due to the SBM process; (**c**) the bottle material in comparison with the preform material due to changes in the rPET content; (**d**) the bottle material due to changes in the rPET content; (**e**) the bottle material due to changes in the power of heating lamps; (**f**) the bottle material due to changes in the cooling fans power. A detailed interpretation and description of all symbols in a circle 1–63 (described in Section 5 as “notes”) will be sent upon request.

A separate article contains a literature review [18] on which the statistical analysis of the obtained data is based. It must be noted that the density of the amorphous phase for preform material (note (8) in Figure 4a and note (23) in Figure 4c) is mainly related to the content of trans conformation in the amorphous phase (because the measured density of the amorphous phase is higher than the density of the unoriented amorphous phase—described in the first part of the work [17]), while the microcavitation in bottle material (notes (21), (22) in Figure 4c and notes (50), (53) in Figure 4f—which is calculated using information about the density of the amorphous phase) is mainly related to the content of the free volume in the amorphous phase (because the measured density of amorphous phase is lower than the density of unoriented amorphous phase—described in the first part of the work [17]).

Based on the data collected in Table 1 and Table 2, changes in dependent variables of the preform material due to changes in the rPET content, of the bottle material in comparison with the preform material due to the SBM process and changes in the rPET content, and of the bottle material due to changes in the rPET content, power of heating lamps, and cooling fans were conceptually illustrated in Figure 4a, Figure 4b, Figure 4c, Figure 4d, Figure 4e, and Figure 4f, respectively.

Figure 4 shows the designations 1–63 necessary for a full interpretation. A full interpretation of the research will be sent upon request to conduct a constructive dialogue because a complete interpretation contains aspects of speculation and requires the construction of separate research methodologies to verify them. Although the conclusions from the complete statistical analysis are consistent with the correlation analysis, they have yet to be published due to the lack of literature support. They refer to the last three hypotheses formulated in the literature review article [18]. This research and the designations used in Figure 4 (1–63) will be used in the future to verify these three hypotheses.

The following contains only an analysis related to the first two hypotheses formulated in the literature review article [18]: the comparison of vPET and rPET and the impact of rPET content on microstructure, which are proven by the research.

The abbreviations used in the description refer to: M.E.—microcavitation effect; $C_{DSC}$—crystallinity measured by DSC; IV—intrinsic viscosity; ρ—bulk density; ρ_a_—density of amorphous phase (the density of the non-crystalline phase, as a measure of the orientation of the amorphous phase); *T*_g_—glass transition temperature; *T*_m_—melting temperature; τ_2_—the free positron annihilation lifetime (proportional to the free annihilation in the material, often related to the annihilation in the crystalline parts); τ_3_—the ortho-positronium (o-Ps) pick-off annihilation lifetime (proportional to the free volumes space in the entire material, i.e., related to free spaces in the amorphous phase, at the border of the crystalline and amorphous phases, and in the crystalline phase); δτ_2_—the fitting uncertainties of τ_2_ (related to the regularity of crystallites dimensions due to the free volumes inside the crystallites); δτ_3_—the fitting uncertainties of τ_3_ (related to the regularity of the free volumes dimensions distribution in the sample volume); σ_3_—the spectrum dispersion parameter of lifetime τ_3_ (related to the ellipsoidality of the free volumes, and the higher the σ_3_ dispersion, the greater the ellipsoidality of the free volumes in the sample volume); Δσ_3_—the standard uncertainty of σ_3_ measurements.

## 5. Discussion

The following discussion is based on Figure 4 and refers to numbers in a circle 1–63. To improve the text flow, the term “numbers in a circle” has been replaced with the term “notes”.

### 5.1. Preform Material

For low values of rPET content, the density of the amorphous phase increases (notes (3) and (8)) with increasing of the average dimensions of the free volumes (note (1)—τ_3_ increases) and of their ellipsoidality (note (5)—σ_3_ increases), but there is also an increase in the regularity of the distribution of the average dimensions of free volumes (note (5)—δτ_3_ decreases). At the same time, the degree of crystallinity increases (note (3)) with a simultaneous increase in the average dimensions of the crystals (note (1)—τ_2_ decreases). It follows that the growth of crystal structures in the preform material, most likely caused by the “extraction” of the chains from the surrounding amorphous phase to the crystalline phase (RAF structures are formed [19]), increases the average dimensions of free volume and their ellipsoidality at the interface between the amorphous and crystalline phases.

However, for high values of rPET content, the density of the amorphous phase increases even stronger (note (4) and (8)—these can be related to the occurrence of a higher amount of trans conformations in the amorphous phase and thus a higher stiffness of amorphous phase, but also a greater probability of self-nucleation) with strong decreasing of the average dimensions of the free volumes (note (2)—τ_3_ decreases sharply) and of their ellipsoidality (note (5)—σ_3_ decreases), but a decrease in the regularity of the distribution of the average dimensions of free volumes (note (6)—δτ_3_ increases). At the same time, the degree of crystallinity increases (note (4)) with no change in the average dimensions of the crystals (note (2)—τ_2_ does not change). **This may be due to the fact that much higher rPET content involves not the growth of crystallites but causes the formation of more numerous crystallites with little change in their dimensions (therefore, despite the increase in the degree of crystallinity measured by DSC, the lifetime τ_2_ does not change, which proves the formation of more numerous crystallites), which takes place without “pulling” macromolecules from the surrounding amorphous phase (because the density of the amorphous phase also increases what can be related to the occurrence of higher trans conformations in the amorphous phase and thus a higher stiffness of amorphous phase).** So, it can be concluded that ordered macromolecules in the amorphous phase (which is measured by the density of the non-crystalline phase) arise as a result of the self-nucleation process in the amorphous phase, which is stronger for higher rPET content due to the larger amount of trans conformations of chains with higher rPET content.

The general conclusion from the obtained data shown in Figure 4a is that **the higher the rPET content in the preform, the stronger the self-nucleation of crystal structures taking place in the material (due to the greater number of trans-chain conformations for higher rPET content). Therefore, for low rPET content, fewer but larger crystallites are formed in the preform, and for high rPET content, more numerous but smaller crystallites are formed in the preform. This can be caused by the fact that rPET is characterized by an increased amount of the trans conformation of the chain in the non-crystalline phase, whose conformation may cause the crystallization nucleation.** So, in the mechanical (material) recycling process for low melting temperature, it is impossible to remove full information about the history of phase transformations of PET [28].

### 5.2. SBM Process

The SBM process itself and the increase in rPET content simultaneously increase the density (Table 1) of the material and the degree of crystallinity (note (14)). In the SBM process, both the density and the degree of crystallinity increase for the bottle material relative to the preform material. The increase in density is greater for 0% rPET content than for 50% rPET content. In contrast, the increase in the degree of crystallinity is greater for 50% rPET content than for 0% rPET content. **This inverse relationship between density and the degree of crystallinity indicates that for high rPET contents, more numerous but less perfect crystallites are formed compared to no rPET content.** What is more, in the SBM process, δτ_2_ increases (note (13)) but is greater for 50% of rPET content than for 0% of rPET content, so crystallites become more differentiated from each other in terms of free volume **(which means that as rPET content increases, it becomes more difficult to stabilize the perfection of crystallites in the SBM process).**

The SBM process itself increases the average crystallite dimensions (note (12)—τ_2_ decreases), which is also confirmed by an increase in the degree of crystallinity of the bottle material during the SBM process (note (14)). While in terms of average values, the increase in rPET content does not change the value of τ_2_. However, for the value of τ_2_, there is a two-factor interaction effect for the SBM process and rPET content. In the SBM process, the τ_2_ decreases for the bottle relative to the preform for both the 0% and 50% rPET content (note (12)), while the decrease in the τ_2_ is greater for 0% rPET content than for 50% rPET content. So, as the rPET content increases, the average crystallite dimensions for the bottle decrease (note (16)), with an increase in the degree of crystallinity measured by DSC (note (26)), so the number of crystallites must increase (**which supports the previously reached conclusion for the preform that rPET content supports the growth of smaller but more numerous and less perfect crystallites, but now also during the SBM process**).

Regardless of the rPET content, in the SBM process, both the M.E. and the IV increase for the bottle material relative to the preform material (note (15)—**PET macromolecules are not being torn apart in the SBM process** because the breaking stress of covalent carbon-carbon bond in a polymer chain is practically 60–100 GPa [29]), but the two-way cross-effects (Table S2) show that the increase in the IV is greater for 0% rPET content than for 50% rPET content (**the increase in the rPET content does not cause a decrease in the IV in the SBM process but inhibits the post-condensation processes occurring in the SBM process**), while the increase in the M.E. is smaller for 0% rPET content than for 50% rPET content (**this is most likely because the crystallites of the material with rPET content are less perfect and, what is more, they are smaller and more numerous, and thus the boundary between the crystalline and amorphous phases increases)**. The opposite correlation of the effect of rPET content on M.E. and IV (note (21)) can be explained by the increased amount of trans conformation in the amorphous phase and, thus, the increase in stiffness of the amorphous phase [30] of the material with a higher rPET content because the increased stiffness of the amorphous phase hinders the migration of free ends of the PET chain (inhibits post-condensation [31]) and hinders the rotation of crystallites in the phase amorphous, which increases the stresses occurring inside the crystallites and at its boundary with the amorphous phase (resulting in increased M.E.).

The τ_3_ time in samples from bottles is shorter than in preforms (note (10)—the average pore size of the free volume is also smaller), while δτ_3_ and σ_3_ are greater for the bottle material than the preform (note (11)). Such an effect can be interpreted as an extension of the free volumes in one direction (the free volumes become more and more ellipsoidal) with a decreasing regularity of the distribution of the average dimensions of the free volumes in the sample volume in the case of materials with an oriented amorphous phase [32]. **Also, as the rPET content increases, τ_3_ decreases (note (20)—the average dimensions of the free volumes decrease, which is consistent with literature data** [33]**)**.

The SBM process (as a whole) increases the value of the σ_3_ (note (11)) and the Δσ_3_ (the variability of the shape of the free volumes increases). The two-factor interaction between the SBM process and rPET content shows that the SBM process for both 0% and 50% rPET content results in an increase in the σ_3_ (note (11)), but the growing trend for the σ_3_ is stronger for 50% rPET content (**with a high rPET content, the SBM process results in greater ellipsoidality of the free volumes than for 0% rPET content, and it is worth emphasizing that the spherical shape, in terms of mechanical strength, is the most structurally efficient and resilient three-dimensional form found in nature (it is the strongest 3D shape)** [34]).

A significant increase in M.E. observed during the SBM process (note (15)) is correlated with crystallization phenomena (notes (14) and (12)) and with the regularity of the distribution of the average dimensions of the crystallites, which increases due to the free volume inside the crystallites (note (13)—δτ_2_ increases). It follows that the change in the shape of the free volume is related also to the free volumes associated with the crystal structures. **Most likely, the decrease in the regularity of crystallite dimensions in terms of free volumes as a result of increasing the rPET content is caused by the increase in the ellipsoidality of these free volumes as the rPET content increases** (the increase in ellipsoidality of free volumes inside crystallites of stretched semi-crystalline polymers was also confirmed in other PALS, SAXS, and WAXS studies [32]). Referring to other studies [32], ellipsoidization of free microvolumes may lead to the occurrence of cavitation in the amorphous phase inside a crystallite subject to rupture deformation.

After the SBM process, the average density of **the amorphous phase for the bottle is lower than the density of the non-oriented amorphous phase, which is explained by the occurrence of M.E., but whose effects are not caused by an increase in the average dimensions of the free volumes present in the preform (because they decrease in the SBM process (note (10)) as a result of the processes of the orientation of the amorphous phase and its crystallization—note (12))—but are caused by an increase in the number of small and ellipsoidal free volumes (note (11))**. What is more, it is unlikely that cavitation occurs in the amorphous phase itself during the SBM process because the amorphous phase of crystalline polymers exhibits amazingly high strength in terms of cavitation stress at the level of 10–20 MPa [35].

The general conclusion from the obtained data shown in Figure 4b,c is that, due to the higher trans conformation amount in the non-oriented amorphous phase, the increase in rPET content enhances crystallite nucleation processes but hinders their growth (and thus crystallites are less perfect), as well as enhances the M.E. occurring in the process SBM, and on the other hand, inhibits the post-condensation processes occurring in the SBM process (note (21)).

In the SBM process, large free volumes occurring in the preform associated with free volumes between unoriented PET chains and in thermally induced crystallites in a very poorly crystalline preform are eliminated in favor of very small free volumes at the boundary between the crystalline and amorphous phase, and inside crystallites induced by deformation in the highly crystalline bottle. However, these small free volumes are still getting longer (their ellipsoidality increases, especially inside the stretched crystallites in the amorphous phase between the lamellas [32]), and even though the average dimensions of free volumes in the preform are much larger than in the bottle, due to the much smaller amount of amorphous phase in the bottle than in the preform (the bottle is much more crystallized), the share of free volumes in relation to the volume of the amorphous phase in the bottle increases, which is manifested by a decrease in the average density of the amorphous phase in relation to the preform material (M.E.). In the SBM process, the average dimensions of the free volumes are very heterogeneously extended (note (11)), but this effect is inversely correlated with the post-condensation phenomenon measured by the IV of the bottle relative to the preform (note (15)).

The above conclusions for the preform and bottle can be explained causally. In the SBM process, an increase in rPET content results in the formation of more numerous but smaller, less perfect, with the greater ellipsoidal shape of free volumes, and less regular crystallites, as well as an increase in microcavitation between the stiffer amorphous phase and the less regular crystalline phase. One fact can explain these phenomena, namely the increased amount of trans conformations in the unoriented amorphous phase with the increase in rPET content (an increase in the stiffness of the non-oriented amorphous phase).

By analyzing the influence of the power of heating lamps and the power of cooling fans to change the linear effect of the rPET content (Figure 1b and Figure 2b) on density, M.E., τ_2_, τ_3_, and δτ_3_, it can be concluded that increasing the power of heating lamps increases the impact of rPET content on the material density while increasing the power of cooling fans reduces the impact of rPET content on the material density (which is mainly related to the crystalline phase). However, increasing the power of heating lamps reduces the impact of rPET content on τ_2_, τ_3_, δτ_3_, and M.E., while increasing the power of cooling fans increases the influence of rPET content on them (which is mainly related to the amorphous phase and crystal dimensions and perfection). Therefore, it can be concluded that the colder the preform, the stronger the impact of the rPET content on changes in bottle amorphous phase microstructure (the colder the preforms, the smaller the tolerance window for the ISBM process parameters, for which repeatable microstructure properties of bottles with the addition of rPET can be obtained). **The above description of the influence of the power of heating lamps and the power of cooling fans on the microstructure of the bottle is widely known in the industry, which confirms the correctness of the adopted methodology for measuring the microstructure.**

### 5.3. Bottle Material

The influence of rPET content, the power of heating lamps, and the power of cooling fans on the τ_3_ are strongly confounded because these parameters affect the process of amorphous phase free volume change (microcavitation), i.e., in the crystalline phase (change of crystalline perfection), in the amorphous phase (orientation of the amorphous phase), and at the crystalline and amorphous phase interface. For the bottle material, both with increasing the rPET content (notes (28) and (29)) and with increasing the power of the heating lamps (notes (44) and (45)), there is a relationship that as the ellipsoidality of the free volumes increases, their average dimensions simultaneously increase.

The ellipsoidality of the free volumes increases significantly in the SBM process (note (11)) but is reduced by increasing the power of the cooling fans (note (60)), especially for high rPET contents (Table S5). In contrast, increasing the power of the heating lamps further increases the ellipsoidality of the free volumes in the SBM process, but only for low cooling fan powers, which also reduces the regularity of distribution of the average dimensions of the free volumes (note (44)). This means that for less heated preforms, the ellipsoidality of the free volumes in the SBM process increases less than for more heated preforms. However, cooling fans, in addition to cooling the preforms, also eliminate the temperature gradient between the inner and outer walls of the preform during its heating in the heating furnace, and this may be the reason that increasing the power of the cooling fans reduces the ellipsoidality of the free volumes in the SBM process, although the confirmation of this postulate requires additional research. The above also shows that the high content of rPET increases the “sensitivity” of microstructure changes to the occurring temperature gradient between the outer and inner surfaces of the preform wall. **The greater the power of cooling fans, the greater the sphericity of free areas, but also the uncertainty of measuring this sphericity increases, i.e., the higher the power of cooling fans (smaller temperature gradient in the preform wall and also cooler preforms** [17]**), the greater the variability of sphericity, with increasing the sphericity of the shape of free areas.**

It is observed that for higher rPET content and higher temperatures of preform, the mechanical strength of the bottle (measured by pressure resistance) decreases (Figure 3). The research shows that with an increase in the rPET content (note (19)), as well as with an increase in the power of heating lamps, but only for low cooling fan powers (note (44)), the increase in ellipsoidality of the free volumes, observed in the SBM process (note (11), increases even more. However, an increase in the power of the cooling fans strongly reduces the impact of the heating lamp power on the ellipsoidality of the free volumes (note (46)), but at the same time, the increase in the power of cooling fans (increased preform cooling) unfortunately increases the impact of the rPET content on increasing the ellipsoidality of the free volumes (note (33)). **The ellipsoidality of the free volumes is strongly related to the mechanical strength, and the studies described in the literature** [32] **show that the small free volumes of the amorphous phase inside the crystallites can increase their ellipsoidality to a certain value, beyond which they merge into large cavitation voids, initiating the phenomenon of sample tearing during uniaxial stretching** [36].

Increasing the rPET content increases the M.E. and the degree of crystallinity (note (26)). Increasing the power of cooling fans increases the τ_3_ (note (56)) and thus increases the mean dimensions of free volumes for bottle material—for high-power cooling fans, the rotation of crystallites in the crystalline matrix is hindered (because due to the lowering of the temperature of the material, the stiffness of the non-crystalline matrix increases). At the same time, the whole SBM process increases the M.E., the degree of crystallinity (note (15) and (14)), and the ellipsoidality of free volumes (note (11)), and decreases the mean dimensions of free volumes for bottle material in relation to the preform material (note (10) in Figure 4b). **This is strong evidence that the increase in the number of small and strongly ellipsoidal free volumes is caused by the rotation of crystalline structures and, thus, the microcavitation phenomena mainly occurs at the border of the crystalline and amorphous phases.**

Although in the SBM process, the effect of microcavitation and post-condensation are correlated (microstructure of the bottle relative to the preform), the analysis of the influence of the rPET content (notes (21) and (27)) and the power of cooling fans (notes (50) and (53)) on the bottle microstructure, shows that the microcavitation and post-condensation effects are inversely correlated for bottle material. It can be concluded that an additional phenomenon affecting both the microcavitation and post-condensation effects occurs in the SBM process. The literature describes research on filling cavitation volumes with a low-molecular compound, which inhibited or even prevented further cavitation processes [35]. It is also known that in the post-condensation process of PET, ethylene glycol (EG) is released [31], and by diffusing into the free volumes, it makes it difficult to change them during the SBM process. So, slowed post-condensation causes a reduction in the release of EG, which in turn slows down the filling of free volumes with this particular EG, and as a consequence, the phenomenon of microcavitation increases, which is especially manifested when the content of rPET is increased (Table S2—the addition of rPET inhibits post-condensation and supports microcavitation), as well as when changing the power of cooling fans, for which the relationship between post-condensation and micocavitation is non-linear and inversely correlated (notes (50) and (53)).

**Figure 5:**
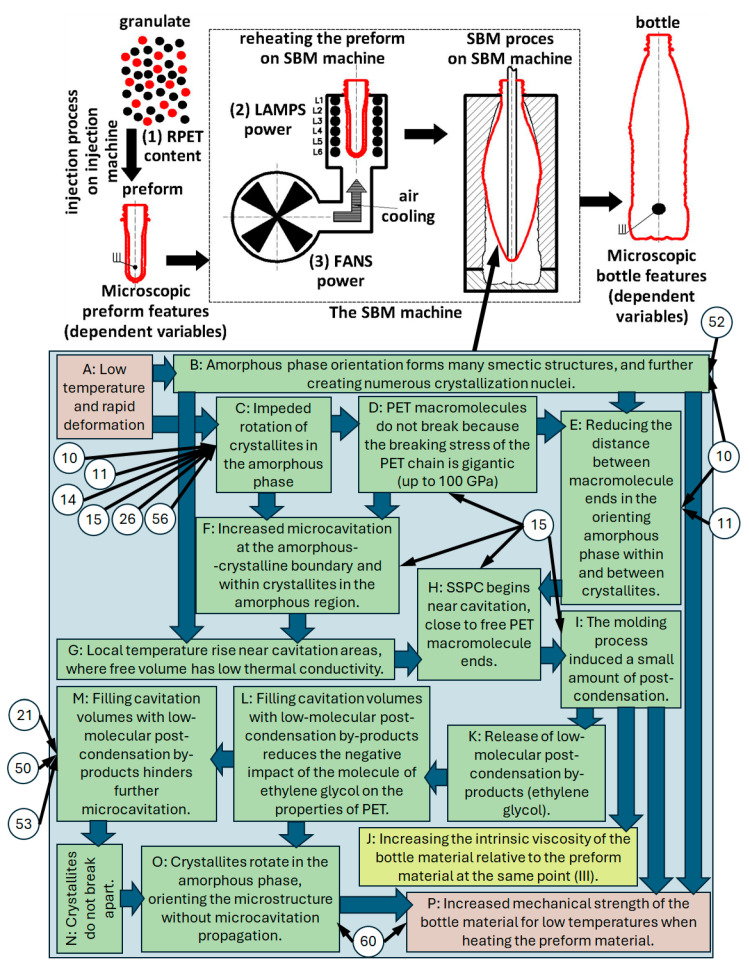
Schematic representation of the interaction potentially occurring in the low-temperature SBM process between the phenomenon of microcavitation and the phenomenon of post-condensation in the solid state and its impact on the microscopic and macroscopic properties of containers produced in the SBM process [18]. The letter designations (A–P) are supported by the literature (description in the literature review work [18]), while the numerical designations (10, 11, 14, 15, 21, 26, 50, 52, 53, 60) correspond to the notes in Figure 4 of the test results (description in the text).

All the above comments, regarding the low-temperature mechanism linking the post-condensation phenomenon with microcavitation through the diffusion of EG from the post-condensation areas into the microcavitation volume, are presented in Figure 5, which is based on the analysis of the literature presented in separate work [18]. The mechanism described in Figure 5 is also explained in detail in terms of literature research [18], but also confirmed by the research described in this part of the work (the markings in the circles shown in Figure 5 refer to the markings in the circles in Figure 4). The phenomena included in Figure 5, described with comments marked with letters A to P, were based on literature research and described in detail on the basis of the literature in the literature review work [18]. The diagram shown in Figure 5 uses the designations 10, 11, 14, 15, 21, 26, 50, 52, 53, and 60, where:

10: In the SBM process, the free volumes decrease, so the distance between PET macromolecules decreases.
11: Additionally, in the SBM process, the ellipsoidality of the free volumes increases, which further reduces the distance between PET macromolecules.
15: In the SBM process, the microcavitation process is correlated with the process of increasing intrinsic viscosity (which is evidence of the occurrence of post-condensation phenomena in the SBM process).
21: When the rPET content increases, the microcavitation process is inversely related to the increase in intrinsic viscosity, which proves that if the post-condensation phenomenon weakens, the microcavitation phenomenon increases.
50 and 53: When the power of the cooling fans is increased, the microcavitation process is inversely related to the increase in intrinsic viscosity, which proves that if the post-condensation phenomenon weakens, the microcavitation phenomenon increases and vice versa.
52: For low-power heat lamps, increasing the fan power increases the glass transition temperature, which is an indicator of the increase in the number of trans conformations in the amorphous phase and, therefore, is an indicator of the orientation of the amorphous phase.
10, 11, 14, 15, 26, 56: The greater the power of the cooling fans, the more difficult it is for the crystallites to rotate in the surrounding amorphous phase (because, due to the lowering of the temperature of the material, the stiffness of the non-crystalline matrix increases).
60: The higher the power of cooling fans (smaller temperature gradient in the preform wall and also cooler preforms [17]), the greater the variability of sphericity, with increasing sphericity of the shape of free areas. The sphere shape in nature, in terms of mechanical strength, is the strongest 3D shape—the reason is that stress is distributed equally along the arc instead of concentrating at any one point. What could be the reason for the increasing mechanical strength of the bottle produced from the less heated preform observed in this study (Figure 3)—the bottle may deform more before the less ellipsoidal free volumes begin to merge and form large cavitation voids [32], causing the process of bottle bursting. In this work, it was proved that microcavitation is correlated not with the increase in the size of free volumes but with the increase in the number and ellipsoidality of very small free volumes.

## 6. Conclusions

The three-factor, three-value tests used in this study are very complex, capital-intensive, and time-consuming, but they enable a holistic view of the impact of the most important factors on the behavior of rPET in the SBM process and, most importantly, such tests enable the examination of the effects of interactions between factors. From the statistically significant effects and based on literature data, many unique conclusions can be drawn.

The research shows that with the increase in rPET content, the number of trans conformations of the ethylene glycol segments of the PET macromolecule in the non-crystalline phase increases. Based on the literature review presented in a separate work [18] (especially [37]), it was concluded that this increase in the amount of trans conformation in the amorphous phase of preforms with the addition of rPET may be a result of the mechanical (material) recycling process of the PET material, and a more effective method of recycling should be developed in terms of removing the “memory” from the PET macromolecule of the history of phase transformations of the material that occurred before recycling. The research described above indirectly shows that, despite the high chemical purity of the PET waste material (which is confirmed by NMR research of preform material containing rPET), in the mechanical (material) recycling process used to obtain PET recyclate added by the preform manufacturer to the preforms used in this study, full information was not removed about the history of phase transformations of PET taking place in previous processes. This raises the question of whether there is any way to remove the excessive amount of trans conformation in the amorphous phase of recycled PET during mechanical (material) recycling with temperatures near the melting temperature.

The amorphous phase of the preform material with rPET content is characterized by an increased content of the trans conformation, as a result of which it crystallizes into smaller, more irregular crystallites during the SBM process because the trans conformation may be the cause of self-nucleation of the material into the crystalline phase; as well, during the growth of the crystallite from the amorphous phase with increased content of the trans conformation, less perfect crystallites are obtained, i.e., with more defects, due to the increased stiffness of the amorphous phase chain containing an increased amount of trans conformation.

The research shows that the increase in the content of rPET promotes the microcavitation process. However, two mechanisms influence the phenomenon of microcavitation in the amorphous phase. The first one is the preform heating process (the lower the temperature of the preform, the stronger the microcavitation phenomenon during deformation), and the second one is the amount of trans conformations in the amorphous phase before the start of the deformation process in the SBM process (the larger the number of trans conformations in the amorphous phase before the start of deformation, the stronger microcavitation phenomenon during deformation).

The higher the rPET content and the more strongly the preform is heated in the heating oven, the more ellipsoidal shapes of the free volumes in the bottle material, which could be the reason for the lowering of the mechanical strength of the bottle produced from more heated preform observed in pressure resistance tests (the bottle may deform less before the more ellipsoidal free volumes begin to merge and form large cavitation voids [32] causing the process of bottle bursting). Further research will be continued in this area.

Comparing the microstructure of the bottle to the microstructure of the preform, in the SBM process, the microcavitation effect is correlated with the post-condensation effect. However, when changing the rPET content and cooling fan power, the microcavitation effect and the intrinsic viscosity are inversely correlated within the bottle material. It can be concluded that an additional phenomenon affecting both the microcavitation and post-condensation effects occurs in the SBM process. The literature review presented in a separate work [18] describes research on filling cavitation volumes with a low-molecular compound, which inhibited or even prevented further cavitation processes. However, further research must be conducted in this area.

## Data Availability

All data, including equations used in statistical analysis, have been included in the Supporting Information in first part of the article [17]. If necessary, data will be sent upon request. Also, a full interpretation of Figure 4 will sent upon request.

## Supplementary Materials

The following supporting information can be downloaded at: https://www.mdpi.com/article/10.3390/ma18010036/s1, Table S1: Symbolic interpretation of the linear and quadratic influence of rPET content on the microscopic features of the preform material shown in Figure 1a and Figure 2a; Table S2: Symbolic interpretation of the linear effect of the SBM process (A) and rPET content (B) on the microscopic features of the bottle relative to the preform shown in Figure 1b and Figure 2b—symbolic interpretation of linear two-way interactions from the graph presented in Figure 1b and Figure 2b necessary for the interpretation of the linear main effects presented in Figure 1b and Figure 2b was based on the Table S2 describing the interpretation of the two-factor cross-effects presented in the first part of the article (part I [17]), where (a) is mean, (b) is fitting uncertainty, (c) is standard uncertainty; Table S3: Symbolic interpretation of the quadratic influence of the SBM process (A) and rPET content (B) on the microscopic features of the bottle in relation to the preform shown in Figure 1b and Figure 2b—symbolic interpretation of the quadratic effects in relation to the linear effects was based on the idea presented in Figure S1 describing the interpretation of quadratic effects versus linear effects presented in the first part of the article (part I [17]), where: (a) is mean, (b) is fitting uncertainty, (c) is standard uncertainty; Table S4: Symbolic interpretation of the linear two-way interactions from the graph presented in Figure 1c, Figure 2c and Figure 3 of the influence of rPET content (A), the power of heating lamps (B), and the power of cooling fans (C) on the microscopic features of the bottle, necessary to interpret the linear main effects presented in Table S5 based on the Table S2 describing the interpretation of the two-factor cross-effects presented in the first part of the article (part I [17]), where (a) is mean, (b) is fitting uncertainty, (c) is standard un-certainty; Table S5: Symbolic interpretation of the linear main effects from the graphs presented in Figure 1c, Figure 2c and Figure 3 in relation to the linear two-way effects presented in Table S4 and the Table S2 describing the interpretation of the two-way cross effects of the influence of rPET (A) content, the power of heating lamps (B), and the power of cooling fans (C) on the microscopic features of the bottle, where (a) is mean, (b) is fitting uncertainty, (c) is standard uncertainty; Table S6: Symbolic interpretation of the quadratic main effects from the graphs shown in Figure 1c, Figure 2c and Figure 3 of the influence of rPET content (A), the power of heating lamps (B), and the power of cooling fans (C) on the microscopic features of the bottle in relation to the linear main effects presented in Tables S4 and S5 based on the idea presented in Figures S1 and S2 describing the interpretation of quadratic effects in relation to the linear effects presented in the first part of the article [17], where (a) is mean, (b) is fitting uncertainty, (c) is standard uncertainty; Table S7: The calculated standardized effects (s.effect), *p*-values, power, and adjusted R^2 (aR^2) values for every experimental design (Table A1). The colors are explained in Tables S8 and S9; Table S8: Explanation of four colors for the analysis of the *p*-value (statistical significance of a given effect in the analyzed model) versus the power of the ANOVA test (the higher the power of the test, the greater the probability that a given statistically insignificant effect is actually statistically insignificant) used in Table S7; Table S9. Explanation of three colors for the analysis of the value of the R^2 parameter used in Table S7.

## Appendix A

**Table A1:** Test plans, independent and dependent variables, run order, and the number of samples for the measurement series, testing the microscopic and macroscopic features of bottles, preforms, and bottles relative to preforms (the SBM process); graphic representations of the test plans are shown in Figure A1 [17].

Measurement Series						Run order of Bottle Measurement Series ^6^	Number of Bottles	Independent Variables										Dependent Variables ***					
Bottle ^a^	Preform ^b^	SBM Process (Bottle vs. Preform)						RPET Content			Power of LAMPS			Power of FANS			FORM	Microscopic				Macroscopic	
		“ALL” ^c^	“RPET” ^d^	“RPET+ LAMPS” ^e,7^	“RPET + FANS” ^f,7^			(−1)	(0)	(1)	(−1)	(0)	(1)	(−1)	(0)	(1)		ρ ^1,2^	DSC ^1,2^	PALS ^1^	IV ^1^	TH ^3^ (I,II,III)	PRT ^4^
								0%	25%	50%	−10%	0%	10%	−5%	0%	5%		Number of Samples					
A1	N/A	B4	N/A	N/A	N/A	15	15	(−1)			(−1)			(−1)			Bottle **	1	1	1	1	5 × 3	10
A2	N/A		N/A	N/A	N/A	4	15	(−1)			(−1)			(1)				1	1	1	1	5 × 3	10
A3	N/A		B7	B7	B7	8	15	(−1)			(0)			(0)				1	1	1	1	5 × 3	10
A4	N/A		N/A	N/A	N/A	1	15	(−1)			(1)			(−1)				1	1	1	1	5 × 3	10
A5	N/A		N/A	N/A	N/A	14	15	(−1)			(1)			(1)				1	1	1	1	5 × 3	10
A6	N/A	B5	N/A	B10	N/A	13	15	(0)			(−1)			(0)				1	1	1	1	5 × 3	10
A7	N/A		N/A	N/A	B11	6	15	(0)			(0)			(−1)				1	1	1	1	5 × 3	10
A8	N/A		B8	B10		11	15	(0)			(0)			(0)				3 ^5^	3 ^5^	3 ^5^	3 ^5^	5 × 3	10
A9	N/A		N/A	N/A		10	15	(0)			(0)			(1)				1	1	1	1	5 × 3	10
A10	N/A		N/A	B10	N/A	3	15	(0)			(1)			(0)				1	1	1	1	5 × 3	10
A11	N/A	B6	N/A	N/A	N/A	12	15	(1)			(−1)			(−1)				1	1	1	1	5 × 3	10
A12	N/A		N/A	N/A	N/A	2	15	(1)			(−1)			(1)				1	1	1	1	5 × 3	10
A13	N/A		B9	B9	B9	9	15	(1)			(0)			(0)				1	1	1	1	5 × 3	10
A14	N/A		N/A	N/A	N/A	7	15	(1)			(1)			(−1)				1	1	1	1	5 × 3	10
A15	N/A		N/A	N/A	N/A	5	15	(1)			(1)			(1)				1	1	1	1	5 × 3	10
N/A	p0.0	B1				-		(−1)			-			-			Preform *	(−1)	-	-	1	-	-
N/A	p0.25	B2				-		(0)			-			-				(0)	-	-	3	-	-
N/A	p0.5	B3				-		(1)			-			-				(1)	-	-	1	-	-

^1,2,3,4,5,6,7,^*^,^**^,^*** A detailed description of the table is presented in the previous paper in Table 1 [17]. ^a–f^ A graphic representation of the test plans is shown in Figure A1. N/A means “not applicable”.

Table A2 and Table A3 show the test plan for the measurement series marked with the letter A (Table A1a) and marked with the letter B (Table A1b, c, and d), respectively.

**Table A2:** Experimental design for design (a), which is shown in Table A1 and Figure A1, using series designations with the letter “A” (the plan is described in detail in [17]).

NoE	Main Factors			Interaction Factors			Mean Response
	(1) RPET Content	(2) Power of LAMPS	(3) Power of FANS	(1) × (2)	(1) × (3)	(2) × (3)	$\bar{y_{Ai}}$
A1	−1	−1	−1	1	1	1	$\bar{y_{1}}$
A2	−1	−1	1	1	−1	−1	$\bar{y_{2}}$
A3	−1	0	0	0	0	0	$\bar{y_{3}}$
A4	−1	1	−1	−1	1	−1	$\bar{y_{4}}$
A5	−1	1	1	−1	−1	1	$\bar{y_{5}}$
A6	0	−1	0	0	0	0	$\bar{y_{6}}$
A7	0	0	−1	0	0	0	$\bar{y_{7}}$
A8	0	0	0	0	0	0	$\bar{y_{8}}$
A9	0	0	1	0	0	0	$\bar{y_{9}}$
A10	0	1	0	0	0	0	$\bar{y_{10}}$
A11	1	−1	−1	−1	−1	1	$\bar{y_{11}}$
A12	1	−1	1	−1	1	−1	$\bar{y_{12}}$
A13	1	0	0	0	0	0	$\bar{y_{13}}$
A14	1	1	−1	1	−1	−1	$\bar{y_{14}}$
A15	1	1	1	1	1	1	$\bar{y_{15}}$

**Table A3:** Experimental designs for designs (c) and (d), which are shown in Table A1 and Figure A1, using series designations with the letter “B”.

NoE	Main Factors		Interaction Factors	Mean Response
	(1) FORM (SBM)	(2) RPET Content	(1) × (2)	$\bar{y_{Bi}}$
B1 (p0.0)	−1	−1	1	$\bar{y_{B1}}$
B2 (p0.25)	−1	0	0	$\bar{y_{B2}}$
B3 (p0.5)	−1	1	−1	$\bar{y_{B3}}$
B4	1	−1	−1	$\bar{y_{B4}}$
B5	1	0	0	$\bar{y_{B5}}$
B6	1	1	1	$\bar{y_{B6}}$
B7	1	−1	−1	$\bar{y_{B7}}$
B8	1	0	0	$\bar{y_{B8}}$
B9	1	1	1	$\bar{y_{B9}}$

## Appendix B

### Appendix B.1. The Method of Calculating the Effect Value for Plans (a)–(d) (Table A1) Based on the Example of Density Measurements

Table A1a shows the CCF three-way, trivalent, incomplete design with 15 measurement series for the bottle (A1–A15), and Table A1b shows the one-factor three-valued design for the preform (p0.0, p0.25, p0.5). The results of measurements of some examples of data research on the density, crystallinity (measured in DSC), and τ_3_ lifetime (measured in PALS) of the preform and bottle material are summarized in Table A4 (see Table S4 in [17]). Since, through the CCF plan methodology, the standard deviation was assumed to be equal to the measured standard deviation for the A8 series for all measurement series for the bottle and equal to the measured standard deviation for the p0.25 series for all measurement series for the preform, the homogeneity of variance between series tested by Levene’s test [15] showed that all measurement series shown in Table A4 are homogeneous in terms of variance.

**Table A4:** Presentation of some examples of measurement data research results on the density, crystallinity (measured in DSC), and τ_3_ lifetime (measured in PALS) of the bottles and preforms material, where measurement series are related to Table A1 (see Tables S4–S7 in [17]).

Examples of Measurement Data (Tables S4–S7 [17])		Responses for Bottles (“A” Series)															Responses for Preforms		
		A1	A2	A3	A4	A5	A6	A7	A8	A9	A10	A11	A12	A13	A14	A15	p0.0	p0.25	p0.5
Density [g/cm³]	Mean ($\bar{y_{i}}$)	1.3646	1.3635	1.3648	1.3649	1.3650	1.3644	1.3651	1.3647	1.3646	1.3654	1.3635	1.3629	1.3648	1.3651	1.3655	1.3409	1.3427	1.3492
	Measurement uncertainty	-	-	-	-	-	-	-	0.0002	-	-	-	-	-	-	-	-	0.0021	-
	Sample size	1	1	1	1	1	1	1	3	1	1	1	1	1	1	1	1	3	1
Crystallinity [%] (measured in DSC)	Mean ($\bar{y_{i}}$)	31.3	31.2	29.7	32.6	32.2	31.5	31.9	30.7	31.0	30.3	31.8	29.8	32.3	31.0	33.2	3.4	4.4	5.4
	Measurement uncertainty	-	-	-	-	-	-	-	1.2	-	-	-	-	-	-	-	-	0.8	-
	Sample size	1	1	1	1	1	1	1	3	1	1	1	1	1	1	1	1	3	1
τ_3_ lifetime [ns] (measured in PALS)	Mean ($\bar{y_{i}}$)	1.4810	1.5430	1.5250	1.4830	1.5230	1.4670	1.4890	1.5053	1.5210	1.4915	1.4970	1.4890	1.5080	1.4830	1.4870	1.5240	1.5267	1.5160
	Measurement uncertainty	-	-	-	-	-	-	-	0.0237	-	-	-	-	-	-	-	-	0.0043	-
	Sample size	1	1	1	1	1	1	1	3	1	1	1	1	1	1	1	1	3	1

All further examples of calculations necessary to construct the graphs of Figure 1, Figure 2 and Figure 3 will be presented using density measurements as an example. Also, an example of the interpretation of the DOE analyses presented in Figure 1, Figure 2 and Figure 3 will be presented using density measurements as an example (Appendix C). All calculations and interpretations for the remaining dependent variables were performed in an analogous manner.

Table A5 shows the method of calculating the main linear effect (τ) and main quadratic effect (τ^2), with Formulas (A1) and (A2), respectively, in the one-factor three-valued design for the preform (Table A1b) for density measurements (Table A4). Table A6 shows the results of calculations in the Statistica 13 environment of the effect value (see Table A5), standard error (SE), standardized effect (understood as the ratio of the effect value to the standard error), the *p*-value in ANOVA (see Table A15), statistically significant standardized effect (values of statistically insignificant effects were given as zero), and the model fit parameter which is the adjusted R^2.

**Table A5:** The method of calculating the main linear effect (τ) and main quadratic effect (τ^2) with the names of the effects used in Figure 1a and Figure 2a.

Effects in Figure 1a and Figure 2a—see Table A1 and Figure A1 (plan (b))	
$\tau \equiv \left(1\right)RPETcontent\left(L\right)=\bar{y_{B3}}-\bar{y_{B1}}=0.0083$	(A1)
τ^2≡1RPETcontentQ=yB2¯−12yB3¯+yB1¯=−0.0024	(A2)

**Table A6:** The results of calculations in the Statistica 13 environment of the effect value (see Table A5), standard error (SE), standardized effect (understood as the ratio of the effect value to the standard error), the *p*-value in ANOVA (see Table A15), statistically significant standardized effect (values of statistically insignificant effects were given as zero), and the model fit parameter which is the adjusted R^2 with the names of the effects used in Figure 1a and Figure 2a.

Name	Model Fit: R^2 = 0.82. The Model Is Acceptably Fitted for R^2 > 0.8.				
	Effect	Standard Error (SE)	Standardized Effect = Effect/SE	$p\;(p_{cr}=0.05$) (See Table A12)	Significant Standardized Effect
Global mean	1.3427	0.001193	1125.5	0.000	1125.454
(1) RPET content (L)	0.0083	0.001687	4.9	0.003	4.919
(1) RPET content (Q)	−0.0024	0.001461	−1.6	0.134	0

Table A7 shows the method of calculating the main linear effects (τ, β), main quadratic effect (β^2), and linear two-way interaction effect ((τβ)), with Formulas (A3), (A4), (A5), and (A6), respectively, in the two-factor study of the impact of the SBM process (bivalent factor) and rPET content (three-valued factor) on the “relative” properties of the bottles in relation to the properties of the preforms (for each rPET content value, the lamp power and fan power settings were not fixed and changed (“ALL” plan)—see Table A1c) for density measurements (Table A4). Table A8 shows the results of calculations in the Statistica 13 environment of the effect values (see Table A7), standard error (SE), standardized effect (understood as the ratio of the effect value to the standard error), the *p*-value in ANOVA (see Table A16), statistically significant standardized effect (values of statistically insignificant effects were given as zero), and the model fit parameter which is the adjusted R^2.

**Table A7:** The method of calculating the main linear effects (τ, β), main quadratic effect (β^2), and linear two-way interaction effect ((τβ)) with the names of the effects used in Figure 1b and Figure 2b for “ALL” plan.

Effects in Figure 1b and Figure 2b—see Table A1 and Table A2, and Figure A1 (plan (c))	
$\tau \equiv \left(1\right)FORM\left(L\right)=\frac{\bar{y_{B4}}+\bar{y_{B5}}+\bar{y_{B6}}}{3}-\frac{\bar{y_{B1}}+\bar{y_{B2}}+\bar{y_{B3}}}{3}=0.02032$	(A3)
$\beta \equiv \left(2\right)RPET\;content\left(L\right)=\frac{\bar{y_{B3}}+\bar{y_{B6}}}{2}-\frac{\bar{y_{B1}}+\bar{y_{B4}}}{2}=0.00405$	(A4)
β^2≡2RPET contentQ=yB2¯+yB5¯2−12yB3¯+yB6¯2+yB1¯+yB4¯2=−0.00099	(A5)
$\left(\tau \beta \right)\equiv 1L\;vs.\;2L=\frac{\bar{y_{B1}}+\bar{y_{B6}}}{2}-\frac{\bar{y_{B3}}+\bar{y_{B4}}}{2}=-0.00425$	(A6)

**Table A8:** The results of calculations in the Statistica 13 environment of the effect value (see Table A7), standard error (SE), standardized effect (understood as the ratio of the effect value to the standard error), the *p*-value in ANOVA (see Table A16), statistically significant standardized effect (values of statistically insignificant effects were given as zero), and the model fit parameter which is the adjusted R^2 with the names of the effects used in Figure 1b and Figure 2b for “ALL” plan.

Name	Model Fit: Adjusted R^2 = 0.98. The Model Is Acceptably Fitted for R^2 > 0.8.				
	Effect	Standard Error (SE)	Standardized Effect = Effect/SE	$p\;(p_{cr}=0.05$) (See Table A12)	Significant Standardized Effect
Global mean	1.3544	0.000203	6682.1	0.000	6682.1
(1) FORM (L)	0.02032	0.000405	47.9	0.000	47.9
(2) RPET content (L)	0.00405	0.000496	8.2	0.000	8.2
(2) RPET content (Q)	−0.00099	0.000320	−3.1	0.002	−3.1
1L vs. 2L	−0.00425	0.000496	−8.6	0.000	−8.6

Table A9 shows the method of calculating the main linear effects (τ, β), main quadratic effect (β^2), and linear two-way interaction effect ((τβ)), with Formulas (A7), (A8), (A9), and (A10), respectively, in the two-factor study of the impact of the SBM process (bivalent factor) and rPET content (three-valued factor) on the “relative” properties of the bottles in relation to the properties of the preforms (for individual rPET content, the lamp power and fan power settings were fixed on the central values from the plan shown in Table A1 as A8 series (“RPET” plan)—see Table A1d) for density measurements (Table A4). Table A10 shows the results of calculations in the Statistica 13 environment of the effect values (see Table A9), standard error (SE), standardized effect (understood as the ratio of the effect value to the standard error), the *p*-value in ANOVA (see Table A16), statistically significant standardized effect (values of statistically insignificant effects were given as zero), and the model fit parameter which is the adjusted R^2.

**Table A9:** The method of calculating the main linear effects (τ, β), main quadratic effect (β^2), and linear two-way interaction effect ((τβ)) with the names of the effects used in Figure 1b and Figure 2b for “RPET” plan.

Effects in Figure 1b and Figure 2b—see Table A1 and Table A2, and Figure A1 (plan (d))	
$\tau \equiv \left(1\right)FORM\left(L\right)=\frac{\bar{y_{B7}}+\bar{y_{B8}}+\bar{y_{B9}}}{3}-\frac{\bar{y_{B1}}+\bar{y_{B2}}+\bar{y_{B3}}}{3}=0.0205$	(A7)
$\beta \equiv \left(2\right)RPET\;content\left(L\right)=\frac{\bar{y_{B3}}+\bar{y_{B9}}}{2}-\frac{\bar{y_{B1}}+\bar{y_{B7}}}{2}=0.0042$	(A8)
β^2≡2RPET contentQ=yB2¯+yB8¯2−12yB3¯+yB9¯2+yB1¯+yB7¯2=−0.0012	(A9)
$\left(\tau \beta \right)\equiv 1L\;vs.\;2L=\frac{\bar{y_{B1}}+\bar{y_{B9}}}{2}-\frac{\bar{y_{B3}}+\bar{y_{B7}}}{2}=-0.0042$	(A10)

**Table A10:** The results of calculations in the Statistica 13 environment of the effect value (see Table A9), standard error (SE), standardized effect (understood as the ratio of the effect value to the standard error), the *p*-value in ANOVA (see Table A17), statistically significant standardized effect (values of statistically insignificant effects were given as zero), and the model fit parameter which is the adjusted R^2 with the names of the effects used in Figure 1b and Figure 2b for “RPET” plan.

Name	Model Fit: Adjusted R^2 = 0.99. The Model Is Acceptably Fitted for R^2 > 0.8				
	Effect	Standard Error (SE)	Standardized Effect = Effect/SE	$p\;(p_{cr}=0.05$) (See Table A12)	Significant Standardized Effect
Global mean	1.3545	0.000365	3712.4	0.000	3712.4
(1) FORM (L)	0.0205	0.000730	27.1	0.000	27.1
(2) RPET content (L)	0.0042	0.000894	4.6	0.002	4.6
(2) RPET content (Q)	−0.0012	0.000774	−1.6	0.117	0
1L vs. 2L	−0.0042	0.000894	−4.6	0.000	−4.6

Table A11 shows the method of calculating the main linear effects (τ, β, γ), main quadratic effects (τ^2, β^2, γ^2), and linear two-way interaction effects ((τβ), (τγ), (βγ)), with Formulas (S9), (S10), (S11), (S12), (S13), (S14), (S15), (S16), and (S17), respectively, in the of the CCF experiment (Table A1a) for density measurements (Table A4). Table A12 shows the results of calculations in the Statistica 13 environment of the effect values (see Table A11), standard error (SE), standardized effect (understood as the ratio of the effect value to the standard error), the *p*-value in ANOVA (see Table A18), statistically significant standardized effect (values of statistically insignificant effects were given as zero), and the model fit parameter which is the adjusted R^2.

**Table A11:** The method of calculating the main linear effects (τ, β, γ), main quadratic effects (τ^2, β^2, γ^2), and linear two-way interaction effects ((τβ), (τγ), (βγ)) with the names of the effects used in Figure 1c, Figure 2c and Figure 3 (the equations are described in detail in the Supporting Information in [17]).

Effects in Figure 1c, Figure 2c and Figure 3—see Table A1, Figure A1 (plan (a)), and Table S1 [17]	
$\tau \equiv \left(1\right)RPET\;content\left(L\right)=\frac{\bar{y_{A11}}+\bar{y_{A12}}+\bar{y_{A13}}+\bar{y_{A14}}+\bar{y_{A15}}}{5}-\frac{\bar{y_{A1}}+\bar{y_{A2}}+\bar{y_{A3}}+\bar{y_{A4}}+\bar{y_{A5}}}{5}=-0.0002$	(A11)
$\beta \equiv \left(2\right)power\;of\;heating\;lamps\left(L\right)=\frac{\bar{y_{A4}}+\bar{y_{A5}}+\bar{y_{A10}}+\bar{y_{A14}}+\bar{y_{A15}}}{5}-\frac{\bar{y_{A1}}+\bar{y_{A2}}+\bar{y_{A6}}+\bar{y_{A11}}+\bar{y_{A12}}}{5}=0.0014$	(A12)
$\gamma \equiv \left(3\right)power\;of\;cooling\;fans\left(L\right)=\frac{\bar{y_{A2}}+\bar{y_{A5}}+\bar{y_{A9}}+\bar{y_{A12}}+\bar{y_{A15}}}{5}-\frac{\bar{y_{A1}}+\bar{y_{A4}}+\bar{y_{A7}}+\bar{y_{A11}}+\bar{y_{A14}}}{5}=-0.0003$	(A13)
τ^2≡1RPET contentQ==yA6¯+yA7¯+yA8¯+yA9¯+yA10¯5−12yA11¯+yA12¯+yA13¯+yA14¯+yA15¯5+yA1¯+yA2¯+yA3¯+yA4¯+yA5¯5=0.0004	(A14)
β^2≡2power of heating lampsQ==yA3¯+yA7¯+yA8¯+yA9¯+yA13¯5−12yA4¯+yA5¯+yA10¯+yA14¯+yA15¯5+yA1¯+yA2¯+yA6¯+yA11¯+yA12¯5=0.0003	(A15)
γ^2≡3power of cooling fansQ==yA3¯+yA6¯+yA8¯+yA10¯+yA13¯5−12yA2¯+yA5¯+yA9¯+yA12¯+yA15¯5+yA1¯+yA4¯+yA7¯+yA11¯+yA14¯5=0.0004	(A16)
$\left(\tau \beta \right)\equiv 1L\;vs.\;2L=\frac{\bar{y_{A1}}+\bar{y_{A2}}+\bar{y_{A14}}+\bar{y_{A15}}}{4}-\frac{\bar{y_{A4}}+\bar{y_{A5}}+\bar{y_{A11}}+\bar{y_{A12}}}{4}=0.0006$	(A17)
$\left(\tau \gamma \right)\equiv 1L\;vs.\;3L=\frac{\bar{y_{A1}}+\bar{y_{A4}}+\bar{y_{A12}}+\bar{y_{A15}}}{4}-\frac{\bar{y_{A2}}+\bar{y_{A5}}+\bar{y_{A11}}+\bar{y_{A14}}}{4}=0.0002$	(A18)
$\left(\beta \gamma \right)\equiv 2L\;vs.\;3L=\frac{\bar{y_{A1}}+\bar{y_{A5}}+\bar{y_{A11}}+\bar{y_{A15}}}{4}-\frac{\bar{y_{A2}}+\bar{y_{A4}}+\bar{y_{A12}}+\bar{y_{A14}}}{4}=0.0006$	(A19)

**Table A12:** The results of calculations in the Statistica 13 environment of the effect value (see Table A11), standard error (SE), standardized effect (understood as the ratio of the effect value to the standard error), the *p*-value in ANOVA (see Table A18), statistically significant standardized effect (values of statistically insignificant effects were given as zero), and the model fit parameter which is the adjusted R^2 with the names of the effects used in Figure 1c, Figure 2c and Figure 3 (the method of calculation is described in detail in the Supporting Information in [17]).

Name	Model Fit: Adjusted R^2 = 0.92. The Model Is Acceptably Fitted for R^2 > 0.8.				
	Effect	Standard Error (SE)	Standardized Effect = Effect/SE	$p\;(p_{cr}=0.05$) (See Table A12)	Significant Standardized Effect
Global mean	1.3646	0.000031	43,781.6	0.000	43,781.6
(1) RPET content (L)	−0.0002	0.000076	−2.6	0.013	−2.6
(1) RPET content(Q)	0.0004	0.000075	5.1	0.000	5.1
(2) power of heating lamps (L)	0.0014	0.000076	18.3	0.000	18.3
(2) power of heating lamps (Q)	0.0003	0.000075	4.3	0.000	4.3
(3) power of cooling fans (L)	−0.0003	0.000076	−4.5	0.000	−4.5
(3) power of cooling fans (Q)	0.0004	0.000075	4.7	0.000	4.7
1L vs. 2L	0.0006	0.000085	7.0	0.000	7.0
1L vs. 3L	0.0002	0.000085	2.3	0.025	2.3
2L vs. 3L	0.0006	0.000085	6.4	0.000	6.4

### Appendix B.2. The Method of Calculating the Statistical Significance of the Effect by ANOVA and the Power of ANOVA for Plans (a)–(d) (Table A1) Based on the Example of Density Measurements

The statistical analysis should check whether the constructed model significantly reflects the influence of the independent variables on the dependent variables—for this, an ANOVA is required. Table S3 [17] summarizes the formulas needed to perform ANOVA for an incomplete three-valued, three-factor design shown in Table A1a.

All ANOVA tests were conducted for an arbitrarily assumed 5% probability of making a type I error ($\alpha =p_{cr}=0.05$) [17]. All calculations for the power of the ANOVA test were conducted for the assumption that the minimum power cannot be lower than 80%, i.e., the probability of making a type II error cannot exceed 20% ($\beta_{cr}=0.2$) [17]. Knowledge of the value of the probability of making a type II error (β) makes it possible to calculate the test power according to Formula (S98) [17]. The steps for calculating the test power for each effect are summarized in Figure S4 [17].

Table A13, Table A14, Table A15 and Table A16 show the results of calculations in the Statistica 13 environment of the sum of squares (SS), degree of freedom (df), the mean sum of squares (MS), F statistic value (F), *p*-value in the ANOVA, Fcr as F statistic value for critical *p*-value ($\alpha =p_{cr}=0.05$) in ANOVA, δ as the non-central parameter for the non-central probability density function of F statistic, β as the probability of making a type II error ($\beta_{cr}=0.2$) for non-central probability density function of F statistic [15], and the power of ANOVA (the methods of calculations are described in detail in Table S3 [17] and in Figure S4 [17]). The results shown in Table A13, Table A14, Table A15 and Table A16 refer to Table A6, Table A8, Table A10 and Table A12, respectively. Table A13 also shows an example of how to calculate *p*-values in ANOVA (Equations (A20) and (A21)) based on Equations (S19)–(S91) [17] (all *p*-values in ANOVA presented in Table A14, Table A15 and Table A16 can be calculated analogously based on Table S3 [17]).

**Table A13:** The results of calculations in the Statistica 13 environment of the *p*-value in the ANOVA and the power of the test of the ANOVA for the effect value (see Table A6) with the names of the effects used in Figure 1a and Figure 2a.

Name	Symbol [17]	*p*-Value in the ANOVA					Power of ANOVA			
		SS	df	MS	F	p	Fcr	δ	β	Power
(1) RPET content (L)	A	0.000103	1	0.0001	24.193939	0.0026602	5.9873776	13.24	0.14	0.86
(1) RPET content (Q)	A^2^	0.000013	1	0.00001	2.992342	0.1343849	5.9873776	1.64	0.8083123	0.19
Error		0.000026	6	0.000004						
Total		0.000142	8							
where: $\tilde{y}=\frac{\bar{y_{B1}}+\bar{y_{B2}}+\bar{y_{B3}}}{3}=1.3443$										
$df_{total}=\sum_{i=1}^{3}N_{i}-1=\left(3+3+3\right)-1=8$										(S19) [17]
$df_{error}=df_{total}-\left(df_{A}+df_{A^{2}}\right)=8-\left(1+1\right)=6$										(S20) [17]
Positive mean and positive group size for the linear (A) main effect ${\bar{y}}_{+A}=\bar{y_{B3}}=1.3492;N_{+A}=N_{B3}=3$										(S21) [17]
Negative mean and negative group size for the linear (A) main effect ${\bar{y}}_{-A}=\bar{y_{B1}}=1.3409;N_{-A}=N_{B1}=3$										(S22) [17]
Mean for the linear main effect of the Factor (A) ${\bar{y}}_{A}=\frac{{\bar{y}}_{+A}+{\bar{y}}_{-A}}{2}=1.3451$										(S23) [17]
Positive mean and positive group size for the quadratic (A^2^) main effect ${\bar{y}}_{+A^{2}}=\bar{y_{B2}}=1.3427;N_{+A^{2}}=N_{B2}=3$										(S30) [17]
Negative mean and negative group size for the quadratic RPET (A^2^) main effect $\begin{array}{c} {\bar{y}}_{-A^{2}}=\frac{1}{2}\left(\bar{y_{B3}}+\bar{y_{B1}}\right)=1.3451\\ N_{-A^{2}}=N_{B1}+N_{B3}=6 \end{array}$										(S31) [17]
Mean for the quadratic main effect of the Factor (A^2^) ${\bar{y}}_{A^{2}}=\frac{{\bar{y}}_{+A^{2}}+{\bar{y}}_{-A^{2}}}{2}=1.3439$										(S32) [17]
$\begin{array}{c} SS_{A.ef}={\left({\bar{y}}_{+A}-{\bar{y}}_{A}\right)}^{2}\cdot N_{+A}+{\left({\bar{y}}_{-A}-{\bar{y}}_{A}\right)}^{2}\cdot N_{-A}=\\ ={\left(1.3492-1.3451\right)}^{2}\cdot 3+{\left(1.3409-1.3451\right)}^{2}\cdot 3=1.03\cdot 10^{-4} \end{array}$										(S67) [17]
$\begin{array}{c} {SS}_{A^{2}.ef}={\left({\bar{y}}_{+A^{2}}-{\bar{y}}_{A^{2}}\right)}^{2}\cdot N_{+A^{2}}+{\left({\bar{y}}_{-A^{2}}-{\bar{y}}_{A^{2}}\right)}^{2}\cdot N_{-A^{2}}=\\ ={\left(1.3427-1.3439\right)}^{2}\cdot 3+{\left(1.3451-1.3439\right)}^{2}\cdot 6=1.3\cdot 10^{-5} \end{array}$										(S70) [17]
$\begin{array}{c} \begin{array}{c} {SS}_{total}=SS_{A.ef}+SS_{A^{2}.ef}+SS_{error}=\sum_{i=1}^{N_{j}=3}\sum_{j=-1}^{1}{\left(y_{ij}-\tilde{y}\right)}^{2}=\\ ={\left(y_{1B1}-\tilde{y}\right)}^{2}+{\left(y_{2B1}-\tilde{y}\right)}^{2}+{\left(y_{3B1}-\tilde{y}\right)}^{2}+{\left(y_{1B2}-\tilde{y}\right)}^{2}+{\left(y_{2B2}-\tilde{y}\right)}^{2}+{\left(y_{3B2}-\tilde{y}\right)}^{2}+ \end{array}\\ +{\left(y_{1B3}-\tilde{y}\right)}^{2}+{\left(y_{2B3}-\tilde{y}\right)}^{2}+{\left(y_{3B3}-\tilde{y}\right)}^{2}=1.42\cdot 10^{-4} \end{array}$										(S77) [17]
$SS_{error}=\sum_{i=-1}^{1}\sum_{j=1}^{N_{i}}{\left(y_{ij}-{\bar{y}}_{i•}\right)}^{2}={SS}_{total}-\left(SS_{A.ef}+SS_{A^{2}.ef}\right)=2.6\cdot 10^{-5}$										(S76) [17]
$MS_{A.ef}={\left({\hat{s}}_{1.A}\right)}^{2}=\frac{SS_{A.ef}}{df_{A}}=\frac{1.03\cdot 10^{-4}}{1}=1.03\cdot 10^{-4}$										(S78) [17]
$MS_{A^{2}.ef}={\left({\hat{s}}_{1.A^{2}}\right)}^{2}=\frac{SS_{A^{2}.ef}}{df_{A^{2}}}=\frac{1.3\cdot 10^{-5}}{1}=1.3\cdot 10^{-5}$										(S81) [17]
$MS_{error}={\left({\hat{s}}_{2}\right)}^{2}=\frac{SS_{error}}{df_{error}}=\frac{2.6\cdot 10^{-5}}{6}=4.3\cdot 10^{-6}$										(S87) [17]
$F_{A}=\frac{{\left({\hat{s}}_{1.A}\right)}^{2}}{{\left({\hat{s}}_{2}\right)}^{2}}=\frac{1.03\cdot 10^{-4}}{4.3\cdot 10^{-6}}=24.2$										(S88) [17]
$F_{A^{2}}=\frac{{\left({\hat{s}}_{1.A^{2}}\right)}^{2}}{{\left({\hat{s}}_{2}\right)}^{2}}=\frac{1.3\cdot 10^{-5}}{4.3\cdot 10^{-6}}=3.0$										(S91) [17]
$p_{A}=p\left(F_{A},\vartheta_{1}=df_{effect.A},\vartheta_{2}=df_{error}\right)=0.003$										(A20)
$p_{A^{2}}=p\left(F_{A^{2}},\vartheta_{1}=df_{effect.A^{2}},\vartheta_{2}=df_{error}\right)=0.134$										(A21)

**Table A14:** The results of calculations in the Statistica 13 environment of the *p*-value in the ANOVA and power of the test of the ANOVA for the effect value (see Table A8) with the names of the effects used in Figure 1b and Figure 2b for “ALL” plan.

Name	*p*-Value in the ANOVA					Power of ANOVA			
	SS	df	MS	F	p	Fcr	δ	β	Power
(1) FORM (L)	0.003391	1	0.00	2751.582673	1.0245 × 10^−44^	4.038393	741.58	0.00	1.00
(2) RPET content (L)	0.000085	1	0.00	68.92770008	6.615 × 10^−11^	4.038393	18.58	0.011769	0.99
(2) RPET content (Q)	0.000013	1	0.00	10.91877454	0.001784539	4.038393	2.94	0.609712	0.39
1L vs. 2L	0.001028	1	0.00	833.919994	2.001 × 10^−32^	4.038393	224.75	0	1.00
Error	0.000060	49	0.000001						
Total	0.003265	53							

**Table A15:** The results of calculations in the Statistica 13 environment of the *p*-value in the ANOVA and power of the test of the ANOVA for the effect value (see Table A10) with the names of the effects used in Figure 1b and Figure 2b for “RPET” plan.

Name	*p*-Value in the ANOVA					Power of ANOVA			
	SS	df	MS	F	p	Fcr	δ	β	Power
(1) FORM (L)	0.001170	1	0.00	488.3389808	1.074 × 10^−11^	4.667193	43.96	0.00	1.00
(2) RPET content (L)	0.000038	1	0.00	15.65740176	0.001639911	4.667193	1.41	0.803683	0.20
(2) RPET content (Q)	0.000007	1	0.00	2.818062553	0.117069743	4.667193	0.25	0.924981	0.08
1L vs. 2L	0.000445	1	0.00	185.9005796	4.454 × 10^−9^	4.667193	16.73	0.034914	0.97
Error	0.000031	13	0.000002						
Total	0.002036	17							

**Table A16:** The results of calculations in the Statistica 13 environment of the *p*-value in the ANOVA and power of the test of the ANOVA for the effect value (see Table A12) with the names of the effects used in Figure 1c, Figure 2c and Figure 3.

Name	*p*-Value in the ANOVA					Power of ANOVA			
	SS	df	MS	F	p	Fcr	δ	β	Power
(1) RPET content (L)	0.000000	1	0.00	6.862579	0.012927	4.121338	5.25	0.39	0.61
(1) RPET content(Q)	0.000002	1	0.00	38.47619	4.18 × 10^−7^	4.121338	29.41	0.00	1.00
(2) power of heating lamps (L)	0.000015	1	0.00	336.2664	1.58 × 10^−19^	4.121338	257.04	0.00	1.00
(2) power of heating lamps (Q)	0.000001	1	0.00	27.46175	7.75 × 10^−6^	4.121338	20.99	0.01	0.99
(3) power of cooling fans (L)	0.000001	1	0.00	19.83285	8.26 × 10^−5^	4.121338	15.16	0.03	0.97
(3) power of cooling fans (Q)	0.000001	1	0.00	32.73736	1.8 × 10^−6^	4.121338	25.02	0.00	1.00
1L vs. 2L	0.000002	1	0.00	49.41057	3.5 × 10^−8^	4.121338	37.77	0.00	1.00
1L vs. 3L	0.000000	1	0.00	5.490063	0.024936	4.121338	4.20	0.49	0.51
2L vs. 3L	0.000002	1	0.00	41.5186	2.02 × 10^−7^	4.121338	31.74	0.00	1.00
Error	0.000002	35	0.000000						
Total	0.000024	44							

### Appendix B.3. The Method of Calculating the Influence of the Power of Heating Lamps and the Power of Cooling Fans on the Change in the Influence of rPET Content on the Properties of the Bottle Relative to the Properties of the Preform for Plans (e), and (f) (Table A1) Based on the Example of Density Measurements

The procedure for calculating the effect of the heating lamp power on the change in the effect of the rPET content on the properties of the bottle material relative to the preform material for (e) measurement plan (Table A1) (similarly, it will be for the influence of the cooling fan power for (f) measurement plan (Table A1)), which consisted of 5 steps (shown on the example of density measurements):

Step 1: Calculation of the standardized density value for each measurement series (Table A17) in (e) measurement plan (Table A1). The standardized value is the distance of one measurement series from the mean of the whole series, divided by the standard deviation of the distribution of the whole series (Table A17).
Step 2: Testing the homogeneity of variances between groups (Table A18) and the significance of differences between means (Table A19) for (e) measurement plan (Table A1).
Step 3: Testing the differences between the means for all pairs of series in (e) measurement plan (Table A1) using the Bonferroni post hoc test (Table A19).
Step 4: Calculation of the statistically significant (by Bonferroni test shown in Table A23) linear regression coefficient value (Table A24) based on the definition and value of the regression coefficient for standardized values (Table A21 and Table A22).
Step 5: Calculation of the change in the impact of RPET content from LAMPS (shown in Figure 1b and Figure 2b) in two ways (Table A25), i.e., for only 25% rPET content and for the arithmetic mean of the rPET content. If the analyzed effect of the change in the power of the heating lamps on the change in the effect of the rPET content was statistically insignificant for 25% rPET, the effect calculated for the arithmetic mean was taken into account.

**Table A17:** The standardized density value for each measurement series in (e)’s measurement plan (Table A1).

Name	FORM	RPET	LAMPS	Density (g/cm^3^)	
				Mean	Standardized
p0.0	preform	0	p	1.3409	−1.4869
p0.25	preform	0.25	p	1.3427	−1.3249
p0.5	preform	0.5	p	1.3492	−0.7257
A6	bottle	0.25	−0.1	1.3644	0.6684
A3	bottle	0	0	1.3648	0.7051
A8	bottle	0.25	0	1.3647	0.6989
A13	bottle	0.5	0	1.3648	0.7051
A10	bottle	0.25	0.1	1.3654	0.7601

**Table A18:** The homogeneity of variances between groups for (e) measurement plan (Table A1).

Levene’s Test of Homogeneity of Variance. Effects Are Significant for *p* < 0.05.							
SS Effect	df Effect	MS Effect	SS Error	df Error	MS Error	F	p
0.000009	7	0.000001	0.00001	16	0.000001	2.391462	0.070485

**Table A19:** The ANOVA significance of differences between means for (e) measurement plan (Table A1).

Analysis of Variance (ANOVA). Effects Are Significant for *p* < 0.05.							
SS Effect	df Effect	MS Effect	SS Error	df Error	MS Error	F	p
0.002	7	0.000	0.00003	16	0.000002	220.3659	0.000000

**Table A20:** The Bonferroni post hoc test results of the differences between the means for all pairs of series in the (e) measurement plan (Table A1).

Post Hoc Bonferroni Test. Differences Between Groups Are Significant for *p* < 0.05.								
Density (g/cm^3^)	{1}	{2}	{3}	{4}	{5}	{6}	{7}	{8}
A3 {1}		0.999986	1.000000	0.999779	1.000000	0.000000	0.000000	0.000000
A6 {2}	0.999986		0.999996	0.994238	0.999986	0.000000	0.000000	0.000000
A8 {3}	1.000000	0.999996		0.999557	1.000000	0.000000	0.000000	0.000000
A10 {4}	0.999779	0.994238	0.999557		0.999779	0.000000	0.000000	0.000000
A13 {5}	1.000000	0.999986	1.000000	0.999779		0.000000	0.000000	0.000000
p0.0 {6}	0.000000	0.000000	0.000000	0.000000	0.000000		0.880178	0.000142
p0.25 {7}	0.000000	0.000000	0.000000	0.000000	0.000000	0.880178		0.002011
p0.5 {8}	0.000000	0.000000	0.000000	0.000000	0.000000	0.000142	0.002011	

**Table A21:** Calculation formulas of regression coefficients for standardized value (Table A17) in (e) measurement plan (Table A1).

Regression Coefficients: Bottle—Preform (FANS = 0)				
Calculation Formula of Regression Coefficients for Standardized Value (Table A7)		Power of Heating Lamps		
		−0.1	0	0.1
rPET content	0		r1 = A3 − p0.0	
	0.25	r2 = A6 − p0.25	r3 = A8 − p0.25	r4 = A10 − p0.25
	0.5		r5 = A13 − p0.5	

**Table A22:** Values of regression coefficients for standardized value (Table A17) in (e) measurement plan (Table A1).

Regression Coefficients: Bottle—Preform (FANS = 0)				
Linear Regression Coefficient Value (Table A21)		Power of Heating Lamps		
		−0.1	0	0.1
rPET content	0		2.191965	
	0.25	1.993252	2.023823	2.084966
	0.5		1.430739	

**Table A23:** *p*-values of the Bonferroni test (Table A20) for each pair of series in (e) measurement plan (Table A1).

Post Hoc Bonferroni Test: Bottle—Preform (FANS = 0)				
*p*-Value from Bonferroni’s Test (Table A20)		Power of Heating Lamps		
		−0.1	0	0.1
rPET content	0		0.000	
	0.25	0.000	0.000	0.000
	0.5		0.000	

**Table A24:** Values of the statistically significant (by Bonferroni test shown in Table A23) regression coefficients for standardized value (Table A17) in (e) measurement plan (Table A1) and the arithmetic mean of the linear regression coefficient (defined according to Table A21) for all rPET contents of individual heating lamp powers.

Regression Coefficients: Bottle—Preform (FANS = 0)				
Statistically Significant Linear Regression Coefficient Value (by Bonferroni Test)		Power of Heating Lamps		
		−0.1	0	0.1
rPET content	0		2.191965	
	0.25	1.993252	2.023823	2.084966
	0.5		1.430739	
	arithmetic mean (see Table A21)	r2 = 1.993252	(r1 + r3 + r5)/3 = 1.882176	r4 = 2.084966

**Table A25:** Calculation of the change in the impact of RPET content from LAMPS (shown in Figure 1b and Figure 2b) in two ways, i.e., for only 25% rPET content and for the arithmetic mean of the rPET content.

Influence of “Power of Heating LAMPS” on the Influence of “RPET Content”			
Method of Taking into Account the rPET Content	Effect (see Figure 1b and Figure 2b)	Method of Calculation (see Table A21)	Change in Impact of RPET Content from LAMPS (see Figure 1b and Figure 2b)
Based on the 25% of rPET content (p0.25)	(2) RPET content (L)	r4 − r2	0.091714
	(2) RPET content (Q)	r3 − (r4 + r2)/2	−0.015286
Based on arithmetic mean for rPET content	(2) RPET content (L)	r4 − r2	0.091714
	(2) RPET content (Q)	(r1 + r3 + r5)/3 − (r4 + r2)/2	−0.156933

The calculated *p*-values and power of ANOVA for every effect for each experimental design of each dependent variable are presented in the Supporting Information in Table S7. The calculated adjusted R^2 values (the parameter of the fit of the model to the measured data) for each experimental design of each dependent variable are also presented in the Supporting Information in Table S7.

From Table S7, it follows that from the analysis of *p*-values, ANOVA test power, and adjusted R^2, it can be concluded that the adopted statistical models for almost all experimental designs for all dependent variables can only be used for preliminary qualitative analysis.

## Appendix C

### Appendix C.1. The Method of Interpreting the Statistical Significance Effects Shown in Figure 1a and Figure 2a (Plan (b) in Table A1) Based on the Example of Density Measurements

The procedure for interpreting the statistical significance effects shown in Figure 1a and Figure 2a (plan (b) in Table A1), based on the example of density measurements (Figure 1a), consisted of 5 steps:

Step 1: From Figure 1a, determine the sign (−, 0, +) of the linear main effect and the quadratic main effect of the influence of rPET content on the density of the preform material.
Step 2: From Figure 1a, determine whether the power of the ANOVA test for a given effect is greater than 80%.
Step 3: From Figure 1a, determine whether the absolute value of the quadratic main effect is greater than 1/4 or 1/2 of the absolute value of the linear main effect.
Step 4: Make a symbolic interpretation of the effect of rPET content on the density of the preform material in terms of the linear and quadratic effect (interpret the linear main effect and the quadratic main effect against the linear main effect)—see Table A26.
Step 5: Draw final conclusions from the symbolic interpretation of the linear main effect of the effect of rPET content on the density of the preform material (see Table A27). Assign the conclusion regarding the quadratic main effect against the linear main effect to one of the four different cases (see Table A28).

**Table A26:** Symbolic interpretation of the linear and quadratic influence of rPET content on the density of the preform material shown in Figure 1a (an explanation of the interpretation of the quadratic effect against the linear effect is shown in Figure S1 in [17]). Symbolic interpretation of the linear and quadratic influence of rPET content on all measured microscopic features of the preform material is shown in Table S1.

Study	Figure	Feature	LINEAR EFFECT		QUADRATIC EFFECT							
			A (RPET Content)	Power of the Test > 0.8	A^2^ (RPET Content)	Power of the Test > 0.8	|A^2^| > |¼ A|	|A^2^| > |½ A|	Extreme	Trend of Change for Low Values of Factor A	Trend of Change for High Values of Factor A	|Trend of Change for Low Values of Factor A| vs. |Trend of Change for High Values of Factor A|
Physical and thermal properties	1a	Density (ρ)	(+)	YES	(0)	NO	n.a.4	n.a.4	n.a.4	n.a.4	n.a.4	n.a.4

n.a.4 means no conclusion can be drawn for the quadratic effect of a given factor as it is zero.

**Table A27:** A summary of the analyzed main linear effect shown in Table A26 (a summary of the analyzed main linear effect of rPET content on all measured microscopic features of the preform material is shown in Table 1).

Dependent Variables			Independent Variables			
			SBM Process	RPET Content	Power of Heating Lamps	Power of Cooling Fans
Preform (Table S1)	Physical and thermal properties	Density	N/A	+	N/A	N/A

“+” is the dependent variable increases as the independent variable increases. N/A means “not applicable”.

**Table A28:** Division of density due to linearity and non-linearity of changes concerning independent variables shown in Table A26 (division of all dependent variables due to linearity and non-linearity of changes concerning independent variables is shown in Table 2).

Object	Independent Variable	Trend Of Changes In The Dependent Variable			
		1. Linear Variability of the Dependent Variable in Terms of the Independent Variable	Non-Linear Variation of the Dependent Variable Within the Range of the Independent Variable		
			2. No Change in the Sign of the Trend of Changes in the Dependent Variable	3. No Clear Evidence of a Change in the Sign of the Trend of Changes in the Dependent Variable.	4. Change in the Sign of the Trend of Changes in the Dependent Variable.
Preform (Table S1)	rPET content	(1) Density (+;0)	-	-	-

The symbols in parentheses refer to the sign of the effects (linear; quadratic).

### Appendix C.2. The Method of Interpreting the Statistical Significance Effects Shown in Figure 1b and Figure 2b (Plans (c), (d), (e), (f) in Table A1) Based on the Example of Density Measurements

The procedure for interpreting the statistical significance effects shown in Figure 1b and Figure 2b (plans (c), (d), (e), (f) in Table A1) based on the example of density measurements (Figure 1b) consisted of 10 steps:

Step 1: From Figure 1b, determine the sign (−, 0, +) of the linear two-way interaction effect for the influence of the SBM process and the rPET content (A × B) on the density of the bottle material relative to the density of the preform material. From Figure 1b, determine whether the absolute value of the linear main effect of the influence of the SBM process (A) is greater than the absolute value of the linear two-way interaction effect (A × B), and determine whether the absolute value of the linear main effect of the influence of rPET content (B) is greater than the absolute value of the linear two-way interaction effect (A × B) (see Table A29).
Step 2: From Figure 1b, determine the sign (−, 0, +) of the linear main effect of the influence of the SBM process (A) and of the linear main effect of the influence of rPET content (B) on the density of the bottle material relative to the density of the preform material. From Figure 1b, determine whether the absolute value of the linear main effect of the influence of the SBM process (A) is greater than the absolute value of the linear main effect of the influence of rPET content (B) (see Table A29).
Step 3: Based on Table A30, make a symbolic interpretation of the linear effect of the SBM process (A) in terms of the level of Factor B (i.e., low level of Factor B (B−), which means 0% of rPER content, while the high level of Factor B (B+) means 50%of rPET content). Make a symbolic interpretation of the linear effect of the rPET content (B) in terms of the level of Factor A (i.e., low level of Factor A (A−), which means preform, while the high level of Factor A (A+) means bottle) (see Table A29).
Step 4: From Figure 1b, determine whether the power of the ANOVA test for a given linear effect and main quadratic effect is greater than 80% (see Table A29).
Step 5: Draw final conclusions from the symbolic interpretation of the linear main effect of the effect of the SBM process, and of rPET content on the density of the bottle material relative to the density of the preform material (see Table A31).
Step 6: From Figure 1b, determine the sign (−, 0, +) of the quadratic main effect of the influence of rPET content on the density of the bottle material relative to the density of the preform material (see Table A32).
Step 7: From Figure 1b, determine whether the absolute value of the quadratic main effect is greater than 1/4 or 1/2 of the absolute value of the linear main effect of the influence of rPET content on the density of the bottle material relative to the density of the preform material (see Table A32).
Step 8: Make a symbolic interpretation of the effect of rPET content on the density of the bottle material relative to the density of the preform material in terms of the quadratic effect (interpret the quadratic main effect against the linear main effect)—see Table A32.
Step 9: Assign the conclusion regarding the quadratic main effect against the linear main effect to one of the four different cases (see Table A33).
Step 10: Determine the conclusion regarding the impact of the power of the heating lamps and the power of the cooling fans on the change in the effect of the rPET content on the density bottle material relative to the density of the preform material in the SBM process—see Table A29 (see Table A25 for the impact of the power of the heating lamps on the change in the effect of the rPET content).

**Table A29:** The symbolic interpretation of the linear effect of the SBM process (A) and rPET content (B) on the density of the bottle relative to the preform shown in Figure 1b—symbolic interpretation of linear two-way interactions from the graph presented in Figure 1b necessary for the interpretation of the linear main effects presented in Figure 1b was based on the Table A30. Symbolic interpretation of the linear influence of the SBM process and rPET content on all measured microscopic features of the bottle material relative to the preform material is shown in Table S2.

Study	Figure	Feature		Linear Effects of the SBM Process (A) and RPET Content (B)																						
				Linear Two-Way Interactions							Linear Main Effects with Respect to Linear Two-Way Interactions															
				A × B							A (SBM Process—FORM)							B (RPET Content)								
				A (SBM Process)	B (RPET Content)	A × B	|A| vs. |B|	|A × B| vs. |A|	|A × B| vs. |B|	The Power of the Test > 0.8	Quantitative Independence from Factor B	Trend of Change for a Low Level of Factor B: (B−)	Trend of Change for a High Level of Factor B: (B+)	Relative Absolute Value of the Trends for Low and High Levels of Factor B: |B−| vs. |B+|	Qualitative Independence from Factors B	Qualitative Main Effect of Factor A	Power of the Test > 0.8	Quantitative Independence from Factor A	Trend of Change for a Low Level of Factor A: (A−)	Trend of Change for a High Level of Factor A: (A+)	Relative Absolute Value of the Trends for Low and High Levels of Factor A: |A−| vs. |A+|	Qualitative Independence from Factors A	Qualitative Main Effect of Factor B	Power of the Test > 0.8	Change of Impact on the Feature of RPER Content from the Power of LAMPS (Table A1e)	Change of Impact on the Feature of RPER Content from the Power of FANS (Table A1f)
Physical and thermal properties	1b	Density (ρ)	ALL	(+)	(+)	(−)	>	<	=	YES	NO	(+)	(+)	>	YES	(+)	YES	NO	(+)	0	n.a.2	YES	(+)	YES	+	-
			RPET	(+)	(+)	(−)	>	<	=	YES	NO	(+)	(+)	>	YES	(+)	YES	NO	(+)	0	n.a.2	YES	(+)	NO	n.a.7	n.a.7

n.a.2 means not applicable because there is at least one zero trend; n.a.7 means no conclusion can be drawn because the lamp power and fan power settings were constant (the middle value of the lamp power variability and the fan power variability).

**Table A30:** Influence of the value and sign of the two-way interaction A × B on the interpretation of the Factor A effect depending on the level of Factor B, and vice versa (influences of any combination of the value and sign of the two-way interaction A × B on the interpretation of the Factor A effect depending on the level of Factor B, and vice versa is described in Table S2 in [17]).

No. [17]	Effect Sign			Relative Absolute Values of Effects			Factor A (Dependent Variable A)					Factor B (Dependent Variable B)				
	A	B	A × B	|A| vs. |B|	|A × B| vs. |A|	|A × B| vs. |B|	Trend of Change for a Low Level of Factor B: (B−)	Trend of Change for a High Level of Factor B: (B+)	Initial Value, i.e., in A−, for Low and High Levels of Factor B: (B−) vs. (B+)	Trends of Change for Low and High Levels of Factor B Intersect in the Domain of Change of Factor A	Relative Absolute Value of the Trends for Low and High Levels of Factor B: |B−| vs. |B+|	Trend of Change for a Low Level of Factor A: (A−)	Trend of Change for a High Level of Factor A: (A+)	Initial Value, i.e., in B−, for Low and High Levels of Factor A: (A−) vs. (A+)	Trends of Change for Low and High Levels of Factor A Intersect in the Domain of Change of Factor B	Relative Absolute Value of the Trends for Low and High Levels of Factor A: |A−| vs. |A+|
106	+	+	-	>	<	=	(+)	(+)	<	(A+)	(>)	(+)	(0)	<	NO	n.a.2

(n.a.2) means not applicable because there is at least one zero trend; (0) is a zero trend of change; (+) is a positive trend of change; (>) is a faster trend of change; NO means trends of changes for low and high levels of the second factor do not intersect in the domain of change of the first factor; (A+) means trends of changes for low and high levels of Factor B intersect at the point of high level of Factor A (A−).

**Table A31:** Summary of the analyzed main linear effects shown in Table A29 (summary of the analyzed main linear effect of SBM process and rPET content on all measured microscopic features of the bottle material relative to the preform material is shown in Table 1).

Dependent Variables			Independent Variables				
			SBM Process	RPET Content		Power of Heating Lamps	Power of Cooling Fans
Bottle vs. preform (Table S2)	Physical and thermal properties	Density	+	Preform +	Bottle 0	N/A	N/A

“+” is the dependent variable increasing as the independent variable increases; “0” is the dependent variable not changing statistically significantly as the independent variable increases. N/A means “not applicable”.

**Table A32:** Symbolic interpretation of the quadratic influence of the SBM process (A) and rPET content (B) on the density of the bottle in relation to the preform shown in Figure 1b—symbolic interpretation of the quadratic effects in relation to the linear effects was based on the idea presented in Figure S1 describing the interpretation of quadratic effects versus linear effects presented in the first part of the article [17]. Symbolic interpretation of the quadratic influence of the SBM process and rPET content on all measured microscopic features of the bottle material relative to the preform material is shown in Table S3.

Study	Figure	Feature		Quadratic Main Effects with Respect to Linear Main Effects of RPET Content (B)										
				Linear Effect			Sign of Quadratic Main Effect	Extreme	Trend of Change for Low Values of Factor B	Trend of Change for High Values of Factor B	|Trend of Change for Low Values of Factor B| vs. |Trend of Change for High Values of Factor B|	Power of the Test > 0.8	Change of Impact on Feature of RPER Content from	
				|B²| > |¼ B|	|B²| > |½ B|	Sign of Linear Main Effect							Power of LAMPS (Table A1e)	Power of FANS (Table A1f)
Physical and thermal properties	1b	Density (ρ)	ALL	NO	NO	(+)	(−)	n.a.5	(+)	(+)	<	NO	-	-
			RPET	n.a.4	n.a.4	(+)	0	n.a.4	n.a.4	n.a.4	n.a.4	NO	n.a.7	n.a.7

n.a.4 means no conclusion can be drawn for the quadratic effect of a given factor as it is zero; n.a.5 means no extreme because the quadratic effect is smaller than ¼ of the linear effect; n.a.7 means no conclusion can be drawn because the lamp power and fan power settings were constant (the middle value of the lamp power variability and the fan power variability).

**Table A33:** Division of density due to linearity and non-linearity of changes concerning independent variables shown in Table A32 (division of all dependent variables due to linearity and non-linearity of changes concerning independent variables is shown in Table 2).

Object	Independent Variable	Trend of Changes in the Dependent Variable			
		1. Linear Variability of the Dependent Variable in Terms of the Independent Variable	Non-Linear Variation of the Dependent Variable Within the Range of the Independent Variable		
			2. No Change in the Sign of the Trend of Changes in the Dependent Variable	3. No Clear Evidence of a Change in the Sign of the Trend of Changes in the Dependent Variable.	4. Change in the Sign of the Trend of Changes in the Dependent Variable.
Bottle and Preform (Table S3)	rPET content	(1) Density “RPET” (+;0)	(1) Density “ALL” (+;−)	-	-

The symbols in parentheses refer to the sign of the effects (linear; quadratic).

### Appendix C.3. The Method of Interpreting the Statistical Significance Effects Shown in Figure 1c, Figure 2c and Figure 3 (Plan (a) in Table A1) Based on the Example of Density Measurements

The procedure for interpreting the statistical significance effects shown in Figure 1c and Figure 2c (plan (a) in Table A1), based on the example of density measurements (Figure 1c), consisted of 9 steps:

Step 1: From Figure 1c, determine the sign (−, 0, +) of the linear two-way interaction effects for the influence of the rPET content and the power of heating lamps (A × B), of the rPET content and the power of cooling fans (A × C) and of the power of heating lamps and the power of cooling fans (B × C) on the density of the bottle material. From Figure 1c, determine whether the absolute value of the linear main effect of the influence of rPET content (A) is greater than the absolute value of the linear two-way interaction effect (A × B), and determine whether the absolute value of the linear main effect of the influence of the power of heating lamps (B) is greater than the absolute value of the linear two-way interaction effect (A × B). The same procedure must be followed for the main linear effects A and C in relation to the linear interaction effect A × C and for the main linear effects B and C in relation to the linear interaction effect A × C (see Table A34).
Step 2: From Figure 1c, determine the sign (−, 0, +) of the linear main effect of the influence of the rPET content (A) and of the linear main effect of the influence of the power of heating lamps (B) on the density of the bottle material. From Figure 1c, determine whether the absolute value of the linear main effect of the influence of rPET content (A) is greater than the absolute value of the linear main effect of the influence of rPET content (B). The same procedure must be followed for the main linear effects A and C and for the main linear effects B and C (see Table A34).
Step 3: Based on Table A35, make a symbolic interpretation of the linear effect of the rPET content (A) in terms of the level of Factor B (i.e., a low level of Factor B (B−), which means low power of heating lamps, while the high level of Factor B (B+) means high power of heating lamps). Make a symbolic interpretation of the linear effect of the power of heating lamps (B) in terms of the level of Factor A (i.e., low level of Factor A (A−), which means 0% of rPER content, while a high level of Factor A (A+) means 50% of rPET content). The same procedure must be followed for the main linear effects A and C and for the main linear effects B and C (see Table A36).
Step 4: From Figure 1c, determine whether the power of the ANOVA test for a given linear effect and main quadratic effect is greater than 80% (see Table A36).
Step 5: Draw final conclusions from the symbolic interpretation of the linear main effect of the effect of the rPET content, the power of heating lamps, and the power of cooling fans on the density of the bottle material (see Table A37).
Step 6: From Figure 1c, determine the sign (−, 0, +) of the quadratic main effect of the influence of the rPET content, of the power of heating lamps, and of the power of cooling fans on the density of the bottle material relative to the density of the preform material (see Table A38).
Step 7: From Figure 1c, determine whether the absolute value of the quadratic main effect of the rPET content is greater than 1/4 or 1/2 of the absolute value of the linear main effect of the influence of rPET content on the density of the bottle material. The same procedure must be followed for the power of heating lamps and for the power of cooling fans (see Table A38).
Step 8: Make a symbolic interpretation of the effect of rPET content, of the effect of the power of heating lamps, and of the effect of the power of cooling fans on the density of the bottle material in terms of the quadratic effect (interpret the quadratic main effect against the linear main effect)—see Table A38.
Step 9: Assign the conclusion regarding the quadratic main effect against the linear main effect to one of the four different cases (see Table A39).

**Table A34:** Symbolic interpretation of the linear two-way interactions from the graph presented in Figure 1c of the influence of rPET content (A), the power of heating lamps (B), and the power of cooling fans (C) on the density of the bottle, necessary to interpret the linear main effects presented in Table A36 based on the Table A35 describing the interpretation of the two-factor cross-effects presented in the first part of the article [17]. Symbolic interpretation of the linear two-way interactions from the graph presented in Figure 1c, Figure 2c and Figure 3 of the influence of rPET content (A), the power of heating lamps (B), and the power of cooling fans (C) on all measured microscopic and macroscopic features of the bottle, necessary to interpret the linear main effects presented in Table S5 based on the Table S2 (describing the interpretation of the two-factor cross-effects presented in the first part of the article [17]) is presented in Table S4.

Study	Figure	Feature	Linear Two-Way Interactions																				
			A × B							A × C							B × C						
			A (PRET Content)	B (Power of LAMPS)	A × B	|A| vs. |B|	|A × B| vs. |A|	|A × B| vs. |B|	Power of the Test > 0.8	A (PRET Content)	C (Power of FANS)	A × C	|A| vs. |C|	|A × C| vs. |A|	|A × C| vs. |C|	Power of the Test > 0.8	B (Power of LAMPS)	C (Power of FANS)	B × C	|B| vs. |C|	|B × C| vs. |B|	|B × C| vs. |C|	Power of the Test > 0.8
Physical and thermal properties	1c	Density (ρ)	(−)	(+)	(+)	<	>	<	YES	(−)	(−)	(+)	<	=	<	NO	(+)	(−)	(+)	>	<	>	YES

**Table A35:** Influence of the value and sign of the two-way interaction A × B (No. 151 from Table S2 [17]) on the interpretation of the Factor A effect depending on the level of Factor B, and vice versa—and analogously for the two-way interaction A × C (No. 77 from Table S2 [17]) and B × C (No. 129 from Table S2 [17]). Influences of any combination of the value and sign of the two-way interaction A × B on the interpretation of the Factor A effect depending on the level of Factor B, and vice versa, are described in Table S2 in [17].

No. [17]	Effect Sign			Relative Absolute Values of Effects			Factor A (Dependent Variable A)					Factor B (Dependent Variable B)				
	A	B	A × B	|A| vs. |B|	|A × B| vs. |A|	|A × B| vs. |B|	Trend of Change for a Low Level of Factor B: (B−)	Trend of Change for a High Level of Factor B: (B+)	Initial Value, i.e., in A−, for Low and High Levels of Factor B: (B−) vs. (B+)	Trends of Change for Low and High Levels of Factor B Intersect in the Domain of Change of Factor A	Relative Absolute Value of the Trends for Low and High Levels of Factor B: |B−| vs. |B+|	Trend of Change for a Low Level of Factor A: (A−)	Trend of Change for a High Level of Factor A: (A+)	Initial Value, i.e., in B−, for Low and High Levels of Factor A: (A−) vs. (A+)	Trends of Change for Low and High Levels of Factor A Intersect in the Domain of Change of Factor B	Relative Absolute Value of the Trends for Low and High Levels of Factor A: |A−| vs. |A+|
151 (A × B)	−	+	+	<	>	<	(−)	(+)	<	NO	(>)	(+)	(+)	>	YES	(<)
77 (A × C)	−	−	+	<	=	<	(−)	(0)	>	NO	n.a.2	(−)	(−)	>	(B+)	(>)
129 (B × C)	+	−	+	>	<	>	(+)	(+)	>	YES	(<)	(−)	(+)	<	NO	(>)

(n.a.2) means not applicable because there is at least one zero trend; (0) is a zero trend of change; (+) is a positive trend of change; (−) is a negative trend of change; (>) is a faster trend of change; (<) is a slower trend of change; YES means trends of changes for low and high levels of the second factor intersect in the domain of change of the first factor; NO means trends of changes for low and high levels of the second factor do not intersect in the domain of change of the first factor; (B+) means trends of changes for low and high levels of Factor A intersect at the point of high level of Factor B (B−).

**Table A36:** Symbolic interpretation of the linear main effects from the graphs presented in Figure 1c in relation to the linear two-way effects presented in Table A34 and Table A35 describing the interpretation of the two-way cross effects of the influence of rPET (A) content, the power of heating lamps (B), and the power of cooling fans (C) on the density of the bottle. Symbolic interpretation of the linear main effects from the graphs presented in Figure 1c, Figure 2c and Figure 3 in relation to the linear two-way effects presented in Tables S2 and S4 [17] describing the interpretation of the two-way cross effects of the influence of rPET (A) content, the power of heating lamps (B), and the power of cooling fans (C) on the microscopic features of the bottle is presented in Table S5.

Study	Figure	Feature	Linear Main Effects with Respect to Linear Two-Way Interactions																																
			A (RPET Content)											B (Power of LAMPS)											C (Power of FANS)										
			A × B				A × C				Qualitative Independence from Factors B and C	Qualitative Main Effect of Factor A	Power of the Test > 0.8	A × B				B × C				Qualitative Independence from Factors A and C	Qualitative Main Effect of Factor B	Power of the Test > 0.8	A × C				B × C				Qualitative Independence from Factors A and B	Qualitative Main Effect of Factor C	Power of the Test > 0.8
			Quantitative Independence from Factor B	Trend of Change for a Low Level of Factor B: (B−)	Trend of Change for a High Level of Factor B: (B+)	Relative Absolute Value of the Trends for Low and High Levels of Factor B: |B−| vs. |B+|	Quantitative Independence from Factor C	Trend of Change for a Low Level of Factor C: (C−)	Trend of Change for a High Level of Factor C: (C+)	Relative Absolute Value of the Trends for Low and High Levels of Factor C: |C−| vs. |C+|				Quantitative Independence from Factor A	Trend of Change for a Low Level of Factor A: (A−)	Trend of Change for a High Level of Factor A: (A+)	Relative Absolute Value of the Trends for Low and High Levels of Factor A: |A−| vs. |A+|	Quantitative Independence from Factor C	Trend of Change for a Low Level of Factor C: (C−)	Trend of Change for a High Level of Factor C: (C+)	Relative Absolute Value of the Trends for Low and High Levels of Factor C: |C−| vs. |C+|				Quantitative Independence from Factor A	Trend of Change for a Low Level of Factor A: (A−)	Trend of Change for a High Level of Factor A: (A+)	Relative Absolute Value of the Trends for Low and High Levels of Factor A: |A−| vs. |A+|	Quantitative Independence from Factor B	Trend of Change for a Low Level of Factor B: (B−)	Trend of Change for a High Level of Factor B: (B+)	Relative Absolute Value of the Trends for Low and High Levels of Factor B: |B−| vs. |B+|			
Physical and thermal properties	1c	Density (ρ)	NO	(−)	(+)	>	NO	(−)	0	n.a.2	NO	n.a.3	NO	NO	(+)	(+)	<	NO	(+)	(+)	>	NO	n.a.3	YES	NO	(−)	(−)	>	NO	(−)	(+)	<	NO	n.a.3	YES

n.a.2 means not applicable because there is at least one zero trend; n.a.3 means no conclusion can be drawn for the linear effect of a given factor for any settings of the other two factors, i.e., the effect of a given factor should be analyzed in relation to the values of the other factors due to the overlapping of both interaction effects and low statistical power for the main effect of a given factor.

**Table A37:** Summary of the analyzed main linear effects shown in Table A36 (a summary of the analyzed main linear effect of the rPET content, power of heating lamps, and power of cooling fans on all measured microscopic features of the bottle material relative to the preform material is shown in Table 1).

Dependent Variables			Independent Variables					
			SBM Process	RPET Content		Power of Heating Lamps	Power of Cooling Fans	
Bottle (Table S5)	Physical and thermal properties	Density	N/A	LAMPS − | +	FANS − | 0	+	RPET −	LAMPS − | +

“+” is the dependent variable increasing as the independent variable increases; “−” is the dependent variable decreasing as the independent variable increases; “0” is the dependent variable not changing statistically significantly as the independent variable increases; “− | +” is the change in the dependent variable due to a change in the independent variable conditioned by the value of the “other” independent variable, and in the designation, the left side shows the effect of a change in the independent variable on the dependent variable, with a minimum setting of the “other” independent variable, while the right side shows the effect of a change in the independent variable on the dependent variable, at the maximum setting of the “other” independent variable, i.e., “min | max”. N/A means “not applicable”.

**Table A38:** Symbolic interpretation of the quadratic main effects from the graphs shown in Figure 1c of the influence of rPET content (A), the power of heating lamps (B), and the power of cooling fans (C) on the density of the bottle in relation to the linear main effects presented in Table A34 and Table A36 (based on the idea presented in Figures S1 and S2 describing the interpretation of quadratic effects in relation to the linear effects presented in the first part of the article [17]). Symbolic interpretation of the quadratic main effects from the graphs shown in Figure 1c, Figure 2c and Figure 3 of the influence of rPET content (A), the power of heating lamps (B), and the power of cooling fans (C) on all measured microscopic and macroscopic features of the bottle in relation to the linear main effects presented in Tables S4 and S5 (based on the idea presented in Figure S1 describing the interpretation of quadratic effects in relation to the linear effects presented in the first part of the article [17]) is presented in Table S6.

Study	Figure	Feature	Quadratic Main Effects with Respect to Linear Main Effects																													
			A² (RPET Content)										B² (Power of LAMPS)										C² (Power of FANS)									
			Does the Quadratic Effect Occur?	Linear Effect			Sign of Quadratic Main Effect	Extreme	Trend of Change for Low Values of Factor A	Trend of Change for High Values of Factor A	|Trend of Change for Low Values of Factor A| vs. |Trend of Change for High Values of Factor A|	Power of the Test > 0.8	Does the Quadratic Effect Occur?	Linear Effect			Sign of Quadratic Main Effect	Extreme	Trend of Change for Low Values of Factor B	Trend of Change for High Values of Factor B	|Trend of Change for Low Values of Factor B| vs. |Trend of Change for High Values of Factor B|	Power of the Test > 0.8	Does the Quadratic Effect Occur?	Linear Effect			Sign of Quadratic Main Effect	Extreme	Trend of Change for Low Values of Factor C	Trend of Change for High Values of Factor C	|Trend of Change for Low Values of Factor C| vs. |Trend of Change for High Values of Factor C|	Power of the Test > 0.8
				|A²| > |¼ A|	|A²| > |½ A|	Sign of Linear Main Effect								|B²| > |¼ B|	|B²| > |½ B|	Sign of Linear Main Effect								|C²| > |¼ C|	|C²| > |½ C|	Sign of Linear Main Effect						
Physical and thermal properties	1c	Density (ρ)	YES	YES	YES	(−)	(+)	MAX	(+)	(−)	<<	YES	YES	NO	NO	(+)	(+)	n.a.5	(+)	(+)	>	YES	YES	YES	YES	(−)	(+)	MAX	(+)	(−)	<<	YES

n.a.5 means no extreme because the quadratic effect is smaller than ¼ of the linear effect.

**Table A39:** Division of density due to linearity and non-linearity of changes concerning independent variables shown in Table A38 (a division of all dependent variables due to linearity and non-linearity of changes concerning independent variables is shown in Table 2).

Object	Independent Variable	Trend of Changes in the Dependent Variable			
		1. Linear Variability of the Dependent Variable in Terms of the Independent Variable	Non-Linear Variation of the Dependent Variable Within the Range of the Independent Variable		
			2. No Change in the Sign of the Trend of Changes in the Dependent Variable	3. No Clear Evidence of a Change in the Sign of the Trend of Changes in the Dependent Variable	4. Change in the Sign of the Trend of Changes in the Dependent Variable
Bottle (Table S6)	rPET content	-	-	-	(1) Density (−;+)
	Power of heating lamps	-	(1) Density (+;+)	-	-
	Power of cooling fans	-	-	-	(1) Density (−;+)

The symbols in parentheses refer to the sign of the effects (linear; quadratic).

## References

[1] Enache A.-C., Grecu I., Samoila P. (2024). Polyethylene Terephthalate (PET) Recycled by Catalytic Glycolysis: A Bridge toward Circular Economy Principles. Materials.

[2] Gnoffo C., Arrigo R., Frache A. (2024). An Upcycling Strategy for Polyethylene Terephthalate Fibers: All-Polymer Composites with Enhanced Mechanical Properties. J. Compos. Sci..

[3] Magazzù A., Marcuello C. (2023). Investigation of Soft Matter Nanomechanics by Atomic Force Microscopy and Optical Tweezers: A Comprehensive Review. Nanomaterials.

[4] Wawrzyniak P., Datta J. (2015). Characteristics of the blowing stages of poly (ethylene terephthalate) preforms in the blowing process with simultaneous stretching. Przem. Chem..

[5] Wawrzyniak P., Datta J. (2015). Stretch blow molding machines used for manufacturing PET bottles. Przem. Chem..

[6] Wawrzyniak P., Karaszewski W. (2020). A literature survey of the influence of preform reheating and stretch blow molding with hot mold process parameters on the properties of PET containers. Part I. Polimery.

[7] Wawrzyniak P., Karaszewski W. (2020). A literature survey of the influence of preform reheating and stretch blow molding with hot mold process parameters on the properties of PET containers. Part II. Polimery.

[8] Le A.-D., Gilblas R., Lucin V., Le Maoult Y., Schmidt F. (2022). Infrared heating modeling of recycled PET preforms in injection stretch blow molding process. Int. J. Therm. Sci..

[9] Yan S., Menary G., Nixon J. (2017). A novel methodology to characterize the constitutive behaviour of polyethylene terephthalate for the stretch blow moulding process. Mech. Mater..

[10] Awaja F., Pavel D. (2005). Injection stretch blow moulding process of reactive extruded recycled PET and virgin PET blends. Eur. Polym. J..

[11] Bordival M., Schmidt F.M., Le Maoult Y., Velay V. (2009). Optimization of preform temperature distribution for the stretch-blow molding of PET bottles: Infrared heating and blowing modeling. Polym. Eng. Sci..

[12] Thibault F., Malo A., Lanctot B., Diraddo R. (2007). Diraddo, Preform shape and operating condition optimization for the stretch blow molding process. Polym. Eng. Sci..

[13] Pham X.-T., Thibault F., Lim L.-T. (2004). Modeling and simulation of stretch blow molding of polyethylene terephthalate. Polym. Eng. Sci..

[14] McEvoy J.P., Armstrong C.G., Crawford R.J. (1998). Simulation of the stretch blow molding process of PET bottles. Adv. Polym. Technol..

[15] Wawrzyniak P., Karaszewski W. (2020). Blowing kinetics, pressure resistance, thermal stability, and relaxation of the amorphous phase of the PET container in the SBM process with hot and cold mold. Part I: Research methodology and results. Polymers.

[16] Wawrzyniak P., Karaszewski W. (2020). Blowing kinetics, pressure resistance, thermal stability, and relaxation of the amorphous phase of the PET container in the SBM process with hot and cold mold. Part II: Statistical Analysis and Interpretation of Tests. Polymers.

[17] Wawrzyniak P., Karaszewski W., Różański A. (2024). Effect of rPET Content and Preform Heating/Cooling Conditions in the Stretch Blow Molding Process on Microcavitation and Solid-State Post-Condensation of vPET-rPET Blend: Part I—Research Methodology and Results. Materials.

[18] Wawrzyniak P., Karaszewski W., Różański A. (2024). Cavitation and Solid-State Post-Condensation of Polyethylene Terephthalate: Literature Review. Materials.

[19] Heidrich D., Gehde M. (2022). The 3-Phase Structure of Polyesters (PBT, PET) after Isothermal and Non-Isothermal Crystallization. Polymers.

[20] Zhang Y., Ben Jar P.Y., Xue S., Li L. (2019). Quantification of strain-induced damage in semi-crystalline polymers: A review. J. Mater. Sci..

[21] Dong W., Zhao J., Li C., Guo M., Zhao D., Fan Q. (2002). Study of the amorphous phase in semicrystalline poIy(ethyIene terephthalate) via dynamie mechanical thermal analysis. Polym. Bull..

[22] Kansy J. (1996). Microcomputer program for analysis of positron annihilation lifetime spectra. Nucl. Instrum. Methods A.

[23] Sharma S.K., Pujari P.K. (2017). Role of free volume characteristics of polymer matrix in bulk physical properties of polymer nanocomposites: A review of positron annihilation lifetime studies. Prog. Polym. Sci..

[24] Jean Y.C., Van Horn J.D., Hung W.S., Lee K.R. (2013). Perspective of Positron Annihilation Spectroscopy in Polymers. Macromolecules.

[25] Lind J.H., Jones P.L., Pearsall G.W. (1986). A Positron-Annihilation Lifetime Study of Isotactic Polypropylene. J. Polym. Sci. Part A Polym. Chem..

[26] Nakanishi H., Jean Y.C., Smith E.G., Sandreczki T.C. (1989). Positronium Formation at Free-Volume Sites in the Amorphous Regions of Semicrystalline Peek. J. Polym. Sci. Part B Polym. Phys..

[27] de Daubeny R.P., Bunn C.W., Brown C.J. (1954). The crystal structure of polyethylene terephthalate. Proc. Roy. Soc..

[28] Gouissem L., Douibi A., Benachour D. (2014). The Evolution of Properties of Recycled Poly(ethylene terephthalate) as Function of Chain Extenders, the Extrusion Cycle and Heat Treatment. Polym. Sci. Ser. A.

[29] Galeski A. (2003). Strength and toughness of crystalline polymer systems. Prog. Polym. Sci..

[30] Rastogi R., Vellinga W.P., Rastogi S., Schick C., Meijer H.E.H. (2004). The Three-Phase Structure and Mechanical Properties of Poly(ethylene terephthalate). J. Polym. Sci. Part B Polym. Phys..

[31] Gantillon B., Spitz R., McKenna T.F. (2004). The Solid State Postcondensation of PET, 1. Macromol. Mater. Eng..

[32] Makarewicz C., Safandowska M., Idczak R., Rozanski A. (2022). Plastic Deformation of Polypropylene Studied by Positron Annihilation Lifetime Spectroscopy. Macromolecules.

[33] Hagihara H., Oishi A., Funabashi M., Kunioka M., Suda H. (2014). Free-volume hole size evaluated by positron annihilation lifetime spectroscopy in the amorphous part of poly(ethylene terephthalate) degraded by a weathering test. Polym. Degrad. Stab..

[34] Dyląg Z., Jakubowicz A., Orłoś Z. (2013). Wytrzymałość Materiałów Tom 2.

[35] Galeski A., Rozanski A. (2011). Cavitation during Drawing of Crystalline Polymers. Macromol. Symp..

[36] Ronkay F., Czigany T. (2006). Cavity formation and stress-oscillation during the tensile test of injection molded specimens made of PET. Polym. Bull..

[37] Olson B.G., Lin J., Nazarenko S., Jamieson A.M. (2003). Positron Annihilation Lifetime Spectroscopy of Poly(ethylene terephthalate): Contributions from Rigid and Mobile Amorphous Fractions. Macromolecules.

