# Supplementary material for: Effect of rPET Content and Preform Heating/Cooling Conditions in the Stretch Blow Molding Process on Microcavitation and Solid-State Post-Condensation of vPET-rPET Blend: Part II—Statistical Analysis and Interpretation of Tests
Source: Materials (Basel). 2024 Dec 25;18(1):36. doi: 10.3390/ma18010036 (PMC11721359; doi:10.3390/ma18010036)
Supplement: Supplementary file 1 [file materials-18-00036-s001.zip › materials-3377864-supplementary.pdf]

Supporting Information

Effect of rPET Content and Preform Heating/Cooling Condi-tions in the Stretch Blow Molding Process on Microcavitation and Solid-State Post-Condensation of vPET-rPET Blend: Part II —Statistical Analysis and Interpretation of Tests

S.1. Tabular Presentation of DOE results

The interpretation of the results of the microscopic and macroscopic features tests is presented symbolically in Tables: S1 (the linear and quadratic influence of rPET content on the microscopic features of the preform shown in Figures 1a, and 2a), S2 (the linear influence of the SBM process and rPET content on the microscopic features of the bottle material in relation to the preform material shown in Figures 1b, and 2b), S3 (the quadratic effect of the SBM process and rPET content on the microscopic properties of the bottle in relation to the preform shown in Figures 1b, and 2b), S4 (the linear two-way interactions from the graphs presented in Figures 1c, and 2c showing the influence of rPET content, the power of heating lamps, and the power of cooling fans on the microscopic features of the bottle, necessary to interpret the linear main effects presented in Table S5), S5 (the linear main effects of the influence of rPET content, the power of heating lamps, and the power of cooling fans on the microscopic and macroscopic features of the bottle from the graphs presented in Figures 1c, and 2c with reference to the linear two-way effects presented in Table S4), and S6 (the quadratic main effects from the graphs shown in Figures 1c, and 2c of the influence of rPET content, the power of heating lampa, and the power of cooling fan on the microscopic and macroscopic features of the bottle in relation to the linear main effects presented in Tables S4 and S5). A full statistical interpretation of Figures 1, and 2 used to create Tables S1, S2, S3, S4, S5, and S6 is presented below the tables, in section S.2.

The verbal description of the interpretation will not be presented for all dependent variables (the entire verbal description will be sent upon request). The description will be presented only for the most complex dependency occurring for the measurement of material density for the preform, the SBM process (bottle vs. preform), and the bottle.

Table S1. Symbolic interpretation of the linear and quadratic influence of rPET content on the microscopic features of the preform material shown in Figures 1a, and 2a

Study	Figure	Feature			LINEAR EFFECT: A (RPET content)	LINEAR EFFECT: Power of the test > 0.8	QUADRATIC EFFECT: A² (RPET content)	QUADRATIC EFFECT: Power of the test > 0.8	|A²|>|¼ A|	|A²|>|½ A|	Extreme	Trend of change for low values of Factor A	Trend of change for high values of Factor A	|Trend of change for low values of Factor A| vs. |Trend of change for high values of Factor A|
Physical and thermal properties	1a	Density (ρ)			(+)	YES	(0)	NO	n.a.4	n.a.4	n.a.4	n.a.4	n.a.4	n.a.4
		Crystallinity (X_C)			(+)	NO	(0)	NO	n.a.4	n.a.4	n.a.4	n.a.4	n.a.4	n.a.4
		Oriented amorphous phase			(+)	NO	(0)	NO	n.a.4	n.a.4	n.a.4	n.a.4	n.a.4	n.a.4
		Intrinsic viscosity (η)			(0)	NO	(0)	NO	n.a.4	n.a.4	n.a.4	n.a.4	n.a.4	n.a.4
		Glass transition temperature (T_g)			(0)	NO	(0)	NO	n.a.4	n.a.4	n.a.4	n.a.4	n.a.4	n.a.4
		Melting temperature (T_m)			(0)	NO	(0)	NO	n.a.4	n.a.4	n.a.4	n.a.4	n.a.4	n.a.4
PALS analysis	2a	τ_2	with σ_3	Mean	(0)	NO	(0)	NO	n.a.4	n.a.4	n.a.4	n.a.4	n.a.4	n.a.4
				Fitting uncertainty	(-)	YES	(0)	YES	n.a.4	n.a.4	n.a.4	n.a.4	n.a.4	n.a.4
			without σ_3	Mean	(-)	YES	(-)	NO	YES	NO	n.a.8	(-)	unknown	>>
				Fitting uncertainty	(-)	NO	(0)	NO	n.a.4	n.a.4	n.a.4	n.a.4	n.a.4	n.a.4
		τ_3	with σ_3	Mean	(0)	NO	(0)	NO	n.a.4	n.a.4	n.a.4	n.a.4	n.a.4	n.a.4
				Fitting uncertainty	(0)	NO	(0)	NO	n.a.4	n.a.4	n.a.4	n.a.4	n.a.4	n.a.4
			without σ_3	Mean	(-)	NO	(+)	NO	YES	YES	MAX	(+)	(-)	<<
				Fitting uncertainty	(0)	NO	(-)	YES	YES	YES	MIN	(-)	(+)	(=)
		$I_1 + I_3$	with σ_3	Mean	(0)	NO	(0)	NO	n.a.4	n.a.4	n.a.4	n.a.4	n.a.4	n.a.4
				Fitting uncertainty	(0)	NO	(0)	NO	n.a.4	n.a.4	n.a.4	n.a.4	n.a.4	n.a.4
			without σ_3	Mean	(0)	NO	(0)	NO	n.a.4	n.a.4	n.a.4	n.a.4	n.a.4	n.a.4
				Fitting uncertainty	(0)	NO	(0)	NO	n.a.4	n.a.4	n.a.4	n.a.4	n.a.4	n.a.4
		I_2	with σ_3	Mean	(0)	NO	(0)	NO	n.a.4	n.a.4	n.a.4	n.a.4	n.a.4	n.a.4
				Fitting uncertainty	(0)	NO	(0)	NO	n.a.4	n.a.4	n.a.4	n.a.4	n.a.4	n.a.4
			without σ_3	Mean	(0)	NO	(0)	NO	n.a.4	n.a.4	n.a.4	n.a.4	n.a.4	n.a.4
				Fitting uncertainty	(0)	NO	(0)	NO	n.a.4	n.a.4	n.a.4	n.a.4	n.a.4	n.a.4
		σ_3		Mean	(0)	NO	(+)	NO	YES	YES	MAX	(+)	(-)	(=)
				Standard uncertainty	(0)	NO	(0)	NO	n.a.4	n.a.4	n.a.4	n.a.4	n.a.4	n.a.4

Where n.a.4 means no conclusion can be drawn for the quadratic effect of a given factor as it is zero; n.a.8 means no clear evidence for the existence of an extreme; τ_2 is the intermediate lifetime (free positron annihilation); τ_3 is the average lifetime of ortho-positronium annihilation; $I_1 + I_3$ are intensities of the positron component associated with the free space; I_2 is the intensity of the positron component associated with the crystal space; σ_3 is the spectrum dispersion

Table S2. Symbolic interpretation of the linear effect of the SBM process (A) and rPET content (B) on the microscopic features of the bottle relative to the preform shown in Figures 1b, and 2b—symbolic interpretation of linear two-way interactions from the graph presented in Figures 1b, and 2b necessary for the interpretation of the linear main effects presented in Figures 1b, and 2b was based on the Table S2 describing the interpretation of the two-factor cross-effects presented in the first part of the article (part I [17]), where (a) is mean, (b) is fitting uncertainty, (c) is standard uncertainty.

Study	Figure	Feature		Linear effects of the SBM process (A) and RPET content (B)																						
				Linear two-way interactions							Linear main effects with respect to linear two-way interactions															
				AxB							A (SBM process—FORM)							B (RPET content)								
				A (SBM process)	B (RPET content)	AxB	|A| vs. |B|	|AxB| vs. |A|	|AxB| vs. |B|	The power of the test > 0.8	Quantitative independence from factor B	Trend of change for a low level of Factor B: (B-)	Trend of change for a high level of Factor B: (B +)	Relative absolute value of the trends for low and high levels of Factor B: |B-| vs. |B+|	Qualitative independence from Factors B	Qualitative main effect of Factor A	Power of the test > 0.8	Quantitative independence from Factor A	Trend of change for a low level of Factor A: (A-)	Trend of change for a high level of Factor A: (A +)	Relative absolute value of the trends for low and high levels of Factor A: |A-| vs. |A+|	Qualitative independence from Factors A	Qualitative main effect of Factor B	Power of the test > 0.8	Change of impact on the feature of RPER content from the power of LAMPS (Table 1(e) in part I)	Change of impact on the feature of RPER content from the power of FANS (Table 1(f) in part I)
Physical and thermal properties	1b	ρ	ALL	(+)	(+)	(-)	>	<	=	YES	NO	(+)	(+)	>	YES	(+)	YES	NO	(+)	0	n.a.2	YES	(+)	YES	+	-
			RPET	(+)	(+)	(-)	>	<	=	YES	NO	(+)	(+)	>	YES	(+)	YES	NO	(+)	0	n.a.2	YES	(+)	NO	n.a.7	n.a.7
		X_C	ALL	(+)	(+)	(-)	>	<	=	YES	NO	(+)	(+)	>	YES	(+)	YES	NO	(+)	0	n.a.2	YES	(+)	NO	0	0
			RPET	(+)	(+)	(+)	>	<	<	YES	NO	(+)	(+)	<	YES	(+)	YES	NO	(+)	(+)	<	YES	(+)	NO	n.a.7	n.a.7
		M.E.	ALL	(+)	(-)	(+)	>	<	>	YES	NO	(+)	(+)	<	YES	(+)	YES	NO	(-)	(+)	>	NO	n.a.3	YES	-	+
			RPET	(+)	0	(+)	>	<	>	YES	NO	(+)	(+)	<	YES	(+)	YES	NO	(-)	(+)	=	NO	n.a.3	NO	n.a.7	n.a.7

		η	ALL			(+)	0	(-)	>	<	>	YES	NO	(+)	(+)	>	YES	(+)	YES	NO	(+)	(-)	=	NO	n.a.3	YES	0	0
			RPET			(+)	(-)	(-)	=	<	<	YES	NO	(+)	(+)	>	YES	(+)	YES	NO	(-)	(-)	<	YES	(-)	YES	n.a.7	n.a.7
		T_g	ALL			0	0	0	=	=	=	YES	YES	0	0	n.a.2	YES	0	YES	YES	0	0	n.a.2	YES	0	YES	0	0
			RPET			0	0	0	=	=	=	NO	YES	0	0	n.a.2	YES	0	NO	YES	0	0	n.a.2	YES	0	NO	n.a.7	n.a.7
		T_m	ALL			0	0	0	=	=	=	YES	YES	0	0	n.a.2	YES	0	NO	YES	0	0	n.a.2	YES	0	NO	0	0
			RPET			0	0	0	=	=	=	NO	YES	0	0	n.a.2	YES	0	NO	YES	0	0	n.a.2	YES	0	NO	n.a.7	n.a.7
PALS analysis	2b	τ_2	ALL	with σ_3	(a)	(-)	0	0	>	<	=	YES	NO	(-)	(-)	=	YES	(-)	YES	NO	0	0	=	NO	n.a.3	NO	+	+
					(b)	(+)	(-)	(+)	>	<	<	YES	NO	(+)	(+)	<	YES	(+)	YES	NO	(-)	(-)	>	YES	(-)	YES	0	+
				without σ_3	(a)	(-)	0	(+)	>	<	>	YES	NO	(-)	(-)	>	YES	(-)	YES	NO	(-)	(+)	=	NO	n.a.3	NO	-	+
					(b)	(-)	(-)	(+)	<	=	<	YES	NO	(-)	0	n.a.2	YES	(-)	YES	NO	(-)	(-)	>	YES	(-)	YES	0	0
			RPET	with σ_3	(a)	(-)	0	(+)	>	<	>	YES	NO	(-)	(-)	>	YES	(-)	YES	NO	(-)	(+)	=	NO	n.a.3	NO	n.a.7	n.a.7
					(b)	(+)	(-)	(+)	>	<	<	YES	NO	(+)	(+)	<	YES	(+)	YES	NO	(-)	(-)	>	YES	(-)	YES	n.a.7	n.a.7
				without σ_3	(a)	(-)	0	(+)	>	<	>	YES	NO	(-)	(-)	>	YES	(-)	YES	NO	(-)	(+)	=	NO	n.a.3	NO	n.a.7	n.a.7
					(b)	0	0	(+)	=	>	>	NO	NO	(-)	(+)	=	NO	n.a.3	NO	NO	(-)	(+)	=	NO	n.a.3	NO	n.a.7	n.a.7
		τ_3	ALL	with σ_3	(a)	(-)	0	0	>	<	=	YES	YES	(-)	(-)	(=)	YES	(-)	YES	YES	0	0	n.a.2	YES	0	YES	0	0
					(b)	(+)	0	(-)	>	<	>	YES	NO	(+)	(+)	>	YES	(+)	YES	NO	(+)	(-)	=	NO	n.a.3	YES	-	+
				without σ_3	(a)	(-)	0	(+)	>	<	>	YES	NO	(-)	(-)	>	YES	(-)	YES	NO	(-)	(+)	=	NO	n.a.3	YES	-	+
					(b)	0	0	(-)	=	>	>	YES	NO	(+)	(-)	=	NO	n.a.3	YES	NO	(+)	(-)	=	NO	n.a.3	NO	-	+
			RPET	with σ_3	(a)	0	0	0	=	=	=	NO	YES	0	0	n.a.2	YES	0	NO	YES	0	0	n.a.2	YES	0	NO	n.a.7	n.a.7
					(b)	(+)	0	(-)	>	<	>	YES	NO	(+)	(+)	>	YES	(+)	YES	YES	(+)	(-)	=	NO	n.a.3	NO	n.a.7	n.a.7
				without σ_3	(a)	(-)	(-)	(-)	>	<	<	YES	NO	(-)	(-)	<	YES	(-)	YES	NO	(-)	(-)	<	YES	(-)	NO	n.a.7	n.a.7
					(b)	(+)	0	0	>	<	=	NO	YES	(+)	(+)	(=)	YES	(+)	YES	YES	0	0	n.a.2	YES	0	NO	n.a.7	n.a.7
		$I_1 + I_3$	ALL	with σ_3	(a)	(-)	0	(+)	>	<	>	YES	NO	(-)	(-)	>	YES	(-)	YES	NO	(-)	(+)	=	NO	n.a.3	NO	-	-
					(b)	(+)	0	(+)	>	<	>	YES	NO	(+)	(+)	<	YES	(+)	YES	NO	(-)	(+)	=	NO	n.a.3	YES	+	+
				without σ_3	(a)	(-)	0	(-)	>	<	>	NO	NO	(-)	(-)	<	YES	(-)	NO	NO	(+)	(-)	=	NO	n.a.3	NO	-	0
					(b)	0	0	0	=	=	=	YES	YES	0	0	n.a.2	YES	0	NO	YES	0	0	n.a.2	YES	0	YES	0	0
			RPET	with σ_3	(a)	(-)	0	(-)	>	<	>	YES	NO	(-)	(-)	<	YES	(-)	YES	NO	(+)	(-)	=	NO	n.a.3	NO	n.a.7	n.a.7
					(b)	(+)	0	(-)	>	<	>	YES	NO	(+)	(+)	>	YES	(+)	YES	NO	(+)	(-)	=	NO	n.a.3	NO	n.a.7	n.a.7
				wit ho	(a)	(-)	0	(-)	>	<	>	YES	NO	(-)	(-)	<	YES	(-)	YES	NO	(+)	(-)	=	NO	n.a.3	NO	n.a.7	n.a.7

					(b)	0	0	0	=	=	=	NO	YES	0	0	n.a.2	YES	0	NO	YES	0	0	n.a.2	YES	0	NO	n.a.7	n.a.7
		I_2	ALL	with σ_3	(a)	(+)	(-)	(-)	>	<	<	YES	NO	(+)	(+)	>	YES	(+)	YES	NO	(-)	(-)	<	YES	(-)	NO	+	0
					(b)	(+)	0	(+)	>	<	>	YES	NO	(+)	(+)	<	YES	(+)	YES	NO	(-)	(+)	=	NO	n.a.3	YES	+	+
				without σ_3	(a)	(+)	0	(+)	>	<	>	NO	NO	(+)	(+)	<	YES	(+)	NO	NO	(-)	(+)	=	NO	n.a.3	NO	+	0
					(b)	0	0	0	=	=	=	YES	YES	0	0	n.a.2	YES	0	NO	YES	0	0	n.a.2	YES	0	YES	0	0
			RPET	with σ_3	(a)	(+)	0	(+)	>	<	>	YES	NO	(+)	(+)	<	YES	(+)	YES	NO	(-)	(+)	=	NO	n.a.3	NO	n.a.7	n.a.7
					(b)	(+)	0	(-)	>	<	>	YES	NO	(+)	(+)	>	YES	(+)	YES	NO	(+)	(-)	=	NO	n.a.3	NO	n.a.7	n.a.7
				without σ_3	(a)	(+)	(+)	(+)	>	<	>	YES	NO	(+)	(+)	<	YES	(+)	YES	NO	(-)	(+)	<	NO	n.a.3	NO	n.a.7	n.a.7
					(b)	0	0	0	=	=	=	NO	YES	0	0	n.a.2	YES	0	NO	YES	0	0	n.a.2	YES	0	NO	n.a.7	n.a.7
		σ_3	ALL		(a)	(+)	0	(-)	>	<	>	NO	NO	(+)	(+)	>	YES	(+)	NO	NO	(+)	(-)	=	NO	n.a.3	NO	-	-
					(c)	(+)	0	(-)	>	<	>	YES	NO	(+)	(+)	>	YES	(+)	YES	NO	(+)	(-)	=	NO	n.a.3	YES	-	+
			RPET		(a)	(+)	0	(+)	>	<	>	YES	NO	(+)	(+)	<	YES	(+)	YES	NO	(-)	(+)	=	NO	n.a.3	NO	n.a.7	n.a.7
					(c)	(+)	0	(-)	>	<	>	YES	NO	(+)	(+)	>	YES	(+)	YES	NO	(+)	(-)	=	NO	n.a.3	NO	n.a.7	n.a.7

Where n.a.2 means not applicable because there is at least one zero trend; n.a.3 means no conclusion can be drawn for the linear effect of a given factor for any settings of the other two factors, i.e., the effect of a given factor should be analyzed in relation to the values of the other factors due to the overlapping of both interaction effects and low statistical power for the main effect of a given factor; A.YES means almost "≈" YES; n.a.7 means no conclusion can be drawn because the lamp power and fan power settings were constant (the middle value of the lamp power variability and the fan power variability); M.E. is the microcavitation effect

Table S3. Symbolic interpretation of the quadratic influence of the SBM process (A) and rPET content (B) on the microscopic features of the bottle in relation to the preform shown in Figures 1b, and 2b—symbolic interpretation of the quadratic effects in relation to the linear effects was based on the idea presented in the figure S1 describing the interpretation of quadratic effects versus linear effects presented in the first part of the article (part I [17]), where: (a) is mean, (b) is fitting uncertainty, (c) is standard uncertainty.

Study	Figure	Feature		Quadratic main effects with respect to linear main effects of RPET content (B)										
				Linear effect			Sign of quadratic main effect	Extreme	Trend of change for low values of Factor B	Trend of change for high values of Factor B	|Trend of change for low values of factor B| vs |Trend of change [illegible]	Power of the test > 0.8	Change of impact on feature of RPER content from	
				$\|B^2\|>\|¼ B\|$	$\|B^2\|>\|½ B\|$	Sign of linear main effect							Power of LAMPS (Table 1[(e)] in [illegible]	Power of FANS (Table 1[(f)] in [illegible]
Physical and [illegible]	1b	ρ	ALL	NO	NO	(+)	(-)	n.a.5	(+)	(+)	<	NO	-	-

			RPET			n.a.4	n.a.4	(+)	0	n.a.4	n.a.4	n.a.4	n.a.4	NO	n.a.7	n.a.7
		X_C	ALL			n.a.4	n.a.4	(+)	0	n.a.4	n.a.4	n.a.4	n.a.4	NO	0	0
		X_C	RPET			n.a.4	n.a.4	(+)	0	n.a.4	n.a.4	n.a.4	n.a.4	NO	n.a.7	n.a.7
		M.E.	ALL			n.a.4	n.a.4	(-)	0	n.a.4	n.a.4	n.a.4	n.a.4	NO	+	+
		M.E.	RPET			n.a.4	n.a.4	0	0	n.a.4	n.a.4	n.a.4	n.a.4	NO	n.a.7	n.a.7
		η	ALL			n.a.4	n.a.4	0	0	n.a.4	n.a.4	n.a.4	n.a.4	YES	0	0
		η	RPET			YES	NO	(-)	(-)	n.a.8	(-)	unknown	>>	NO	n.a.7	n.a.7
		T_g	ALL			n.a.4	n.a.4	0	0	n.a.4	n.a.4	n.a.4	n.a.4	NO	0	0
		T_g	RPET			n.a.4	n.a.4	0	0	n.a.4	n.a.4	n.a.4	n.a.4	NO	n.a.7	n.a.7
		T_m	ALL			n.a.4	n.a.4	0	0	n.a.4	n.a.4	n.a.4	n.a.4	YES	0	0
		T_m	RPET			n.a.4	n.a.4	0	0	n.a.4	n.a.4	n.a.4	n.a.4	NO	n.a.7	n.a.7
PALS analysis	2b	τ_2	ALL	with σ_3	(a)	n.a.4	n.a.4	0	0	n.a.4	n.a.4	n.a.4	n.a.4	NO	+	+
PALS analysis	2b	τ_2	ALL	with σ_3	(b)	n.a.4	n.a.4	(-)	0	n.a.4	n.a.4	n.a.4	n.a.4	NO	0	-
PALS analysis	2b	τ_2	ALL	without σ_3	(a)	n.a.4	n.a.4	0	0	n.a.4	n.a.4	n.a.4	n.a.4	NO	-	+
PALS analysis	2b	τ_2	ALL	without σ_3	(b)	YES	YES	(-)	(-)	MIN	(-)	(+)	>>	YES	0	0
PALS analysis	2b	τ_2	RPET	with σ_3	(a)	n.a.4	n.a.4	0	0	n.a.4	n.a.4	n.a.4	n.a.4	NO	n.a.7	n.a.7
PALS analysis	2b	τ_2	RPET	with σ_3	(b)	n.a.4	n.a.4	(-)	0	n.a.4	n.a.4	n.a.4	n.a.4	NO	n.a.7	n.a.7
PALS analysis	2b	τ_2	RPET	without σ_3	(a)	n.a.4	n.a.4	0	0	n.a.4	n.a.4	n.a.4	n.a.4	NO	n.a.7	n.a.7
PALS analysis	2b	τ_2	RPET	without σ_3	(b)	YES	YES	0	(-)	MIN	(-)	(+)	=	NO	n.a.7	n.a.7
PALS analysis	2b	τ_3	ALL	with σ_3	(a)	n.a.4	n.a.4	0	0	n.a.4	n.a.4	n.a.4	n.a.4	NO	0	0
PALS analysis	2b	τ_3	ALL	with σ_3	(b)	n.a.4	n.a.4	0	0	n.a.4	n.a.4	n.a.4	n.a.4	NO	-	-
PALS analysis	2b	τ_3	ALL	without σ_3	(a)	YES	YES	0	(+)	MAX	(+)	(-)	=	YES	+	-
PALS analysis	2b	τ_3	ALL	without σ_3	(b)	n.a.4	n.a.4	0	0	n.a.4	n.a.4	n.a.4	n.a.4	YES	+	+
PALS analysis	2b	τ_3	RPET	with σ_3	(a)	n.a.4	n.a.4	0	0	n.a.4	n.a.4	n.a.4	n.a.4	NO	n.a.7	n.a.7
PALS analysis	2b	τ_3	RPET	with σ_3	(b)	n.a.4	n.a.4	0	0	n.a.4	n.a.4	n.a.4	n.a.4	NO	n.a.7	n.a.7
PALS analysis	2b	τ_3	RPET	without σ_3	(a)	n.a.4	n.a.4	(-)	0	n.a.4	n.a.4	n.a.4	n.a.4	NO	n.a.7	n.a.7
PALS analysis	2b	τ_3	RPET	without σ_3	(b)	YES	YES	0	(-)	MIN	(-)	(+)	=	YES	n.a.7	n.a.7
PALS analysis	2b	$I_1 + I_3$	ALL	with σ_3	(a)	n.a.4	n.a.4	0	0	n.a.4	n.a.4	n.a.4	n.a.4	NO	-	0
PALS analysis	2b	$I_1 + I_3$	ALL	with σ_3	(b)	n.a.4	n.a.4	0	0	n.a.4	n.a.4	n.a.4	n.a.4	NO	+	+

					withou t σ3	(a)	n.a.4	n.a.4	0	0	n.a.4	n.a.4	n.a.4	n.a.4	NO	0	0
						(b)	n.a.4	n.a.4	0	0	n.a.4	n.a.4	n.a.4	n.a.4	YES	0	0
				RPET	with σ3	(a)	n.a.4	n.a.4	0	0	n.a.4	n.a.4	n.a.4	n.a.4	NO	n.a.7	n.a.7
						(b)	n.a.4	n.a.4	0	0	n.a.4	n.a.4	n.a.4	n.a.4	NO	n.a.7	n.a.7
					withou t σ3	(a)	n.a.4	n.a.4	0	0	n.a.4	n.a.4	n.a.4	n.a.4	NO	n.a.7	n.a.7
						(b)	n.a.4	n.a.4	0	0	n.a.4	n.a.4	n.a.4	n.a.4	NO	n.a.7	n.a.7
			I_2	ALL	with σ3	(a)	n.a.4	n.a.4	(-)	0	n.a.4	n.a.4	n.a.4	n.a.4	NO	+	0
						(b)	n.a.4	n.a.4	0	0	n.a.4	n.a.4	n.a.4	n.a.4	NO	+	+
					withou t σ3	(a)	n.a.4	n.a.4	0	0	n.a.4	n.a.4	n.a.4	n.a.4	NO	0	0
						(b)	n.a.4	n.a.4	0	0	n.a.4	n.a.4	n.a.4	n.a.4	YES	0	0
				RPET	with σ3	(a)	n.a.4	n.a.4	0	0	n.a.4	n.a.4	n.a.4	n.a.4	NO	n.a.7	n.a.7
						(b)	n.a.4	n.a.4	0	0	n.a.4	n.a.4	n.a.4	n.a.4	NO	n.a.7	n.a.7
					withou t σ3	(a)	n.a.4	n.a.4	(+)	0	n.a.4	n.a.4	n.a.4	n.a.4	NO	n.a.7	n.a.7
						(b)	n.a.4	n.a.4	0	0	n.a.4	n.a.4	n.a.4	n.a.4	NO	n.a.7	n.a.7
			σ_3	ALL		(a)	YES	YES	0	(+)	MAX	(+)	(-)	=	NO	-	-
						(c)	n.a.4	n.a.4	0	0	n.a.4	n.a.4	n.a.4	n.a.4	NO	-	-
				RPET		(a)	n.a.4	n.a.4	0	0	n.a.4	n.a.4	n.a.4	n.a.4	NO	0	0
						(c)	n.a.4	n.a.4	0	0	n.a.4	n.a.4	n.a.4	n.a.4	NO	0	0

Where n.a.4 means no conclusion can be drawn for the quadratic effect of a given factor as it is zero; n.a.5 means no extreme because the quadratic effect is smaller than ¼ of the linear effect; n.a.8 means no clear evidence for the existence of an extreme; M.E. is the microcavitation effect;

Table S4. Symbolic interpretation of the linear two-way interactions from the graph presented in Figures 1c, 2c, and 3 of the influence of rPET content (A), the power of heating lamps (B), and the power of cooling fans (C) on the microscopic features of the bottle, necessary to interpret the linear main effects presented in Table S5 based on the table S2 describing the interpretation of the two-factor cross-effects presented in the first part of the article (part I [17]), where (a) is mean, (b) is fitting uncertainty, (c) is standard uncertainty.

Study	Figure	Feature	Linear two-way interactions																				
			AxB							AxC							BxC						
			A (PRE	B (pow	AxB	|A|v s.|B|	|AxB |vs.|	|AxB |vs.|	Powe r of	A (PRE	C (pow	AxC	|A|v s.|C|	|Ax C|vs.	|Ax C|vs.	Powe r of	B (pow	C (pow	BxC	|B|v	|BxC |vs.|	|BxC |vs.|	Powe r of

Physical and thermal properties	1c	ρ			(-)	(+)	(+)	<	>	<	YES	(-)	(-)	(+)	<	=	<	NO	(+)	(-)	(+)	>	<	>	YES
		X_C			0	0	0	=	=	=	NO	0	0	0	=	=	=	NO	0	0	0	=	=	=	NO
		M.E.			0	0	0	=	=	=	NO	0	0	0	=	=	=	NO	0	0	0	=	=	=	NO
		η			(-)	0	0	>	<	=	YES	(-)	0	(+)	>	<	>	YES	0	0	0	=	=	=	NO
		T_g			0	(+)	0	<	=	<	NO	0	0	0	=	=	=	NO	(+)	0	(-)	>	=	>	YES
		T_m			0	0	0	=	=	=	NO	0	0	0	=	=	=	NO	0	0	0	=	=	=	YES
PALS analysis	2c	τ_2	with σ_3	(a)	0	0	0	=	=	=	NO	0	0	0	=	=	=	YES	0	0	0	=	=	=	NO
				(b)	(-)	(-)	(-)	>	<	=	YES	(-)	0	(-)	>	>	>	YES	(-)	0	0	>	<	=	NO
			without σ_3	(a)	0	0	0	=	=	=	YES	0	0	0	=	=	=	YES	0	0	0	=	=	=	NO
				(b)	0	0	0	=	=	=	YES	0	0	0	=	=	=	NO	0	0	0	=	=	=	YES
		τ_3	with σ_3	(a)	(-)	0	0	>	<	=	NO	(-)	(+)	(-)	<	>	=	YES	0	(+)	0	<	=	<	NO
				(b)	(-)	0	0	>	<	=	YES	(-)	0	(-)	>	<	>	YES	0	0	0	=	=	=	NO
			without σ_3	(a)	(-)	0	(+)	>	=	>	YES	(-)	(+)	(-)	<	>	>	YES	0	(+)	(-)	<	>	>	YES
				(b)	(-)	0	0	>	<	=	NO	(-)	0	0	>	<	=	NO	0	0	0	=	=	=	YES
		$I_1 + I_3$	with σ_3	(a)	0	0	0	=	=	=	NO	0	(-)	(+)	<	>	<	YES	0	(-)	0	<	=	<	MO
				(b)	0	0	0	=	=	=	YES	0	0	(+)	=	>	>	YES	0	0	0	=	=	=	YES
			without σ_3	(a)	0	(-)	0	<	=	<	NO	0	0	0	=	=	=	YES	(-)	0	0	>	<	=	YES
				(b)	(-)	(-)	0	>	<	<	NO	(-)	0	0	>	<	=	NO	(-)	0	0	>	<	=	YES
		I_2	with σ_3	(a)	0	0	0	=	=	=	NO	0	(+)	(-)	<	>	=	YES	0	(+)	0	<	=	<	NO
				(b)	0	0	0	=	=	=	YES	0	0	(-)	=	>	>	YES	0	0	0	=	=	=	YES
			without σ_3	(a)	0	(+)	0	<	=	<	NO	0	0	0	=	=	=	YES	(+)	0	0	>	<	=	YES
				(b)	(-)	(-)	0	>	<	<	NO	(-)	0	0	>	<	=	NO	(-)	0	0	>	<	=	YES
		σ_3		(a)	0	0	0	=	=	=	YES	0	(-)	(+)	<	>	=	YES	0	(-)	(-)	<	>	<	YES
				(c)	(-)	0	0	>	<	=	YES	(-)	(+)	(-)	>	<	=	YES	0	(+)	0	<	=	<	NO
Macroscopic properties	3	b.p.			-	-	0	<	<	<	NO	-	+	0	>	<	<	NO	-	+	0	>	<	<	NO
		TH-I			-	-	0	<	<	<	YES	-	+	0	<	<	<	NO	-	+	0	>	<	<	NO
		TH-II			0	-	-	<	>	<	YES	0	+	0	<	=	<	NO	-	+	0	=	<	<	NO
		TH-III			-	+	+	>	<	>	YES	-	-	0	>	<	<	NO	+	-	+	>	>	>	YES

Table S5. Symbolic interpretation of the linear main effects from the graphs presented in Figures 1c, 2c, and 3 in relation to the linear two-way effects presented in Table S4 and the table S2 describing the interpretation of the two-way cross effects of the influence of rPET (A) content, the power of heating lamps (B), and the power of cooling fans (C) on the microscopic features of the bottle, where (a) is mean, (b) is fitting uncertainty, (c) is standard uncertainty.

Study	Figure	Feature	Linear main effects with respect to linear two-way interactions																																
			A (RPET content)											B (power of LAMPS)											C (power of FANS)										
			AxB				AxC							AxB				BxC							AxC				BxC						
			Quantitative independence from Factor B	Trend of change for a low level of Factor B: (B-)	Trend of change for a high level of Factor B: (B +)	Relative absolute value of the trends for low and high levels of Factor B: |B-| vs. |B+|	Quantitative independence from Factor C	Trend of change for a low level of Factor C: (C-)	Trend of change for a high level of Factor C: (C +)	Relative absolute value of the trends for low and high levels of Factor C: |C-| vs. |C+|	Qualitative independence from Factors B and C	Qualitative main effect of Factor A	Power of the test > 0.8	Quantitative independence from Factor A	Trend of change for a low level of Factor A: (A-)	Trend of change for a high level of Factor A: (A +)	Relative absolute value of the trends for low and high levels of Factor A: |A-| vs. |A+|	Quantitative independence from Factor C	Trend of change for a low level of Factor C: (C-)	Trend of change for a high level of Factor C: (C +)	Relative absolute value of the trends for low and high levels of Factor C: |C-| vs. |C+|	Qualitative independence from Factors A and C	Qualitative main effect of Factor B	Power of the test > 0.8	Quantitative independence from Factor A	Trend of change for a low level of Factor A: (A-)	Trend of change for a high level of Factor A: (A +)	Relative absolute value of the trends for low and high levels of Factor A: |A-| vs. |A+|	Quantitative independence from Factor B	Trend of change for a low level of Factor B: (B-)	Trend of change for a high level of Factor B: (B +)	Relative absolute value of the trends for low and high levels of Factor B: |B-| vs. |B+|	Qualitative independence from Factors A and B	Qualitative main effect of Factor C	Power of the test > 0.8
Physical and thermal properties	1c	ρ	NO	(-)	(+)	>	NO	(-)	0	n.a.2	NO	n.a.3	NO	NO	(+)	(+)	<	NO	(+)	(+)	>	NO	n.a.3	YES	NO	(-)	(-)	>	NO	(-)	(+)	<	NO	n.a.3	YES
		X_C	YES	0	0	(=)	YES	0	0	(=)	YES	0	NO	YES	0	0	(=)	YES	0	0	(=)	YES	0	NO	YES	0	0	(=)	YES	0	0	(=)	YES	0	NO
		M.E.	YES	0	0	(=)	YES	0	0	(=)	YES	0	NO	YES	0	0	(=)	YES	0	0	(=)	YES	0	NO	YES	0	0	(=)	YES	0	0	(=)	YES	0	NO
		η	YES	(-)	(-)	(=)	NO	(-)	(-)	(>)	YES	(-)	YES	YES	0	0	(=)	YES	0	0	(=)	YES	0	YES	NO	(-)	(+)	(=)	YES	0	0	(=)	NO	n.a.3	NO
		T_g	YES	0	0	(=)	YES	0	0	(=)	YES	0	NO	YES	(+)	(+)	(=)	NO	(+)	0	n.a.2	YES	(+)	YES	YES	0	0	(=)	NO	(+)	(-)	(=)	NO	n.a.3	YES
		T_m	YES	0	0	(=)	YES	0	0	(=)	YES	0	NO	YES	0	0	(=)	YES	0	0	(=)	YES	0	YES	YES	0	0	(=)	YES	0	0	(=)	YES	0	NO
PALS analysis	2c	τ_2 with σ_3 (a)	YES	0	0	(=)	YES	0	0	(=)	YES	0	NO	YES	0	0	(=)	YES	0	0	(=)	YES	0	NO	YES	0	0	(=)	YES	0	0	(=)	YES	0	NO
		τ_2 with σ_3 (b)	NO	(-)	(-)	(<)	NO	(+)	(-)	(<)	A.YES	≈ (-)	YES	NO	0	(-)	n.a.2	NO	(-)	(-)	(=)	YES	(-)	YES	NO	(+)	(-)	(=)	YES	0	0	(=)	NO	n.a.3	YES
		τ_2 without σ_3 (a)	YES	0	0	(=)	YES	0	0	(=)	YES	0	NO	YES	0	0	(=)	YES	0	0	(=)	YES	0	NO	YES	0	0	(=)	YES	0	0	(=)	YES	0	YES
		τ_2 without σ_3 (b)	YES	0	0	(=)	YES	0	0	(=)	YES	0	YES	YES	0	0	(=)	YES	0	0	(=)	YES	0	YES	YES	0	0	(=)	YES	0	0	(=)	YES	0	NO
		τ_3 with σ_3 (a)	YES	(-)	(-)	(=)	NO	(+)	(-)	(<)	NO	n.a.3	NO	YES	0	0	(=)	YES	0	0	(=)	YES	0	NO	NO	(+)	0	n.a.2	YES	(+)	(+)	(=)	NO	n.a.3	YES

				(b)	YES	(-)	(-)	(=)	NO	(-)	(-)	(<)	YES	(-)	YES	YES	0	0	(=)	YES	0	0	(=)	YES	0	YES	NO	(+)	(-)	(=)	YES	0	0	(=)	NO	n.a.3	YES
			without σ_3	(a)	NO	(-)	0	n.a.2	NO	(+)	(-)	(<)	NO	n.a.3	NO	NO	(-)	(+)	(=)	NO	(+)	(-)	(=)	NO	n.a.3	NO	NO	(+)	(-)	(>)	NO	(+)	(-)	(>)	NO	n.a.3	YES
				(b)	YES	(-)	(-)	(=)	YES	(-)	(-)	(=)	YES	(-)	YES	YES	0	0	(=)	YES	0	0	(=)	YES	0	YES	YES	0	0	(=)	YES	0	0	(=)	YES	0	YES
		$I_1 + I_3$	with σ_3	(a)	YES	0	0	(=)	NO	(-)	(+)	(=)	NO	n.a.3	YES	YES	0	0	(=)	YES	0	0	(=)	YES	0	YES	NO	(-)	(+)	(>)	YES	(-)	(-)	(=)	YES	(-)	YES
				(b)	YES	0	0	(=)	NO	(-)	(+)	(=)	NO	n.a.3	YES	YES	0	0	(=)	YES	0	0	(=)	YES	0	NO	NO	(-)	(+)	(=)	YES	0	0	(=)	NO	n.a.3	NO
			without σ_3	(a)	YES	0	0	(=)	YES	0	0	(=)	YES	0	NO	YES	(-)	(-)	(=)	YES	(-)	(-)	(=)	YES	(-)	YES	YES	0	0	(=)	YES	0	0	(=)	YES	0	YES
				(b)	YES	(-)	(-)	(=)	YES	(-)	(-)	(=)	YES	(-)	YES	YES	(-)	(-)	(=)	YES	(-)	(-)	(=)	YES	(-)	YES	YES	0	0	(=)	YES	0	0	(=)	YES	0	YES
		I_2	with σ_3	(a)	YES	0	0	(=)	NO	(+)	(-)	(=)	NO	n.a.3	YES	YES	0	0	(=)	YES	0	0	(=)	YES	0	YES	NO	(+)	0	n.a.2	YES	(+)	(+)	(=)	YES	(+)	YES
				(b)	YES	0	0	(=)	NO	(+)	(-)	(=)	NO	n.a.3	YES	YES	0	0	(=)	YES	0	0	(=)	YES	0	NO	NO	(+)	(-)	(=)	YES	0	0	(=)	NO	n.a.3	NO
			without σ_3	(a)	YES	0	0	(=)	YES	0	0	(=)	YES	0	NO	YES	(+)	(+)	(=)	YES	(+)	(+)	(=)	YES	(+)	YES	YES	0	0	(=)	YES	0	0	(=)	YES	0	YES
				(b)	YES	(-)	(-)	(=)	YES	(-)	(-)	(=)	YES	(-)	YES	YES	(-)	(-)	(=)	YES	(-)	(-)	(=)	YES	(-)	YES	YES	0	0	(=)	YES	0	0	(=)	YES	0	YES
		σ_3		(a)	YES	0	0	(=)	NO	(-)	(+)	(=)	NO	n.a.3	NO	YES	0	0	(=)	NO	(+)	(-)	(=)	NO	n.a.3	NO	NO	(-)	0	n.a.2	NO	(-)	(-)	(<)	YES	(-)	YES
				(c)	YES	(-)	(-)	(=)	NO	(-)	(-)	(<)	YES	(-)	YES	YES	0	0	(=)	YES	0	0	(=)	YES	0	YES	NO	(+)	0	n.a.2	YES	(+)	(+)	(=)	YES	(+)	YES
Macroscopic properties	3	b.p.			YES	(-)	(-)	(=)	YES	(-)	(-)	(=)	YES	(-)	YES	YES	(-)	(-)	(=)	YES	(-)	(-)	(=)	YES	(-)	YES	YES	(+)	(+)	(=)	YES	(+)	(+)	(=)	YES	(+)	YES
		TH-I			YES	(-)	(-)	(=)	YES	(-)	(-)	(=)	YES	(-)	YES	YES	(-)	(-)	(=)	YES	(-)	(-)	(=)	YES	(-)	YES	YES	(+)	(+)	(=)	YES	(+)	(+)	(=)	YES	(+)	YES
		TH-II			NO	(+)	(-)	(=)	YES	(0)	(0)	(=)	NO	n.a.3	NO	NO	(-)	(-)	<	YES	(-)	(-)	(=)	YES	(-)	YES	YES	(+)	(+)	(=)	YES	(+)	(+)	(=)	YES	(+)	YES
		TH-III			NO	(-)	(-)	(>)	YES	(-)	(-)	(=)	YES	(-)	YES	NO	(-)	(+)	(<)	NO	(-)	(+)	(<)	A.YES	≈ (+)	YES	YES	(-)	(-)	(=)	NO	(-)	(+)	(>)	A.YES	≈ (-)	YES

Where n.a.2 means not applicable because there is at least one zero trend; n.a.3 means no conclusion can be drawn for the linear effect of a given factor for any settings of the other two factors, i.e., the effect of a given factor should be analyzed in relation to the values of the other factors due to the overlapping of both interaction effects and low statistical power for the main effect of a given factor; A.YES means almost "≈" YES; M.E. is the microcavitation effect

Table S6. Symbolic interpretation of the quadratic main effects from the graphs shown in Figures 1c, 2c, and 3 of the influence of rPET content (A), the power of heating lamps (B), and the power of cooling fans (C) on the microscopic features of the bottle in relation to the linear main effects presented in Tables S.4 and S.5 based on the idea presented in the figures S1 and S2 describing the interpretation of quadratic effects in relation to the linear effects presented in the first part of the article [17], where (a) is mean, (b) is fitting uncertainty, (c) is standard uncertainty.

Study	Figure	Feature	Quadratic main effects with respect to linear main effects																															
			A^2 (RPET content)										B^2 (power of LAMPS)										C^2 (power of FANS)									
			Does the quadratic effect occur?	Linear effect			Sign of quadratic main effect	Extreme	Trend of change for low values of Factor A	Trend of change for high values of Factor A	|Trend of change for low values of Factor A| vs. |Trend of change for high values of Factor A|	Power of the test > 0.8	Does the quadratic effect occur?	Linear effect			Sign of quadratic main effect	Extreme	Trend of change for low values of Factor B	Trend of change for high values of Factor B	|Trend of change for low values of Factor B| vs. |Trend of change for high values of Factor B|	Power of the test > 0.8	Does the quadratic effect occur?	Linear effect			Sign of quadratic main effect	Extreme	Trend of change for low values of Factor C	Trend of change for high values of Factor C	|Trend of change for low values of Factor C| vs. |Trend of change for high values of Factor C|	Power of the test > 0.8
				$\|A^2\|>\|\frac{1}{4}A\|$	$\|A^2\|>\|\frac{1}{2}A\|$	Sign of linear main effect								$\|B^2\|>\|\frac{1}{4}B\|$	$\|B^2\|>\|\frac{1}{2}B\|$	Sign of linear main effect								$\|C^2\|>\|\frac{1}{4}C\|$	$\|C^2\|>\|\frac{1}{2}C\|$	Sign of linear main effect						
Physical and thermal properties	1c	ρ	YES	YES	YES	(-)	(+)	MAX	(+)	(-)	<<	YES	YES	NO	NO	(+)	(+)	n.a.5	(+)	(+)	>	YES	YES	YES	YES	(-)	(+)	MAX	(+)	(-)	<<	YES
		X_C	NO	n.a.4	n.a.4	0	0	n.a.4	n.a.4	n.a.4	n.a.4	NO	NO	n.a.4	n.a.4	0	0	n.a.4	n.a.4	n.a.4	n.a.4	NO	NO	n.a.4	n.a.4	0	0	n.a.4	n.a. 4	n.a.4	n.a.4	NO
		M.E.	NO	n.a.4	n.a.4	0	0	n.a.4	n.a.4	n.a.4	n.a.4	YES	NO	n.a.4	n.a.4	0	0	n.a.4	n.a.4	n.a.4	n.a.4	YES	YES	YES	YES	0	(-)	MIN	(-)	(+)	=	YES
		η	YES	YES	NO	(-)	(-)	n.a.8	(-)	unkn own	>>	YES	NO	n.a.4	n.a.4	0	0	n.a.4	n.a.4	n.a.4	n.a.4	NO	YES	YES	YES	0	(+)	MAX	(+)	(-)	=	YES
		T_g	NO	n.a.4	n.a.4	0	0	n.a.4	n.a.4	n.a.4	n.a.4	NO	NO	n.a.4	n.a.4	(+)	0	n.a.4	n.a.4	n.a.4	n.a.4	NO	NO	n.a.4	n.a.4	0	0	n.a.4	n.a. 4	n.a.4	n.a.4	NO

		T_m			NO	n.a.4	n.a.4	0	0	n.a.4	n.a.4	n.a.4	n.a.4	YES	NO	n.a.4	n.a.4	0	0	n.a.4	n.a.4	n.a.4	n.a.4	NO	NO	n.a.4	n.a.4	0	0	n.a.4	n.a. 4	n.a.4	n.a.4	NO
PALS analysis	2c	τ_2	with σ_3	(a)	NO	n.a.4	n.a.4	0	0	n.a.4	n.a.4	n.a.4	n.a.4	NO	NO	n.a.4	n.a.4	0	0	n.a.4	n.a.4	n.a.4	n.a.4	NO	NO	n.a.4	n.a.4	0	0	n.a.4	n.a. 4	n.a.4	n.a.4	YES
				(b)	NO	n.a.4	n.a.4	(-)	0	n.a.4	n.a.4	n.a.4	n.a.4	YES	NO	n.a.4	n.a.4	(-)	0	n.a.4	n.a.4	n.a.4	n.a.4	YES	YES	YES	YES	0	(-)	MIN	(-)	(+)	=	YES
			without σ_3	(a)	NO	n.a.4	n.a.4	0	0	n.a.4	n.a.4	n.a.4	n.a.4	NO	NO	n.a.4	n.a.4	0	0	n.a.4	n.a.4	n.a.4	n.a.4	NO	YES	YES	YES	0	(+)	MAX	(+)	(-)	=	YES
				(b)	NO	n.a.4	n.a.4	0	0	n.a.4	n.a.4	n.a.4	n.a.4	YES	NO	n.a.4	n.a.4	0	0	n.a.4	n.a.4	n.a.4	n.a.4	NO	NO	n.a.4	n.a.4	0	0	n.a.4	n.a. 4	n.a.4	n.a.4	YES
		τ_3	with σ_3	(a)	NO	n.a.4	n.a.4	(-)	0	n.a.4	n.a.4	n.a.4	n.a.4	NO	YES	YES	YES	0	(+)	MAX	(+)	(-)	=	YES	NO	n.a.4	n.a.4	(+)	0	n.a.4	n.a. 4	n.a.4	n.a.4	NO
				(b)	NO	n.a.4	n.a.4	(-)	0	n.a.4	n.a.4	n.a.4	n.a.4	NO	NO	n.a.4	n.a.4	0	0	n.a.4	n.a.4	n.a.4	n.a.4	NO	NO	n.a.4	n.a.4	0	0	n.a.4	n.a. 4	n.a.4	n.a.4	NO
			without σ_3	(a)	YES	YES	YES	(-)	(+)	MAX	(+)	(-)	<<	YES	YES	YES	YES	0	(+)	MAX	(+)	(-)	=	YES	NO	n.a.4	n.a.4	(+)	0	n.a.4	n.a. 4	n.a.4	n.a.4	NO
				(b)	NO	n.a.4	n.a.4	(-)	0	n.a.4	n.a.4	n.a.4	n.a.4	NO	NO	n.a.4	n.a.4	0	0	n.a.4	n.a.4	n.a.4	n.a.4	YES	NO	n.a.4	n.a.4	0	0	n.a.4	n.a. 4	n.a.4	n.a.4	NO
		$I_1 + I_3$	with σ_3	(a)	YES	YES	YES	0	(+)	MAX	(+)	(-)	=	YES	NO	n.a.4	n.a.4	0	0	n.a.4	n.a.4	n.a.4	n.a.4	YES	NO	n.a.4	n.a.4	(-)	0	n.a.4	n.a. 4	n.a.4	n.a.4	NO
				(b)	NO	n.a.4	n.a.4	0	0	n.a.4	n.a.4	n.a.4	n.a.4	YES	NO	n.a.4	n.a.4	0	0	n.a.4	n.a.4	n.a.4	n.a.4	NO	NO	n.a.4	n.a.4	0	0	n.a.4	n.a. 4	n.a.4	n.a.4	YES
			without σ_3	(a)	NO	n.a.4	n.a.4	0	0	n.a.4	n.a.4	n.a.4	n.a.4	YES	NO	n.a.4	n.a.4	(-)	0	n.a.4	n.a.4	n.a.4	n.a.4	NO	NO	n.a.4	n.a.4	0	0	n.a.4	n.a. 4	n.a.4	n.a.4	NO
				(b)	NO	n.a.4	n.a.4	(-)	0	n.a.4	n.a.4	n.a.4	n.a.4	NO	NO	n.a.4	n.a.4	(-)	0	n.a.4	n.a.4	n.a.4	n.a.4	YES	NO	n.a.4	n.a.4	0	0	n.a.4	n.a. 4	n.a.4	n.a.4	NO

		I_2	with σ_3 (a)	YES	YES	YES	0	(-)	MIN	(-)	(+)	=	YES	NO	n.a.4	n.a.4	0	0	n.a.4	n.a.4	n.a.4	n.a.4	YES	NO	n.a.4	n.a.4	(+)	0	n.a.4	n.a. 4	n.a.4	n.a.4	NO
			with σ_3 (b)	NO	n.a.4	n.a.4	0	0	n.a.4	n.a.4	n.a.4	n.a.4	YES	NO	n.a.4	n.a.4	0	0	n.a.4	n.a.4	n.a.4	n.a.4	NO	NO	n.a.4	n.a.4	0	0	n.a.4	n.a. 4	n.a.4	n.a.4	NO
			without σ_3 (a)	NO	n.a.4	n.a.4	0	0	n.a.4	n.a.4	n.a.4	n.a.4	YES	NO	n.a.4	n.a.4	(+)	0	n.a.4	n.a.4	n.a.4	n.a.4	NO	NO	n.a.4	n.a.4	0	0	n.a.4	n.a. 4	n.a.4	n.a.4	YES
			without σ_3 (b)	NO	n.a.4	n.a.4	(-)	0	n.a.4	n.a.4	n.a.4	n.a.4	NO	NO	n.a.4	n.a.4	(-)	0	n.a.4	n.a.4	n.a.4	n.a.4	YES	NO	n.a.4	n.a.4	0	0	n.a.4	n.a. 4	n.a.4	n.a.4	NO
		σ_3	(a)	YES	YES	YES	0	(+)	MAX	(+)	(-)	=	YES	NO	n.a.4	n.a.4	0	0	n.a.4	n.a.4	n.a.4	n.a.4	YES	NO	n.a.4	n.a.4	(-)	0	n.a.4	n.a. 4	n.a.4	n.a.4	NO
			(c)	NO	n.a.4	n.a.4	(-)	0	n.a.4	n.a.4	n.a.4	n.a.4	NO	NO	n.a.4	n.a.4	0	0	n.a.4	n.a.4	n.a.4	n.a.4	NO	NO	n.a.4	n.a.4	(+)	0	n.a.4	n.a. 4	n.a.4	n.a.4	NO
Macroscopic properties	3	b.p.		YES	NO	NO	-	-	n.a.5	-	-	>	YES	NO	n.a.4	n.a.4	-	0	n.a.4	n.a.4	n.a.4	n.a.4	NO	YES	NO	NO	+	-	n.a.5	+	+	<	YES
		TH-I		NO	n.a.4	n.a.4	-	0	n.a.4	n.a.4	n.a.4	n.a.4	NO	NO	n.a.4	n.a.4	-	0	n.a.4	n.a.4	n.a.4	n.a.4	NO	NO	n.a.4	n.a.4	+	0	n.a.4	n.a. 4	n.a.4	n.a.4	NO
		TH-II		YES	YES	YES	0	-	MIN	-	+	(=)	YES	NO	n.a.4	n.a.4	-	0	n.a.4	n.a.4	n.a.4	n.a.4	NO	NO	n.a.4	n.a.4	+	0	n.a.4	n.a. 4	n.a.4	n.a.4	NO
		TH-III		YES	YES	NO	-	+	n.a.8	unkn own	(-)	<<	YES	NO	n.a.4	n.a.4	+	0	n.a.4	n.a.4	n.a.4	n.a.4	NO	YES	YES	YES	-	+	MAX	+	-	<<	YES

Where n.a.4 means no conclusion can be drawn for the quadratic effect of a given factor as it is zero; n.a.5 means no extreme because the quadratic effect is smaller than ¼ of the linear effect; M.E. is the microcavitation effect; n.a.8 means no clear evidence for the existence of an extreme

materials MDPI

S.2. Additional information concerning DOE analysis

S.2.1. Preform material

From Figure 1a and Table S.1, the following conclusions can be drawn regarding the changes in the density of the preform due to an increase in rPET content:

a) **The linear main effect at a 5% probability of a type I error**: Regarding the mean values, an increase in rPET content in the preform results in an increase in the density of the preform material.
b) **The quadratic main effect at a 5% probability of a type I error**: Quadratic effects do not occur for any dependent variables. So, the influence of rPET content on the microstructure of the preform material is linear. Unfortunately, the statistical power of the test is low, and the probability of making a type II error is not within an acceptable range. As a result, it is highly probable that the conclusion about the lack of quadratic effects of rPET content on the physical and thermal properties of the preform material is incorrect.

It should be noted that the statistical power of the tests in almost all cases, especially for the analysis of quadratic effects, is less than 80%. Therefore, the results obtained cannot be used for quantitative analysis but can be employed for a preliminary qualitative analysis.

S.2.2. Bottle material relative to preform material (SBM process)

Figure 1b and Tables S.2 and S.3 show the following conclusions regarding the influence of the SBM process and rPET content on the bottle material in relation to the preform material's density, crystallinity:

a) **The two-factor linear interaction effects at a 5% probability of a type I error (for a 5% probability of making a type I error, Table S.2)**: There is a two-factor interaction between rPET content in the preform and the SBM process for density (for the “ALL” and “RPET” research), the effect interaction is negative and smaller than the absolute value for the effect of the SBM process and is comparable to the absolute value for the effect of the rPET content.
b) **The linear main effect at a 5% probability of a type I error (for a 5% probability of making a type I error, Table S.2):** For the “ALL” and “RPET” research, in terms of average values, the SBM process itself and the increase in rPET content in the preform affect the increase in density of the preform material. However, for this microstructural feature, there is a two-factor interaction effect for the SBM process and rPET content in the preform. It is comparable to or smaller than the absolute value of both the effect of the SBM process and rPET content. In the SBM process, the density increases for the bottle relative to the preform, while the increase in density is greater for zero rPET content than for 50% rPET content in the preform; the material density increases for the preform as the rPET content increases, while the bottle material density decreases, or does not change, as the rPET content in the preform increases. It should be noted that for the density, there is a two-way interaction effect for the SBM process and rPET content. For 0% and 50% rPET content in the SBM process, the density increases for the bottle relative to the preform (which is caused by an increase in the degree of molecular order, including an increase in the degree of crystallinity), while the increase in density is greater for 0% rPET content than for 50% content rPET. The material density increases for the preform as the rPET content increases, while the bottle material density does not

change (or possibly decreases because the effect of the two-factor interaction is slightly greater than the main effect on rPET content, the adopted methodology (see Equation (25) in Part I) for interpreting the equality relationship between individual effects neglects the influence of small differences between the effects) as the rPET content increases. However, increasing the lamp power causes an increase in the linear impact of rPET content on the bottle material density, while increasing the power of cooling fans reduces the linear impact of rPET content on the bottle material density.

c) **The quadratic main effect at a 5% probability of a type I error (for a 5% probability of making a type I error, Table S.3)**: Quadratic effects occur for density (for the "ALL" research plan). The quadratic effect is negative and, in absolute value, it is less than ¼ of the positive linear effect, so there is no minimum effect in terms of variability of rPET content. An increase in the low-range rPET content will result in a mild increase in the density of the material up to a certain rPET content, beyond which the growth trend is accelerating, and the increased rPET content will cause a more rapid increase in the density of the material.

From analyzing the impact of the power of heating lamps and fans on density, it can be concluded that increasing the power of heating lamps increases the impact of rPET content on the density of the material, while increasing the power of fans reduces the impact of rPET content on the density of the material. In the case of quadratic effects, the analysis of the impact of the power of heating lamps and fans indicates that increasing the power of heating lamps and the power of the cooling fans reduces the impact of RPET content on the nonlinearity for density.

However, the power of the tests for almost all statistically insignificant effects is less than 80% (Figure 1b). The results obtained cannot be used for quantitative analysis but can be employed for a preliminary qualitative analysis.

S.2.3. Bottle material

Figure 1c and Tables S4, S5, and S6 show the following conclusions regarding changes in the bottle's density as a result of changes in rPET content, the power of heating lamps, and the power of cooling fans in the CCF plan:

a) **The two-factor linear interaction effects at a 5% probability of a type I error (for a 5% probability of making a type I error, Table S.4)**: There is a two-factor interaction between rPET content and the power of heating lamps for density (the interaction effect is positive and greater than the absolute value for the rPET content effect and is smaller than absolute value for the effect on the power of heating lamps).

b) **The linear main effect at a 5% probability of a type I error (for a 5% probability of making a type I error, Table S.5):** As for the average values, increasing rPET content reduces the density of the material; increasing the power of heating lamps increases the density of the material while increasing the power of fans reduces the density of the bottle material. However, two-way interactions between all independent variables affect the density of the bottle material. The two-factor interaction between rPET content and the power of heating lamps shows that an increase in rPET content for low-power heating lamps results in a decrease in density, while an increase in rPET content for high-power heating lamps results in an increase in density, with an upward trend for high-power heating lamps greater than the decreasing trend for low-power heating lamps. On the other hand,

increasing the heating lamp power for both zero rPET content and 50% rPET content results in an increase in density, with the increasing trend being greater for high rPET content. The two-factor interaction between rPET content and the fan power shows that an increase in rPET content for low fan power results in a decrease in density and for high fan power results in a no change in density. On the other hand, an increase in fan power for zero rPET content as well as for 50% rPET content also causes a decrease in density, with the decreasing trend being greater for zero rPET content. The two-factor interaction between the power of heating lamps and the power of fans shows that an increase in the power of heating lamps for low fan power and for high fan power results in an increase in density, with the upward trend being stronger for high fan power. On the other hand, an increase in fan power for low-power heating lamps results in a decrease in density, while an increase in fan power for high-power heating lamps results in an increase in density, with the increasing trend for high-power heating lamps being stronger than the decreasing trend for low-power heating lamps.

c) **The quadratic main effect at a 5% probability of a type I error (for a 5% probability of making a type I error, Table S6):** In the case of bottle material density, the quadratic effects are statistically significant for all independent variables. For rPET content, the quadratic effect is positive and, in absolute value, it is greater than ½ of the negative linear effect, so there is a maximum effect in terms of variability of rPET content in the preform. An increase in the low-range rPET content will result in a gentle increase in the density of the material up to a certain rPET content, after which the trend changes and an increase in rPET content will result in a sharp decrease in the density of the material. For the power of heating lamps, the quadratic effect is positive and, in absolute value, it is less than ¼ of the positive linear effect, so there is no maximum effect in terms of variability of the power of heating lamps. An increase in the low-range power of heating lamps will result in a sharp increase in the density of plastic up to a certain rPET content, beyond which the increased power of heating lamps will cause a gentle increase in the density of the plastic. For the fan power, the quadratic effect is positive and, in absolute value, it is greater than ½ of the negative linear effect, so there is a maximum effect in the range of fan power variability. An increase in the low-range fan power will result in a gentle increase in the density of the material up to a certain fan power, beyond which the trend changes, and the increased fan power will cause a sharp decrease in the density of the material.

However, the power of tests for almost all statistically insignificant effects is less than 80% (Figure 1c), particularly for measurements of the degree of crystallinity (for which the power of tests is very low even for statistically significant effects) and for the microcavitation process (which is probably due to the low power of tests for degree of crystallinity). Therefore, the results obtained cannot be used for quantitative analysis but can be employed for a preliminary qualitative analysis.

S.5. The calculated p-values, power of ANOVA, and adjusted R^2 values for every experimental design

The calculated p-values and power of ANOVA for every standardized effect (s.effect) for each experimental design of each dependent variable are presented in Table S.7. The calculated adjusted R^2 (aR^2) values (the parameter of the fit of the model to the measured data) for each experimental design of

each dependent variable are also presented in Table S.7. It was assumed that the analyzed model is acceptably fitted for R^2 > 0.8.

Table S.7 uses four colors for the analysis of the p-value (statistical significance of a given effect in the analyzed model) versus the power of the ANOVA test (the higher the power of the test, the greater the probability that a given statistically insignificant effect is actually statistically insignificant) for every standardized effect (s.effect), the meaning of which is explained in Table S.8. Table S.7 also uses three colors for the value of the R^2 parameter (the percentage of the variance explained by the model in the measured data), the meaning of which is explained in Table S.9.

From Tables S.7, S.8, and S.9, it follows that from the analysis of p-values, ANOVA test power, and aR^2, it can be concluded that the adopted statistical models for all experimental designs for almost all dependent variables can only be used for preliminary qualitative analysis.

Table S.7. The calculated standardized effects (s.effect), p-values, power, and adjusted R^2 (aR^2) values for every experimental design (Table A1). The colors are explained in Tables S.8 and S.9.

Figure	Feature	Statistics	preform		bottle vs preform "ALL"					bottle								
			Table A1(b)		"ALL" - Table A1(c); "RPET" - Table A1(d)					Table A1(a)								
			(1)RPET content (L)	(1)RPET content (Q)	Type: ALL/RPET	(1)FORM (L)	(2)RPET content (L)	(2)RPET content (Q)	1L vs 2L	(1)RPET content (L)	(1)RPET content(Q)	(2)power of heating lamps (L)	(2)power of heating lamps (Q)	(3)power of cooling fans (L)	(3)power of cooling fans (Q)	1L vs 2L	1L vs 3L	2L vs3L
Figure 1	Density	s.effect	4.92	-1.63	ALL	47.89	8.16	-3.12	-8.56	-2.62	5.14	18.34	4.34	-4.45	4.74	7.03	2.34	6.44
					RPET	27.06	4.64	-1.58	-4.64									
		p-value	0.00	0.13	ALL	0.00	0.00	0.00	0.00	0.01	0.00	0.00	0.00	0.00	0.00	0.00	0.02	0.00
					RPET	0.00	0.00	0.12	0.00									
		power	0.86	0.19	ALL	1.00	0.99	0.39	1.00	0.61	1.00	1.00	0.99	0.97	1.00	1.00	0.51	1.00
					RPET	1.00	0.20	0.08	0.97									
		aR^2	0.82		ALL	0.98				0.92								
					RPET	0.99												
	Crystallinity from DSC	s.effect	3.27	0.00	ALL	52.22	1.75	-0.53	-1.40	0.39	-0.79	1.33	-0.69	-0.43	-1.29	0.12	0.28	1.56
					RPET	54.37	3.84	-0.32	0.50									
		p-value	0.02	1.00	ALL	0.00	0.01	0.58	0.00	0.70	0.34	0.19	0.41	0.67	0.13	0.90	0.78	0.13
					RPET	0.00	0.00	0.74	0.00									
		power	0.14	0.05	ALL	1.00	0.20	0.06	1.00	0.06	0.09	0.13	0.08	0.06	0.17	0.05	0.05	0.17
					RPET	1.00	0.18	0.05	0.98									
		aR^2	0.64		ALL	0.98				0.17								
					RPET	0.99												
		s.effect	-3.01	1.63	ALL	15.64	-2.62	1.25	3.24	0.79	-1.46	-0.79	-1.25	0.05	-1.88	-0.73	0.05	0.93

	**Microcavitation effect**				RPET	11.04	-0.71	1.02	3.83									
		**p-value**	0.02	0.13	ALL	0.00	0.02	0.19	0.00	0.44	0.09	0.44	0.14	0.96	0.03	0.47	0.96	0.36
					RPET	0.00	0.62	0.30	0.00									
		**power**	0.72	0.31	ALL	1.00	0.98	0.58	1.00	0.74	1.00	0.74	1.00	0.05	1.00	0.67	0.05	0.67
					RPET	1.00	0.06	0.12	1.00									
		**aR^2**	0.67		ALL	0.77				0.19								
					RPET	0.86												
	**Intrinsic viscosity**	**s.effect**	-0.79	0.09	ALL	2.70	-1.83	-1.15	-1.01	-3.91	-2.20	1.24	0.90	-0.18	2.03	-1.69	2.10	-0.13
					RPET	6.19	-4.88	-2.30	-4.04									
		**p-value**	0.46	0.93	ALL	0.00	0.08	0.23	0.00	0.00	0.01	0.22	0.29	0.86	0.02	0.10	0.04	0.90
					RPET	0.00	0.00	0.03	0.00									
		**power**	0.20	0.05	ALL	1.00	1.00	0.97	1.00	1.00	1.00	0.85	0.75	0.07	1.00	0.98	1.00	0.06
					RPET	1.00	0.95	0.61	1.00									
		**aR^2**	0.10		ALL	0.24				0.45								
					RPET	0.78												
	**Glass transition temperature**	**s.effect**	0.35	-0.03	ALL	-0.92	0.60	0.00	-0.36	0.34	0.10	2.21	-0.42	-1.52	0.49	-0.55	-0.13	-2.08
					RPET	-0.67	-0.16	0.16	-0.79									
		**p-value**	0.74	0.98	ALL	0.32	0.09	1.00	0.42	0.74	0.91	0.03	0.62	0.14	0.56	0.58	0.90	0.04
					RPET	0.59	0.58	0.87	0.39									
		**power**	0.08	0.05	ALL	0.96	1.00	0.05	0.86	0.19	0.07	1.00	0.35	1.00	0.46	0.41	0.07	1.00
					RPET	0.22	0.24	0.07	0.50									
		**aR^2**	0.02		ALL	0.01				0.08								
					RPET	0.07												
	**Melting temperature**	**s.effect**	0.62	0.15	ALL	0.19	0.07	0.81	-0.72	-0.72	0.99	-0.95	0.43	0.11	0.51	-0.43	-0.44	1.11
					RPET	0.42	0.01	0.12	-0.66									
		**p-value**	0.56	0.88	ALL	0.83	0.71	0.39	0.35	0.48	0.24	0.35	0.61	0.91	0.54	0.67	0.66	0.27
					RPET	0.74	0.94	0.90	0.52									

			**power**	0.14	0.06	ALL	0.12	0.28	0.88	0.93	0.70	0.98	0.91	0.43	0.07	0.57	0.31	0.33	0.97
						RPET	0.12	0.05	0.06	0.30									
			**aR^2**	0.06		ALL	0.02				0.11								
						RPET	0.04												
**Figure 2**	**τ_2 (mean)**	**with σ_3**	**s.effect**	-2.31	-0.71	ALL	-15.23	-0.74	-0.37	0.23	-0.57	-0.24	0.67	0.58	0.13	1.01	0.72	-1.61	0.67
						RPET	-13.37	-0.42	-0.73	0.52									
			**p-value**	0.06	0.48	ALL	0.00	0.48	0.70	0.00	0.57	0.77	0.51	0.49	0.90	0.23	0.48	0.12	0.51
						RPET	0.00	0.76	0.45	0.00									
			**power**	0.66	0.12	ALL	1.00	0.20	0.09	1.00	0.49	0.17	0.61	0.65	0.07	0.98	0.68	1.00	0.61
						RPET	1.00	0.05	0.07	0.98									
			**aR^2**	0.50		ALL	0.81				0.16								
						RPET	0.92												
		**without σ_3**	**s.effect**	-8.38	-4.50	ALL	-22.56	-0.59	-0.18	0.68	0.10	0.43	0.40	0.57	-1.38	1.80	1.54	-1.66	0.23
						RPET	-23.64	-0.66	-1.10	0.78									
			**p-value**	0.00	0.00	ALL	0.00	0.57	0.85	0.00	0.92	0.61	0.69	0.50	0.18	0.04	0.13	0.11	0.82
						RPET	0.00	0.63	0.26	0.00									
			**power**	0.85	0.41	ALL	1.00	0.10	0.06	1.00	0.06	0.37	0.24	0.58	0.99	1.00	1.00	1.00	0.11
						RPET	1.00	0.05	0.07	0.99									
			**aR^2**	0.94		ALL	0.90				0.06								
						RPET	0.97												
	**τ_2 (fitting uncertainty)**	**with σ_3**	**s.effect**	-3.40	0.02	ALL	5.52	-4.00	-0.55	2.30	-2.65	-1.16	-2.12	1.25	1.59	-1.97	-2.07	-3.26	-0.89
						RPET	5.45	-5.30	0.10	1.03									
			**p-value**	0.01	0.99	ALL	0.00	0.00	0.56	0.00	0.01	0.17	0.04	0.14	0.12	0.02	0.05	0.00	0.38
						RPET	0.00	0.00	0.92	0.00									
			**power**	0.82	0.05	ALL	1.00	1.00	0.35	1.00	1.00	0.93	1.00	0.96	0.97	1.00	1.00	1.00	0.59
						RPET	0.99	0.95	0.05	1.00									
			**aR^2**	0.66		ALL	0.43				0.43								

						RPET	0.76												
		without σ_3	**s.effect**	-2.86	-1.84	ALL	-2.96	-3.51	-2.95	2.31	-1.59	-0.76	-1.94	0.31	0.53	1.25	-1.09	0.49	-1.28
						RPET	-1.28	-2.85	-2.05	2.02									
			**p-value**	0.03	0.10	ALL	0.00	0.00	0.00	0.01	0.12	0.36	0.06	0.71	0.60	0.14	0.28	0.62	0.21
						RPET	0.32	0.07	0.05	0.01									
			**power**	0.68	0.38	ALL	1.00	1.00	1.00	1.00	1.00	0.81	1.00	0.21	0.37	1.00	0.92	0.33	0.98
						RPET	0.16	0.47	0.53	0.75									
			**aR^2**	0.67		ALL	0.21				0.10								
						RPET	0.43												
	τ_3 (mean)	with σ_3	**s.effect**	-2.26	2.18	ALL	-1.93	-1.14	-0.03	-0.44	-2.16	-0.86	-0.23	1.83	3.08	-0.03	0.05	-2.81	-0.27
						RPET	-0.43	-1.27	-0.26	-0.46									
			**p-value**	0.06	0.06	ALL	0.04	0.25	0.97	0.11	0.04	0.31	0.82	0.03	0.00	0.97	0.96	0.01	0.79
						RPET	0.73	0.29	0.78	0.42									
			**power**	0.52	0.54	ALL	1.00	0.98	0.05	1.00	1.00	0.79	0.09	1.00	1.00	0.05	0.05	1.00	0.11
						RPET	0.11	0.60	0.09	0.39									
			**aR^2**	0.63		ALL	0.10				0.33								
						RPET	0.20												
		without σ_3	**s.effect**	-41.03	35.71	ALL	-10.11	-1.55	2.03	0.16	-2.33	2.27	-1.09	3.01	2.97	1.03	2.10	-4.20	-4.22
						RPET	-15.07	-3.18	0.78	-0.18									
			**p-value**	0.00	0.00	ALL	0.00	0.12	0.04	0.00	0.03	0.01	0.28	0.00	0.01	0.22	0.04	0.00	0.00
						RPET	0.00	0.02	0.43	0.00									
			**power**	0.76	0.69	ALL	1.00	0.91	0.99	1.00	1.00	1.00	0.64	1.00	1.00	0.75	0.99	1.00	1.00
						RPET	1.00	0.26	0.07	0.94									
			**aR^2**	1.00		ALL	0.67				0.58								
						RPET	0.94												
	τ_3 (fitting	with σ_3	**s.effect**	-1.34	-0.45	ALL	4.56	-2.09	0.08	-0.42	-3.27	0.77	-1.83	-0.27	2.02	-0.31	-1.52	-2.50	0.18
						RPET	4.26	-1.91	-0.32	-0.11									

			**p-value**	0.23	0.65	ALL	0.00	0.05	0.94	0.00	0.00	0.36	0.08	0.74	0.05	0.71	0.14	0.02	0.86
						RPET	0.00	0.20	0.74	0.01									
			**power**	0.40	0.09	ALL	1.00	1.00	0.06	1.00	1.00	0.71	1.00	0.14	1.00	0.18	0.99	1.00	0.08
						RPET	1.00	0.44	0.07	0.98									
			**aR^2**	0.25		ALL	0.37				0.31								
						RPET	0.54												
		**without σ_3**	**s.effect**	1.57	-5.96	ALL	1.41	-0.57	-1.80	-1.41	-2.50	0.53	-1.97	1.55	1.83	-0.17	-0.79	-0.47	-1.18
						RPET	2.97	0.10	-2.22	-1.07									
			**p-value**	0.17	0.00	ALL	0.13	0.59	0.06	0.03	0.02	0.53	0.06	0.07	0.08	0.84	0.44	0.64	0.25
						RPET	0.03	0.95	0.03	0.11									
			**power**	0.14	0.92	ALL	1.00	0.44	1.00	1.00	1.00	0.46	1.00	1.00	1.00	0.09	0.63	0.28	0.93
						RPET	0.87	0.05	0.85	0.60									
			**aR^2**	0.88		ALL	0.15				0.20								
						RPET	0.57												
	**$I_1 + I_3$ (mean)**	**with σ_3**	**s.effect**	1.52	-0.73	ALL	-16.56	0.92	1.05	0.22	1.49	2.19	-1.37	-0.94	-2.62	0.67	0.36	2.19	-0.54
						RPET	-20.26	0.00	0.67	-0.79									
			**p-value**	0.18	0.47	ALL	0.00	0.08	0.27	0.00	0.14	0.01	0.18	0.26	0.01	0.42	0.72	0.04	0.59
						RPET	0.00	1.00	0.49	0.00									
			**power**	0.45	0.15	ALL	1.00	0.78	0.39	1.00	0.98	1.00	0.96	0.87	1.00	0.60	0.17	1.00	0.32
						RPET	1.00	0.05	0.06	0.99									
			**aR^2**	0.33		ALL	0.83				0.30								
						RPET	0.96												
		**without σ_3**	**s.effect**	2.31	-1.05	ALL	-31.60	0.50	0.47	-0.73	-0.28	1.35	-2.29	-0.22	-1.03	-0.09	-0.46	1.75	1.09
						RPET	-36.14	-0.91	0.65	-2.27									
			**p-value**	0.06	0.31	ALL	0.00	0.34	0.62	0.00	0.78	0.11	0.03	0.79	0.31	0.91	0.65	0.09	0.28
						RPET	0.00	0.06	0.50	0.00									
			**power**	0.64	0.21	ALL	1.00	0.12	0.07	1.00	0.14	1.00	1.00	0.13	0.89	0.06	0.30	1.00	0.92

						RPET	1.00	0.08	0.05	0.99									
			**aR^2**	0.52		ALL	0.95				0.11								
						RPET	0.99												
	**$I_1 + I_3$ (fitting uncertainty)**	**with σ_3**	**s.effect**	-1.47	-0.42	ALL	5.23	-1.72	0.10	0.47	-1.62	0.90	0.38	0.61	0.67	0.90	-1.12	-3.25	-1.65
						RPET	6.87	-2.86	-0.47	-0.32									
			**p-value**	0.19	0.67	ALL	0.00	0.10	0.91	0.00	0.11	0.28	0.71	0.46	0.51	0.28	0.27	0.00	0.11
						RPET	0.00	0.05	0.63	0.00									
			**power**	0.44	0.09	ALL	1.00	1.00	0.06	1.00	1.00	0.89	0.20	0.58	0.50	0.89	0.91	1.00	1.00
						RPET	1.00	0.53	0.08	1.00									
			**aR^2**	0.28		ALL	0.38				0.19								
						RPET	0.76												
		**without σ_3**	**s.effect**	0.04	-0.71	ALL	-0.06	-0.88	-1.08	-0.96	-2.50	0.07	-2.08	1.35	1.15	0.35	-0.50	0.18	-1.86
						RPET	0.84	-0.32	-0.85	-0.40									
			**p-value**	0.97	0.48	ALL	0.95	0.41	0.26	0.09	0.02	0.94	0.04	0.11	0.26	0.67	0.62	0.86	0.07
						RPET	0.51	0.82	0.38	0.54									
			**power**	0.05	0.18	ALL	0.06	0.84	0.98	1.00	1.00	0.06	1.00	1.00	0.92	0.24	0.31	0.08	1.00
						RPET	0.29	0.08	0.46	0.25									
			**aR^2**	0.09		ALL	0.03				0.34								
						RPET	0.16												
	**I_2 (mean)**	**with σ_3**	**s.effect**	-1.52	0.73	ALL	16.56	-0.92	-1.05	-0.22	-1.49	-2.19	1.37	0.94	2.62	-0.67	-0.36	-2.19	0.54
						RPET	20.26	0.00	-0.67	0.79									
			**p-value**	0.18	0.47	ALL	0.00	0.01	0.27	0.00	0.14	0.01	0.18	0.26	0.01	0.42	0.72	0.04	0.59
						RPET	0.00	1.00	0.49	0.00									
			**power**	0.45	0.15	ALL	1.00	0.97	0.39	1.00	0.98	1.00	0.96	0.87	1.00	0.60	0.17	1.00	0.32
						RPET	1.00	0.05	0.06	0.99									
			**aR^2**	0.33		ALL	0.83				0.30								
						RPET	0.96												

		**without σ_3**	**s.effect**	-2.31	1.05	ALL	31.60	-0.50	-0.47	0.73	0.28	-1.35	2.29	0.22	1.03	0.09	0.46	-1.75	-1.09
						RPET	36.14	0.91	-0.65	2.27									
			**p-value**	0.06	0.31	ALL	0.00	0.16	0.62	0.00	0.78	0.11	0.03	0.79	0.31	0.91	0.65	0.09	0.28
						RPET	0.00	0.01	0.50	0.00									
			**power**	0.64	0.21	ALL	1.00	0.22	0.07	1.00	0.14	1.00	1.00	0.13	0.89	0.06	0.30	1.00	0.92
						RPET	1.00	0.11	0.05	0.99									
			**aR^2**	0.52		ALL	0.95				0.11								
						RPET	0.99												
	**I_2 (fitting uncertainty)**	**with σ_3**	**s.effect**	-1.47	-0.42	ALL	5.23	-1.72	0.10	0.47	-1.62	0.90	0.38	0.61	0.67	0.90	-1.12	-3.25	-1.65
						RPET	6.87	-2.86	-0.47	-0.32									
			**p-value**	0.19	0.67	ALL	0.00	0.10	0.91	0.00	0.11	0.28	0.71	0.46	0.51	0.28	0.27	0.00	0.11
						RPET	0.00	0.05	0.63	0.00									
			**power**	0.44	0.09	ALL	1.00	1.00	0.06	1.00	1.00	0.89	0.20	0.58	0.50	0.89	0.91	1.00	1.00
						RPET	1.00	0.53	0.08	1.00									
			**aR^2**	0.28		ALL	0.38				0.19								
						RPET	0.76												
		**without σ_3**	**s.effect**	0.04	-0.71	ALL	-0.06	-0.88	-1.08	-0.96	-2.50	0.07	-2.08	1.35	1.15	0.35	-0.50	0.18	-1.86
						RPET	0.84	-0.32	-0.85	-0.40									
			**p-value**	0.97	0.48	ALL	0.95	0.41	0.26	0.09	0.02	0.94	0.04	0.11	0.26	0.67	0.62	0.86	0.07
						RPET	0.51	0.82	0.38	0.54									
			**power**	0.05	0.18	ALL	0.06	0.84	0.98	1.00	1.00	0.06	1.00	1.00	0.92	0.24	0.31	0.08	1.00
						RPET	0.29	0.08	0.46	0.25									
			**aR^2**	0.09		ALL	0.03				0.18								
						RPET	0.16												
	**σ_3 (mean)**		**s.effect**	0.32	2.46	ALL	9.19	0.10	2.17	-0.06	0.07	2.71	0.34	-1.46	-3.40	-0.65	1.82	2.96	-2.31
						RPET	9.52	0.43	1.72	0.21									
			**p-value**	0.76	0.04	ALL	0.00	0.92	0.03	0.00	0.94	0.00	0.74	0.09	0.00	0.44	0.08	0.01	0.03

						RPET	0.00	0.75	0.09	0.00									
			**power**	0.06	0.75	ALL	0.05	0.05	0.05	0.05	0.05	1.00	0.13	0.98	1.00	0.45	0.99	1.00	1.00
						RPET	1.00	0.06	0.30	0.98									
			**aR^2**	0.54		ALL	0.63				0.48								
						RPET	0.84												
	**σ₃ (standard uncertainty)**		**s.effect**	-0.89	-0.51	ALL	4.10	-1.81	-0.11	-0.68	-3.23	0.55	-1.60	-0.37	2.35	-0.56	-1.03	-2.42	0.12
						RPET	9.52	0.43	1.72	0.21									
			**p-value**	0.41	0.61	ALL	0.00	0.09	0.90	0.00	0.00	0.51	0.12	0.66	0.02	0.50	0.31	0.02	0.90
						RPET	0.01	0.32	0.64	0.03									
			**power**	0.22	0.11	ALL	1.00	1.00	0.06	1.00	1.00	0.45	0.99	0.23	1.00	0.47	0.81	1.00	0.06
						RPET	0.99	0.33	0.11	0.96									
			**aR^2**	0.15		ALL	0.33				0.29								
						RPET	0.43												
**Figure 3**	**Thickness profiles (Figure 2b [17])**	**I [µm]**	**s.effect**	-	-	-	-	-	-	-	-8.18	-0.62	-19.42	-0.45	13.11	-0.01	3.66	3.68	-0.73
			**p-value**	-	-	-	-	-	-	-	0.00	0.72	0.00	0.79	0.00	0.83	0.17	0.57	0.46
			**power**	-	-	-	-	-	-	-	1.00	0.17	1.00	0.11	1.00	0.09	0.96	0.35	0.52
			**aR^2**	-	-	-	-	-	-	-	0.35								
		**II [µm]**	**s.effect**	-	-	-	-	-	-	-	1.63	-1.79	-10.39	2.15	5.23	2.21	-2.03	5.60	-4.51
			**p-value**	-	-	-	-	-	-	-	0.28	0.00	0.00	0.65	0.00	0.28	0.00	0.14	1.00
			**power**	-	-	-	-	-	-	-	0.28	1.00	1.00	0.09	1.00	0.28	0.98	0.47	0.05
			**aR^2**	-	-	-	-	-	-	-	0.26								
		**III [µm]**	**s.effect**	-	-	-	-	-	-	-	-17.28	12.83	7.78	-1.15	-9.53	11.50	14.74	0.07	19.58
			**p-value**	-	-	-	-	-	-	-	0.00	0.00	0.00	0.81	0.00	0.00	0.00	0.43	0.00
			**power**	-	-	-	-	-	-	-	1.00	1.00	1.00	0.06	1.00	1.00	1.00	0.20	1.00
			**aR^2**	-	-	-	-	-	-	-	0.70								
	**Pressure resistanc**	**Burst pressure**	**s.effect**	-	-	-	-	-	-	-	-17.21	-3.17	-24.03	-0.45	14.07	-3.76	1.27	0.63	-1.09
			**p-value**	-	-	-	-	-	-	-	0.00	0.00	0.00	0.59	0.00	0.00	0.20	0.52	0.28

			**power**	-	-	-	-	-	-	-	1.00	1.00	1.00	0.13	1.00	1.00	0.49	0.16	0.38
			**aR^2**	-	-	-	-	-	-	-	0.89								

Table S.8. Explanation of four colors for the analysis of the p-value (statistical significance of a given effect in the analyzed model) versus the power of the ANOVA test (the higher the power of the test, the greater the probability that a given statistically insignificant effect is actually statistically insignificant) used in table S.7.

The analysis of the p-value versus the power of the ANOVA test.			The higher the power of the test, the greater the probability that a given statistically insignificant effect is actually statistically insignificant.	
			**power**	
			**power > 0.8**	**power <=0.8**
Statistical significance of a given effect in the analyzed model.	**p-value**	**p < 0.05**	statistically significant effect	statistically significant effect
			The model can be used for quantitative and qualitative analysis.	The model can only be used for qualitative analysis.
		**p >= 0.05**	statistically insignificant effect, with high confidence that the effect is statistically insignificant	statistically insignificant effect, with low confidence that the effect is statistically insignificant
			The model can be used for quantitative and qualitative analysis.	The model can only be used for preliminary qualitative analysis.

Table S.9. Explanation of three colors for the analysis of the value of the R^2 parameter used in table S.7.

R^2 - the percentage of the variance explained by the model in the measured data		
**adjusted R^2** [illegible]	**0.8 - 1.0**	more than 80% of the variance of the measurement data is explained by the adopted model
		The model can be used for quantitative and qualitative analysis.
	**0.6 - 0.8**	more than 50% but less than 80% of the variance of the measurement data is explained by the adopted model
		The model can only be used for qualitative analysis.
	**0 - 0.6**	less than 50% of the variance of the measurement data is explained by the adopted model
		The model can only be used for preliminary qualitative analysis.
